# Discovery of New Pyrrolo[2,3-*d*]pyrimidine Derivatives as Potential Multi-Targeted Kinase Inhibitors and Apoptosis Inducers

**DOI:** 10.3390/ph16091324

**Published:** 2023-09-19

**Authors:** AbdulAziz A. Alotaibi, Mohammed M. Alanazi, A. F. M. Motiur Rahman

**Affiliations:** Department of Pharmaceutical Chemistry, College of Pharmacy, King Saud University, Riyadh 11451, Saudi Arabia; 442106443@student.ksu.edu.sa

**Keywords:** Pyrrolo[2,3-*d*]pyrimidine, apoptosis inducer, tyrosine kinase inhibitor, multiple kinase inhibitor

## Abstract

In the pursuit of developing more potent and effective targeted kinase inhibitors (TKIs), a series of new compounds, specifically halogenated ‘(*E*)-4-((7*H*-pyrrolo[2,3-*d*]pyrimidin-4-yl)amino)-*N*’-benzylidenebenzohydrazides’, were successfully synthesized in three steps with high yields. Among these novel compounds, namely **5e**, **5h**, **5k**, and **5l**, promising cytotoxic effects were observed against four different cancer cell lines, with IC_50_ values ranging from 29 to 59 µM. Notably, compound **5k** emerged as the most potent inhibitor, exhibiting significant activity against EGFR, Her2, VEGFR2, and CDK2 enzymes, with IC_50_ values ranging from 40 to 204 nM, comparable to the well-known TKI sunitinib (IC_50_ = 261 nM). Mechanistic investigations of compound **5k** revealed its ability to induce cell cycle arrest and apoptosis in HepG2 cells, accompanied by a notable increase in proapoptotic proteins caspase-3 and Bax, as well as the downregulation of Bcl-2 activity. Furthermore, molecular docking studies indicated similar binding interactions between compound **5k** and the four enzymes, as observed with sunitinib. These findings highlight the potential of compound **5k** as a promising candidate for further development as a multi-targeted kinase inhibitor with enhanced potency.

## 1. Introduction

**Figure 1:**
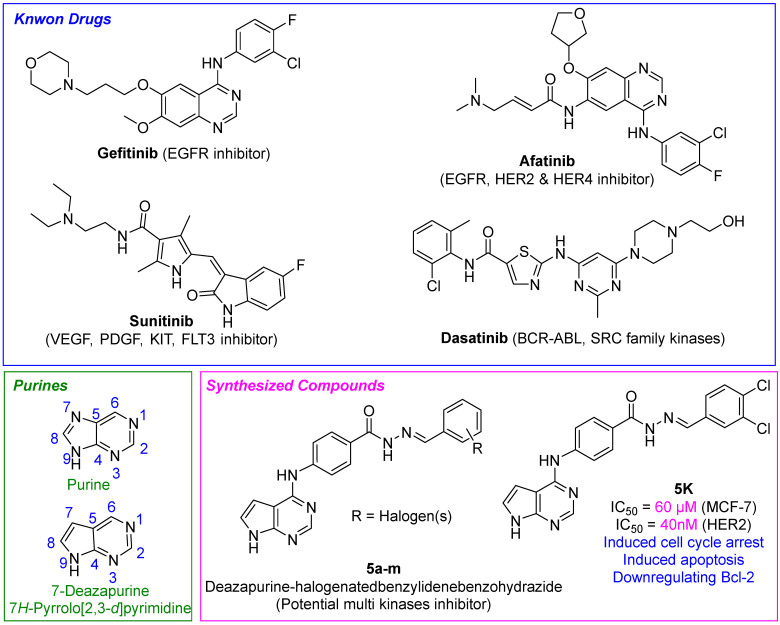
An example of a few known TKIs and synthesized compounds.

Cancer is a major health concern worldwide and the development of effective anticancer therapeutics remains a challenge. Halogenated TKIs are characterized by the presence of halogen atoms (such as fluorine, chlorine, bromine, or iodine) in their chemical structure. Halogen atoms are strategically incorporated into the organic molecule to enhance its potency, selectivity, and pharmacological properties [1]. The addition of halogen atoms can influence the binding affinity of the TKI to its target kinase, potentially leading to improved therapeutic effects [1,2,3]. Halogenated TKIs, such as gefitinib, dasatinib, and afatinib, have emerged as important classes of TKIs due to their ability to enhance the potency and selectivity of these drugs (Figure 1) [4,5,6,7]. Gefitinib is a TKI that targets the epidermal growth factor receptor (EGFR) and has been used in the treatment of non-small cell lung cancer (NSCLC) [5]. The halogen substituents on gefitinib have been shown to enhance its potency and selectivity, leading to improved efficacy in certain patients. One advantage of gefitinib is its oral bioavailability and relatively low toxicity compared to traditional chemotherapy agents, which can result in significant side effects. Additionally, gefitinib has shown promise in patients with EGFR mutations, which are commonly found in Asian populations and are associated with better response rates to TKIs. However, gefitinib has some limitations, such as the development of resistance and relatively limited efficacy in patients without EGFR mutations. Dasatinib is a TKI that targets multiple tyrosine kinases, including BCR-ABL, SRC family kinases, and c-KIT [6]. The halogen substituents on dasatinib have been shown to enhance its potency and selectivity, leading to improved efficacy in certain patients. One advantage of dasatinib is its broad spectrum of activity against multiple tyrosine kinases, which makes it a useful tool in the treatment of various cancers. In particular, dasatinib has shown efficacy in patients with chronic myeloid leukemia (CML) and acute lymphoblastic leukemia (ALL), where it has been approved for use as a first-line or second-line therapy. Another advantage of dasatinib is its ability to overcome resistance to other TKIs, including imatinib, which is commonly used in the treatment of CML. However, dasatinib has some limitations, such as potential adverse effects including fluid retention, bleeding, and pulmonary arterial hypertension (PAH), and high cost. Afatinib is a TKI that targets EGFR, HER2, and HER4 and has been used in the treatment of NSCLC and head and neck cancer [7]. The halogen substituents on afatinib have been shown to enhance its potency and selectivity, leading to improved efficacy in certain patients. One advantage of afatinib is its broad spectrum of activity against multiple HER family members, which makes it a useful tool in the treatment of various cancers. In particular, afatinib has shown efficacy in patients with EGFR mutations, where it has been approved for use as a first-line therapy. Another advantage of afatinib is its ability to overcome resistance to other EGFR TKIs, including gefitinib and erlotinib. However, afatinib has some limitations, such as potential adverse effects including diarrhea, rash, and mucositis, and high cost. Other TKIs, such as entrectinib [8], avitinib [9,10], saracatinib [11,12], ponatinib [6,13], foretinib [14], vandetanib [15], flumatinib [16], vemurafenib [17], sorafenib [18], cabozantinib [19], nilotinib [20], lapatinib [21,22], and selumetinib [23], all them having halogen substituents, have also shown efficacy in treating various cancers. However, these drugs may have potential adverse effects, such as fatigue, cognitive impairment, interstitial lung disease, gastrointestinal toxicity, neutropenia, thrombocytopenia, QT prolongation, hypertension, hand-foot syndrome, and photosensitivity. Additionally, some of these drugs have limitations, such as high cost and limited accessibility to patients in certain healthcare systems.

Sunitinib is a multi-targeted TKI that inhibits several receptor tyrosine kinases, including VEGF, PDGF, KIT, and FLT3, and has been used in the treatment of various malignancies [24,25]. The pyrrole moiety and halogen substituent on sunitinib have been shown to enhance its potency and selectivity, leading to improved efficacy in certain patients. One advantage of sunitinib is its broad spectrum of activity against multiple receptor tyrosine kinases, which makes it a useful tool in the treatment of various cancers. In particular, sunitinib has shown efficacy in patients with metastatic renal cell carcinoma and gastrointestinal stromal tumors. Another advantage of sunitinib is its ability to overcome resistance to other TKIs, including imatinib. However, sunitinib has some limitations, such as potential adverse effects including fatigue, hypertension, and hand-foot syndrome, as well as high cost.

Pyrrolo[2,3-*d*]pyrimidine, on the other hand, refers to a specific heterocyclic compound that contains a purine-like structure but lacks a nitrogen atom at the 7-position. It is structurally distinct from the traditional purine-based molecules. Pyrrolo[2,3-*d*]pyrimidine derivatives have received significant attention in drug discovery and development due to their potential as kinase inhibitors [26,27,28]. These compounds offer unique structural characteristics that can contribute to their binding affinity and selectivity for specific kinases. The incorporation of both Pyrrolo[2,3-*d*]pyrimidine and halogen substituents within the same molecules represents a strategic approach aimed at the development of groundbreaking tyrosine kinase inhibitors with enhanced potency, selectivity, and therapeutic efficacy. Our ongoing research aims to improve the efficacy of TKIs in the treatment of cancer by developing newer generation drugs with improved efficacy against resistant mutations, identifying biomarkers for personalized medicine, and optimizing the use of existing drugs to overcome limitations such as adverse effects and high cost. Additionally, efforts are being made to develop generic versions of these drugs and reduce the cost of therapy. Due to the intriguing structures and exceptional biological activities of 7-deazapurine-containing molecules [26,29,30], in this study, we report the synthesis and in-vitro cytotoxicity evaluation of a novel halogenated ‘(*E*)-4-((7*H*-Pyrrolo[2,3-*d*]pyrimidin-4-yl)amino)-*N*’-benzylidenebenzohydrazide’ compounds. These compounds were designed to target multiple tyrosine kinases and to exhibit potent anticancer activity. The synthesized compounds were evaluated for their cytotoxicity against a panel of cancer cell lines (four cell lines) and its ability to inhibit multiple tyrosine kinases.

## 2. Results and Discussion

### 2.1. Chemistry

**Scheme 1:**
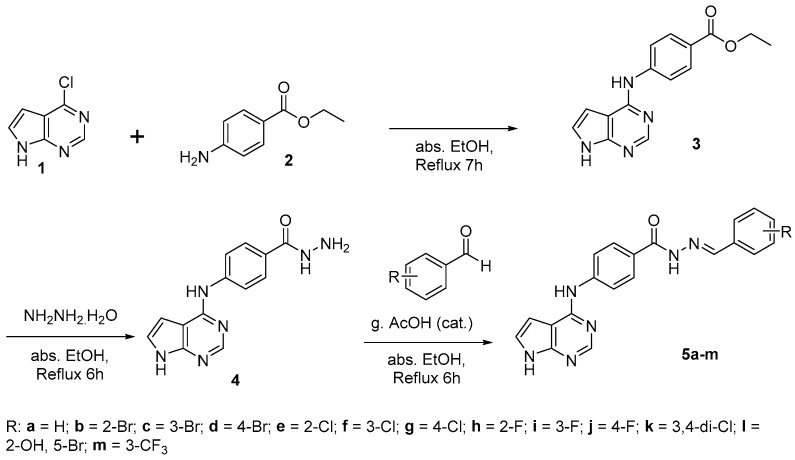
Synthesis of compounds **5a**–**m**.

Synthesis of Pyrrolo[2,3-*d*]pyrimidine-halogenatedbenzylidene-benzohydrazide derivatives was straightforward, as depicted in Scheme 1. In the first step, 6-chloro-7-deazapurine (**1**) was treated with benzocaine (**2**) in ethanol (absolute) at refluxing conditions overnight in order to obtain ester, ethyl 4-((7*H*-Pyrrolo[2,3-d]pyrimidin-4-yl)amino)benzoate (**3**), adopting a previously reported method [26,31] as a precursor for the synthesis of hydrazide (**4**). In the second step, ester **3** was converted to hydrazide (**4**) [31] by refluxing with hydrazine hydrate (80% in H_2_O) as a starting material for the preparation of targeted compounds (**5a**–**m**). In the final step, hydrazide **4** was reacted with various halogen substituted aldehydes to obtain Pyrrolo[2,3-*d*]pyrimidine-halogenatedbenzylidene-benzohydrazide derivatives (**5a**–**m**). The reaction was conducted by refluxing absolute ethanol containing a catalytic amount of glacial acetic acid (approximately 3–4 drops) overnight. The chemical structures of the synthesized compounds were confirmed through infrared spectroscopy (IR), mass spectrometry (MS), ^1^H-NMR, and ^13^C-NMR spectroscopy. Intermediate compounds’ structures were compared with the those reported values [31]. Physical properties (color and melting points) also were reported.

The proton located at the 2-position of Pyrrolo[2,3-*d*]pyrimidine displayed a distinctive signal within the chemical shift range of 8.34–8.38 ppm in all of the final compounds (**5a**–**m**), except for the ester **3** at 8.47 ppm and the hydrazide **4** at 8.33 ppm. Furthermore, the benzylidene protons of the compounds **5a**–**m** were also detected, offering valuable information for the structural confirmation of the final product. It is worth noting that the presence of substituents such as 2-bromo, 2-chloro, and 2-fluoro resulted in a downfield shift. Specifically, the bromo-substituted compound **5b** exhibited a shift of 0.38 ppm, the chloro-substituted compound **5e** exhibited a shift of 0.43 ppm, and the fluoro-substituted compound **5h** exhibited a shift of 0.26 ppm, relative to the non-substituted compound **5a**. The structures of all compounds **5a**–**m** were further confirmed through the observation of characteristic IR bands, such as the sharp peaks at 1604–1639 cm^−1^ for the benzylidene hydrazone (-N=CH-) group in all compounds. Additionally, each compound was characterized by its unique IR band, with bromo-substituted compounds (**5b**–**d, 5l**) exhibiting characteristic stretching bands at 720–760 cm^−1^, chloro-substituted compounds (**5e**–**g**, **5k**) exhibiting characteristic stretching bands at 521–750 cm^−1^, and fluoro-substituted compounds (**5h**–**j**, **5m**) demonstrating characteristic stretching bands at 512–529 cm^−1^. Finally, the structures of all compounds were confirmed through their mass spectral data.

### 2.2. Biological Evaluation

#### 2.2.1. In Vitro Cytotoxicity

The cytotoxic effects of compounds **5a**–**m** were evaluated using a standard MTT method against four different cancer cell lines, namely mammary gland cancer (MCF-7), hepatocellular carcinoma (HepG2), breast cancer (MDA-MB-231), and epithelioid cervix carcinoma (HeLa). The results of the study, presented in Table 1, indicate that the cytotoxicity of each compound was expressed as the concentration required to kill 50% of cancer cells and compared to that of sunitinib, a potent multi-kinase inhibitor approved by the FDA. In general, all synthesized derivatives exhibited different levels of cytotoxic effects. Notably, compounds **5e**, **5h**, **5k**, and **5**l demonstrated modest cytotoxic effects against all tested cancer cell lines, with IC_50_ values ranging from 29 to 59 µM. These findings highlight the potential of these compounds as candidates for further development as cytotoxic agents for cancer treatment.

#### 2.2.2. Structure Activity Relationship

The investigation focused on establishing a correlation between the structures and activities of compounds **5a**–**m** (Scheme 1). Notably, compound **5a** (Table 1, entry **5a**; Scheme 1), which featured an unsubstituted phenyl group, exhibited cytotoxicity levels between those of all the compounds tested. Its IC_50_ values against various cell lines ranged from 43.15 to 68.17 µM. A captivating relationship emerged upon careful examination of the data. In essence, compound **5e** (2-Cl), containing a chlorine substituent (Table 1, entry **5e**; Scheme 1), demonstrated the highest cytotoxicity against HepG2 cell lines, followed by **5h** (2-F) (Table 1, entry **5h**; Scheme 1), and then **5l** (2-OH, 5-Br) (Table 1, entry **5l**; Scheme 1). Intriguingly, this trend was entirely reversed when evaluating its effects on HeLa, MDA-MB-231, and MCF-7 cell lines, where cytotoxicity followed the order of **5l** (2-OH, 5-Br) (Table 1, entry **5l**; Scheme 1) > **5h** (2-F) (Table 1, entry **5h**; Scheme 1) > **5e** (2-Cl) (Table 1, entry **5e**; Scheme 1). Similar altered activity was observed with compounds **5k** (3,4-di-Cl) (Table 1, entry **5k**; Scheme 1) and **5m** (3-CF_3_) (Table 1, entry **5m**; Scheme 1), aligning with HepG2 cell lines but exhibiting different patterns in the other three cell lines. Overall, the study’s results suggest that halogen atoms, particularly chlorine and fluorine, at the 2-position of phenyl rings may favor enhanced activity against the tested cancer cell lines. The position and number of halogen atoms may also exert a significant influence on activity. Additionally, the presence of a hydroxyl group at position 2 and a bromine atom at position 5 may moderately impact its activity. However, further investigations are necessary to confirm these findings and optimize the compounds’ structures for improved activity and selectivity. In summary, the study highlights the intricate relationship between structural modifications and the cytotoxicity of compounds **5a**–**m** (Scheme 1). It underscores the potential benefits of halogen substitutions, specifically chlorine and fluorine at the 2-position, while also acknowledging the influence of other factors such as the position and number of halogen atoms. These findings pave the way for future research endeavors aimed at optimizing the compounds’ structures and unlocking their full potential in terms of activity and selectivity against cancer cell lines.

#### 2.2.3. In Vitro Protein Kinase Inhibition Assays

The most active compounds, namely **5e**, **5h**, **5k**, and **5l**, were subjected to further analysis to assess their enzymatic activities against a range of kinase enzymes, including EGFR, Her2, VEGFR2, and CDK2 (Table 2). To establish the activity of these compounds, a widely recognized kinase inhibitor (sunitinib) was employed as a reference standard for all the tested kinases. In addition, other well-established inhibitors such as erlotinib for EGFR, sorafenib for VEGFR2, and staurosporine for HER2 and CDK2 were also used for comparison. Notably, compound **5k** exhibited outstanding tyrosine kinase inhibitory activities against all the tested enzymes. It displayed superior potency (IC_50_ = 79 nM) compared to sunitinib (IC_50_ = 93 nM) and was comparable to erlotinib (IC_50_ = 55 nM) against EGFR. Impressively, it demonstrated nearly identical potency (1-fold, IC_50_ = 40 nM) to the known Her2 inhibitor, staurosporine (IC_50_ = 38 nM), against the Her2 enzyme. It is worth mentioning that **5k** also exhibited a two-fold increase in potency (IC_50_ = 136 nM) when compared to sunitinib (IC_50_ = 261 nM) against VEGFR2 enzymes. These findings underscore the potential of compound **5k** as a promising candidate for further development as a kinase inhibitor.

#### 2.2.4. Cell Cycle Analysis

**Figure 2:**
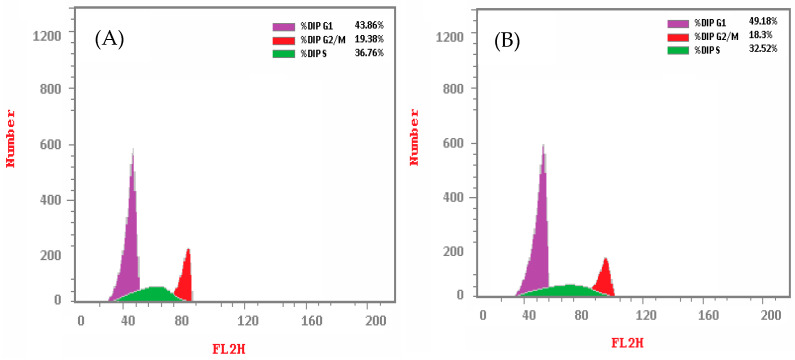
The cell cycle phases of HepG2 cells after treatment with compound **5k**: (**A**) Control HepG2 cell lines; (**B**) HepG2 cells treated with compound **5k**.

The objective of the study was to evaluate the influence of the synthesized compounds on the progression of the cell cycle in HepG2 cells. To achieve this, the cells, in a density of 2 × 10^5^/well of 6 well-plates, were exposed to **5k** at concentrations corresponding to their IC50 values for a duration of 24 h, followed by staining with propidium iodide for analysis of cell cycle phase distribution using flow cytometry. The results, which are presented in Table 3 and Figure 2, showed that the DNA content of cells treated with **5k** increased in the G1 phase and decreased in the S and G2/M phases, indicating an antiproliferative effect of the compounds. The findings suggested that treatment with the drug altered the distribution of HepG2 cells across different cell cycle phases. Specifically, the percentage of cells in the G0-G1 phase increased from 43.86% to 49.18%, while the percentage of cells in the S phase decreased from 36.76% to 32.52%, and the percentage of cells in the G2/M phase decreased from 19.38% to 18.3%.

The observed modifications in cell cycle distribution following drug treatment indicate a potential mechanism of action involving cell cycle arrest, a commonly employed therapeutic strategy for suppressing cancer cell proliferation. By impeding cells in the G0–G1 phase, drugs can effectively prevent them from proceeding to the S phase, where DNA replication takes place, and ultimately lead to cell death. Furthermore, the decrease in the proportion of cells in the S and G2/M phases suggests that the drug impacts cell cycle progression beyond the G0–G1 phase, potentially through the attenuation of DNA synthesis and cell division. The cell cycle distribution index (CDI) is a metric that quantifies the rate of cell proliferation and can be derived from the percentage of cells in each phase of the cell cycle. The CDI was calculated using the formula: CDI = (G2/M + S)/(G0 − G1), wherein G0/G1, S, and G2/M denote the percentages of cells in the respective phases of the cell cycle. The CDI values for the control and compound **5k** were determined to be 1.35 and 1.03, respectively. A decrease in CDI from the control to the drug indicates a reduced rate of cell proliferation and is suggestive of cell cycle arrest. Figure 2 shows the DNA content (%) of control HepG2 cell lines and along with compound **5k**.

#### 2.2.5. Apoptosis Analysis

##### Annexin-V/Propidium Iodide (PI) Staining Assay

**Figure 3:**
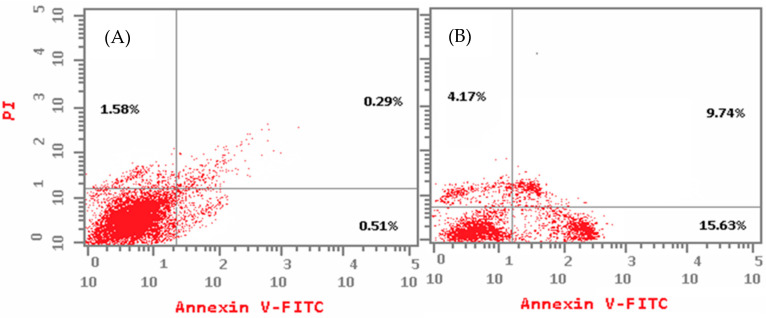
The distribution of apoptotic cells: (**A**) HepG2 cells as control; (**B**) HepG2 cells treated with compound **5k**.

To investigate the type of cell death induced by compound **5k**, flow cytometry analysis was conducted. HepG2 cells were exposed to **5k** at concentrations corresponding to their IC50 values for a duration of 24 h. Subsequently, the cells were double stained with Annexin V and propidium iodide (PI). The findings suggest that treatment with **5k** has an effect on the apoptotic and necrotic cell distribution of HepG2 cells. Apoptosis is a process of programmed cell death that occurs naturally in the body, while necrosis is a type of cell death that occurs due to damage or injury to the cell. The data show that the percentage of apoptotic cells in the total cell population was higher when treated with **5k** compared to the control group. In the early stage of the experiment, the percentage of apoptotic cells was 0.51% in the control group and 15.63% in the **5k** treated group. In the late stage, the percentage of apoptotic cells was 0.29% in the control group and 9.74% in the **5k** treated group. This suggests that treatment with **5k** may induce apoptosis in HepG2 cells, particularly in the early stages (Figure 3 and Table 4).

In contrast, the percentage of necrotic cells was also higher in the **5k** treated group compared to the control group. The percentage of necrotic cells was 1.58% in the control group and 4.17% in the **5k** treated group. This suggests that treatment with **5k** may also induce necrosis in HepG2 cells. Taken together, these findings suggest that treatment with compound **5k** may have an effect on cell death pathways in HepG2 cells, leading to increased apoptosis and necrosis. Further studies are needed to determine the underlying mechanisms of this effect and to assess the potential therapeutic applications of compound **5k** in the treatment of diseases involving abnormal cell death. Figure 3 shows the apoptosis (%) of control HepG2 cell lines and along with compound **5k**.

##### Determination of Apoptotic Protein Levels

To validate the mechanism of cell death and the induction of apoptosis caused by compound **5k**, the expression levels of some apoptotic proteins were investigated. The results showed that treatment with **5k** has an effect on the levels of apoptotic proteins in HepG2 cells (Table 5). Specifically, the levels of caspase-3 (6.9 folds higher than the control) and Bax (2.6 folds higher than the control) were higher in the group treated with **5k** compared to the control group. Caspase-3 is a key enzyme involved in the execution of apoptosis, while Bax is a pro-apoptotic protein that promotes cell death. The higher levels of caspase-3 and Bax in the **5k** treated group suggest that treatment with **5k** may induce apoptosis in HepG2 cells. In contrast, the level of the anti-apoptotic protein Bcl-2 was lower in the **5k** treated group compared to the control group (two-fold lower than the control). Bcl-2 is a protein that inhibits apoptosis, so the lower levels of Bcl-2 in the **5k** treated group further support the notion that treatment with **5k** may induce apoptosis in HepG2 cells. The levels of caspase-3, Bax, and Bcl-2 were also measured for a known drug called Saurosporine and we found that synthesized compound **5k** is comparable with Saurosporine. Overall, the results suggest that treatment with compound **5k** may induce apoptosis in HepG2 cells by upregulating the pro-apoptotic protein Bax and the execution enzyme caspase-3 while downregulating the anti-apoptotic protein Bcl-2. These findings may have implications for the development of novel therapies for diseases involving abnormal cell death, including cancer. Further studies are warranted in order to elucidate the underlying mechanisms of action of compound **5k** and to assess its potential therapeutic applications.

### 2.3. In Silico Studies

#### 2.3.1. Molecular Docking

In order to investigate potential binding interactions between compound **5k** and protein kinase enzymes (specifically EGFR, VEGFR2, HER2, and CDK2), molecular docking studies were conducted. The active sites of EGFR (PDB: 4hjo), VEGFR2 (PDB: 4asd), HER2 (PDB: 3rcd), and CDK2 (PDB: 3ti1) were utilized for docking, employing cocrystal ligands erlotinib, sorafenib, TAK-285 (a potent HER2 inhibitor featuring Pyrrolo[2,3-*d*]pyrimidine as a pharmacophore) [32], and sunitinib as reference standards, respectively. Table 6 shows the binding energies of compound **5k** and the reference standards against the selected protein kinases generated by AutoDock Vina.

**Figure 4:**
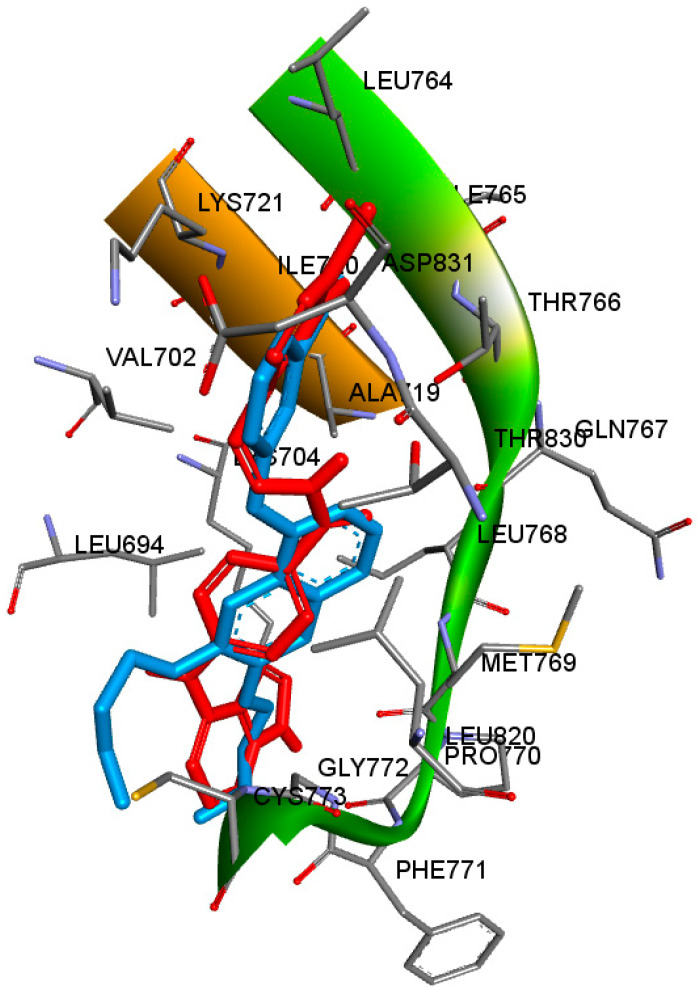
Superimposition of compound **5k** (red) and erlotinib (blue) in the active site of EGFR.

At the beginning, compound **5k** and erlotinib were docked into the active site of EGFR enzyme. Orientation of **5k** and erlotinib displayed the superimposition of the two compounds inside the active pocket (Figure 4).

**Figure 5:**
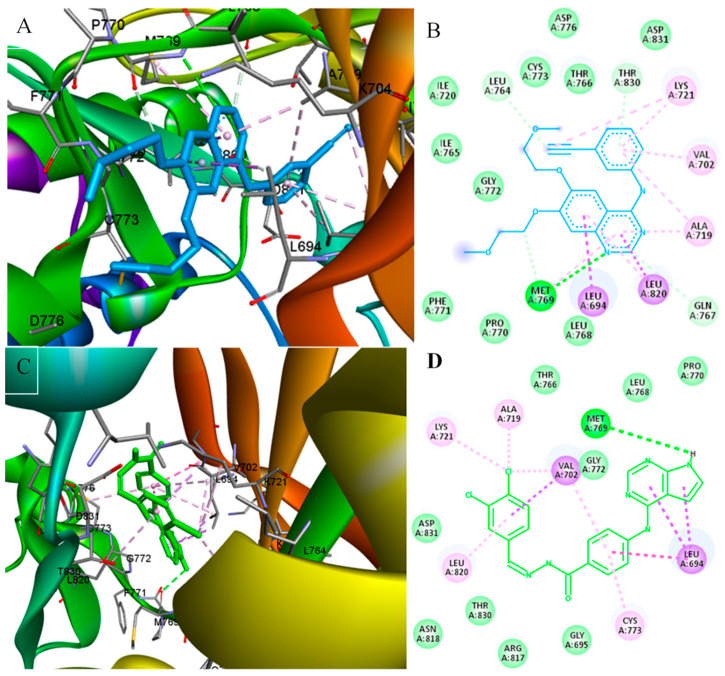
The interactions between erlotinib**/5k** and the amino acid residues within the active site of EGFR: (**A**) 3D interactions of erlotinib; (**B**) 2D interactions of erlotinib; (**C**) 3D interactions of compound **5k**; (**D**) 2D interactions of compound **5k**. Green dotted lines indicate hydrogen bonds.

Furthermore, compound **5k** and erlotinib were found to interact with similar amino acid residues. The nitrogen of the pyrrole ring in compound **5k** made a hydrogen bond with Met769, with nitrogen number 1 of the quinazoline ring in erlotinib; this interacted with Met769 by hydrogen bonding. Both compounds were stabilized by hydrophobic interactions with Leu694, Val702, Ala719, Lys721, and Leu773 (Figure 5).

**Figure 6:**
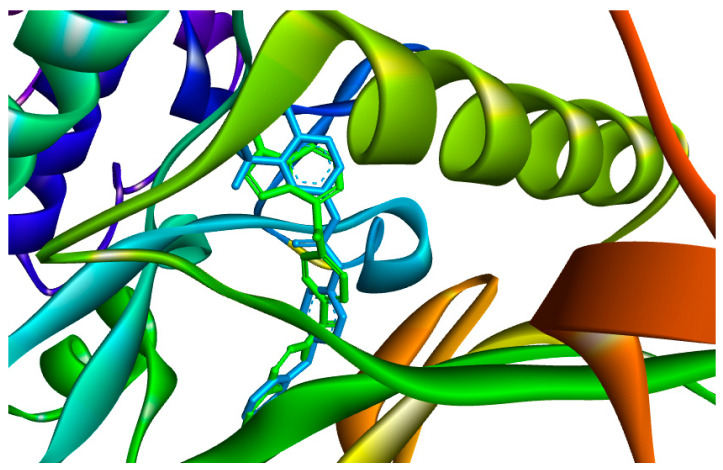
Superimposition of compound **5k** (green) and sorafenib (blue) in the active site of VEGFR2.

Then, compound **5k** and sorafenib were docked into the active site of VEGFR2. Compound **5k** and sorafenib are superimposed (Figure 6); they were stabilized by interacting with almost different amino acids residues.

**Figure 7:**
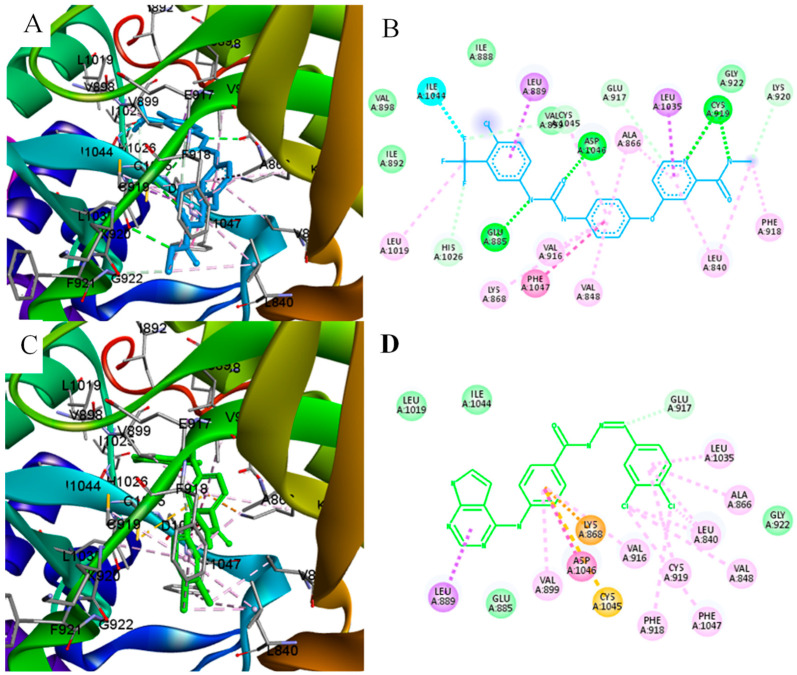
The interactions between sorafenib/**5k** and the amino acid residues within the active site of VEGFR2: (**A**) 3D interactions of sorafenib; (**B**) 2D interactions of sorafenib; (**C**) 3D interactions of compound **5k**; (**D**) 2D interactions of compound **5k**. Green dotted lines indicate hydrogen bonds.

Unlike sorafenib, compound **5k** did not make any hydrogen bonds, but it was stabilized by multiple alkyl and Pi alkyl interactions of the chloro substituents (Figure 7).

**Figure 8:**
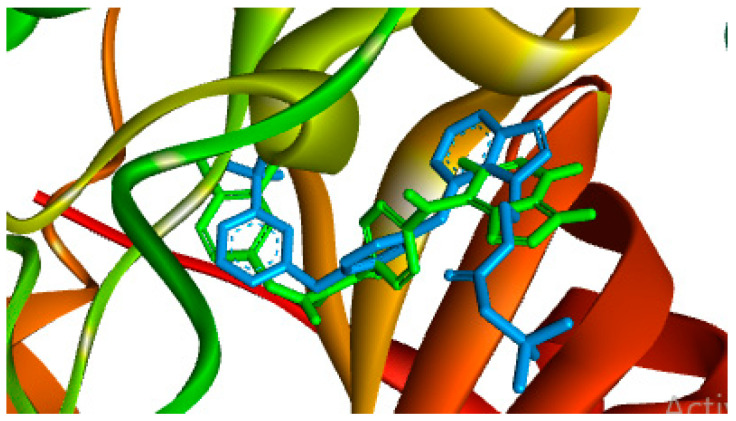
Superimposition of compound **5k** (green) and TAK-285 (blue) in the active site of Her2.

Afterwards, docking of compound **5k** and TAK-285 were studied in the active site of Her2 receptor. The alignment of both compounds is demonstrated in Figure 8.

**Figure 9:**
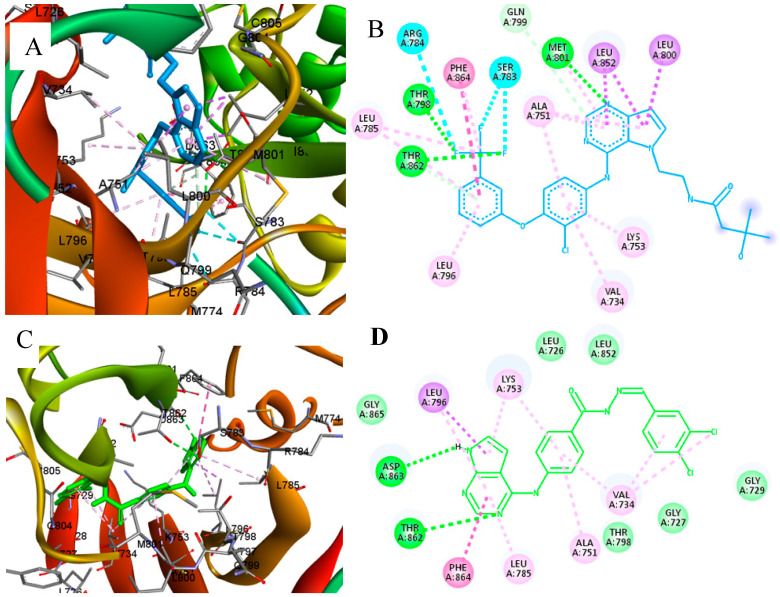
The interactions between TAK-285**/5k** and the amino acid residues within the active site of HER2: (**A**) 3D interactions of TAK-285; (**B**) 2D interactions of TAK-285; (**C**) 3D interactions of compound **5k**; (**D**) 2D interactions of compound **5k**. Green dotted lines indicate hydrogen bonds.

TAK-285 was stabilized by three hydrogen bonds (Met801, Thr798, and Thr862) while compound **5k** made only two hydrogen bonds (ASP863 and Thr862). Both compounds interacted by hydrophobic interactions with Ala751, Phe864, Leu785, Leu796, Val734, and Lys753 (Figure 9).

**Figure 10:**
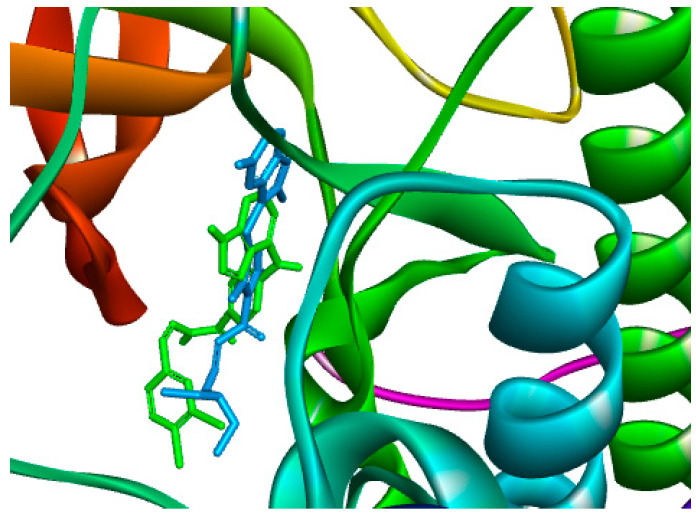
Superimposition of compound **5k** (green) and sunitinib (blue) in the active site of CDK2.

Lastly, compound **5k** and sunitinib were docked into the active site of CDK2. The superimposed pose of **5k** was found to be stabilized with two hydrogen bonds (Leu80 and Glu8), while sunitinib made only one hydrogen bond (Leu83) (Figure 10).

**Figure 11:**
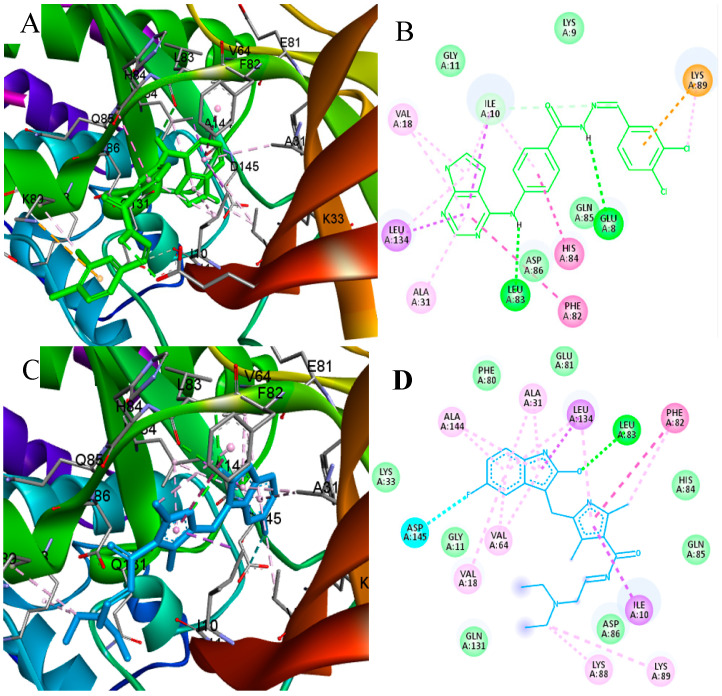
The interactions between sunitinib**/5k** and the amino acid residues within the active site of CDK2: (**A**) 3D interactions of compound **5k**; (**B**) 2D interactions of compound **5k**. (**C**) 3D interactions of sunitinib; (**D**) 2D interactions of sunitinib; Green dotted lines indicate hydrogen bonds.

On the other hand, sunitinib interacted with more amino acid residues by hydrophobic interactions when compared to compound **5k** (Figure 11).

The order of magnitude difference in IC_50_ values between compound **5k** and compounds **5e**, **5h**, and **5l** indicates that **5k** has superior binding affinity to the target protein’s active site, suggesting its potential as a potent kinase inhibitor for targeted therapies. However, the lack of correlation between compound efficacy and binding affinity in cytotoxicity assays raises intriguing questions. It is possible that variations in cellular uptake, metabolism, or off-target effects contribute to the observed differences, allowing compounds **5e**, **5h**, and **5l** to exhibit comparable or even better cytotoxic effects despite weaker binding affinity. To fully understand this phenomenon, further analysis of the specific characteristics of the kinase pathway, cellular context, and downstream effects of kinase inhibition is warranted.

#### 2.3.2. In Silico ADMET Studies

The pkCSM protocol, utilizing graph-based signatures, was employed to evaluate the in-silico profile of the most active compounds, as well as sorafenib as a reference standard, encompassing predicted physicochemical properties, absorption, distribution, metabolism, elimination, and toxicity (ADMET) [33] (Table 7). Notably, all synthesized compounds adhere to Lipinski’s rule of five, indicating their favorable drug-like characteristics. Analysis of the results presented in Table 6 signifies the potential for good intestinal absorption for these compounds, suggesting a likelihood of favorable oral bioavailability. Moreover, the synthesized compounds exhibit a low permeability through the blood-brain barrier, thereby indicating a reduced probability of adverse effects on the central nervous system, albeit mild effects might still be observed. Additionally, it is anticipated that the major biotransforming enzymes, namely CYP2D6 and CYP3A4, will play a significant role in the metabolism of these compounds. On the other hand, the projected toxicity profile demonstrates a high maximum tolerated dose, along with low rates of oral acute and chronic toxicity for the synthesized compounds. Notably, all synthesized compounds, including sorafenib, are predicted to induce hepatotoxicity while not displaying skin sensitivity. These comprehensive in-silico assessments provide valuable insights into the pharmacological properties and safety aspects of the synthesized compounds, supporting their potential development and exploration as kinase inhibitors.

## 3. Materials and Methods

### 3.1. General

The experiments were conducted using commercially available reagents and solvents without any additional purification. Melting points were measured using a Barnstead electrothermal digital melting point apparatus (model IA9100, BIBBY scientific limited, Staffordshire, UK). IR spectra were recorded using a Jasco FT/IR-6600 spectrometer (Tokyo, Japan). NMR spectra were obtained using a Bruker 700 MHz NMR spectrometry instrument (Zurich, Switzerland). Electrospray ionization (ESI) mass spectrometry (MS) experiments were carried out using an Agilent 6320 ion trap mass spectrometer equipped with an ESI ion source (Agilent Technologies, Palo Alto, CA, USA). High-resolution mass spectrometry was performed using an HRMS JMS 700 JEOL mass spectrometry instrument (Tokyo, Japan). The compounds were judged to be pure based on NMR spectroscopic studies. All spectra for ^1^H-NMR, ^13^CNMR and Mass spectra are found in the Supplementary Materials.

### 3.2. Chemistry

#### 3.2.1. Ethyl-4-((7H-Pyrrolo[2,3-d]pyrimidin-4-yl)amino)benzoate (**3**)

Compound **3** was synthesized according to our previously reported method [31]. A solution of 4-chloro-*7H*-Pyrrolo[2,3-*d*]pyrimidine (**1**, 3.072 g, 0.02 mol) and of ethyl-4-aminobenzoate (**2**, 3.304 g, 0.02 mol) in 100 mL of absolute ethanol was heated under reflux for 7 h. The progress of the reaction was monitored using thin-layer chromatography (TLC). Once the reaction was complete, the mixture was cooled to room temperature, and the resulting precipitate was filtered under vacuum. The obtained solid was washed with cold ethanol, dried overnight, and yielded compound **3** as a white solid. (4.74 g, 16.8 mmol, 84%). Mp. 242 °C (Lit Mp. 242 °C) [31].

#### 3.2.2. 4-((7H-Pyrrolo[2,3-d]pyrimidin-4-yl)amino)benzohydrazide (**4**)

Compound **4** was synthesized according to our previously reported method [31]. A mixture of ethyl-4-((7*H*-Pyrrolo[2,3-*d*]pyrimidin-4-yl)amino)benzoate (**3**, 2.0 g, 7.06 mmol) in hydrazine monohydrate (25 mL, 98%) was heated under reflux for 6 h. After cooling the reaction mixture to room temperature, the resulting precipitate was filtered under vacuum. The obtained solid was washed with cold water, dried overnight, and yielded compound **4** as a white solid. (1.345 g, 5.01 mmol, 71%). Mp. 300 °C (Lit Mp. 300 °C) [31].

#### 3.2.3. General Synthetic Method for the Synthesis of **5a**–**m**

Compounds **5a**–**m** were synthesized according to our previously reported method [31]. In brief, compound **4** (100 mg, 0.373 mmol) and substituted-aldehyde (1.2 equiv.) was placed in a round bottom flask (RBF) with 10 mL of absolute ethanol. Then, 3–4 drops of (cat.) glacial acetic acid were added to the reaction mixture, which was then refluxed overnight. The resulting precipitate was filtered, washed with ethanol, and dried under vacuum.

##### (*E*)-4-((7*H*-Pyrrolo[2,3-*d*]pyrimidin-4-yl)amino)-*N*’-benzylidenebenzohydrazide (**5a**) [31]

White solid (120 mg, 0.343 mmol, 92%). Mp. 294 °C (Lit Mp. 294 °C) [31].

##### (*E*)-4-((7*H*-Pyrrolo[2,3-*d*]pyrimidin-4-yl)amino)-*N*’-(2-bromobenzylidene)benzohydrazide (**5b**)

White solid (157 mg, 0.362 mmol, 97%). Mp. 294 °C. FT-IR (KBr): ν (cm^−1^) = 3452, 3424, 3193, 1638, 1611, 1574, 1521, 1465, 1357, 1293, 1184, 1023, 898, 759, 719 and 607 cm^−1^. ^1^H-NMR (700 MHz, DMSO-*d_6_*) δ 12.03 (s, 1H), 11.87 (s, 1H), 9.64 (s, 1H), 8.84 (s, 1H), 8.38 (s, 1H), 8.11 (d, *J* = 8.4 Hz, 2H), 8.03 (d, *J* = 7.8 Hz, 1H), 7.96 (d, *J* = 8.3 Hz, 2H), 7.72 (d, *J* = 8.0 Hz, 1H), 7.49 (t, *J* = 7.5 Hz, 1H), 7.38 (t, *J* = 7.7 Hz, 1H), 7.31 (t, *J* = 2.9 Hz, 1H) and 6.87 (dd, *J* = 3.6, 1.9 Hz, 1H) ppm. ^13^C-NMR (176 MHz, DMSO-*d_6_*) δ 163.24, 153.50, 151.57, 151.07, 145.73, 144.52, 133.73, 133.64, 132.08, 128.97, 128.59 (2C), 127.70, 126.16, 123.94, 123.28, 119.31 (2C), 104.66 and 99.20 ppm. Mass (ESI): *m/z* 435 [^79^(Br)M + H]^+^, 437 [^81^(Br)M + H]^+^; 457 [^79^(Br)M + Na]^+^, 473 [^79^(Br)M + K]^+^, 475 [^81^(Br)M + K]^+^; HRMS (ESI): Calc. for C_20_H_15_BrN_6_O [M]^+^ 434.0491, found 434.6068.

##### (*E*)-4-((7*H*-Pyrrolo[2,3-*d*]pyrimidin-4-yl)amino)-*N*’-(3-bromobenzylidene)benzohydrazide (**5c**)

White solid (151 mg, 0.347 mmol, 93%). Mp. 296 °C. FT-IR (KBr): ν (cm^−1^) = 3417, 3214, 2988, 2850, 2711, 1639, 1613, 1585, 1572, 1518, 1471, 1421, 1348, 1279, 1240, 1190, 1144, 1070, 951, 913, 895, 790, 759, 721, 683 and 521 cm^−1^. ^1^H-NMR (700 MHz, DMSO-*d_6_*) δ 11.90 (s, 1H), 11.87 (s, 1H), 9.63 (s, 1H), 8.44 (s, 1H), 8.38 (s, 1H), 8.11 (d, *J* = 8.8 Hz, 2H), 7.94 (d, *J* = 8.1 Hz, 3H), 7.74 (d, *J* = 7.6 Hz, 1H), 7.64 (d, *J* = 7.4 Hz, 1H), 7.44 (t, *J* = 7.8 Hz, 1H), 7.31 (dd, *J* = 3.4, 2.2 Hz, 1H) and 6.87 (dd, *J* = 3.5, 1.8 Hz, 1H) ppm. ^13^C-NMR (176 MHz, DMSO-*d_6_*) δ 163.31, 153.51, 151.56, 151.07, 145.68, 144.48, 137.48, 132.90, 131.51, 129.48 (2C), 128.95, 126.64, 126.24, 123.27, 122.66, 119.32 (2C), 104.65 and 99.20 ppm. Mass (ESI): *m/z* 435 [^79^(Br)M + H]^+^, 437 [^81^(Br)M + H]^+^; 457 [^79^(Br)M + Na]^+^, 459 [^81^(Br)M + Na]^+^, 473 [^79^(Br)M + K]^+^, 475 [^81^(Br)M + K]^+^.

##### (*E*)-4-((7*H*-Pyrrolo[2,3-*d*]pyrimidin-4-yl)amino)-*N*’-(4-bromobenzylidene)benzohydrazide (**5d**)

White solid (156 mg, 0.358 mmol, 96%). Mp. 321 °C. FT-IR (KBr): ν (cm^−1^) = 3440, 3396, 3212, 3098, 3048, 2992, 2850, 2708, 1653, 1618, 1588, 1575, 1527, 1477, 1424, 1357, 1313, 1298, 1245, 1193, 1667, 1009, 897, 828, 719, 980 and 514 cm^−1^. ^1^H-NMR (700 MHz, DMSO-*d_6_*) δ 11.87 (s, 1H), 11.83 (s, 1H), 9.63 (s, 1H), 8.45 (s, 1H), 8.37 (s, 1H), 8.10 (d, *J* = 8.8 Hz, 2H), 7.94 (d, *J* = 8.3 Hz, 2H), 7.73–7.65 (m, 4H), 7.31 (dd, *J* = 3.4, 2.3 Hz, 1H) and 6.87 (dd, *J* = 3.5, 1.9 Hz, 1H) ppm. ^13^C-NMR (176 MHz, DMSO-*d_6_*) δ 163.23, 153.51, 151.56, 151.07, 146.26, 144.42, 134.28, 132.33 (4C), 129.34 (2C), 128.90, 126.33, 123.59, 123.26, 119.33 (2C), 104.64 and 99.20 ppm. Mass (ESI): *m/z* 435 [^79^(Br)M + H]^+^, 437 [^81^(Br)M + H]^+^; 457 [^79^(Br)M + Na]^+^, 459 [^81^(Br)M + Na]^+^, 473 [^79^(Br)M + K]^+^, 475 [^81^(Br)M + K]^+^.

##### (*E*)-4-((7*H*-Pyrrolo[2,3-*d*]pyrimidin-4-yl)amino)-*N*’-(2-chlorobenzylidene)benzohydrazide (**5e**)

White solid (143 mg, 0.365 mmol, 98%). Mp. 298 °C. FT-IR (KBr): ν (cm^−1^) = 3301, 3214, 3110, 3066, 3015, 2923, 2850, 1655, 1617, 1569, 1464, 1360, 1281, 1265, 1246, 1074, 855, 757, 727 cm^−1^. ^1^H-NMR (700 MHz, DMSO-*d_6_*) δ 12.00 (s, 1H), 11.87 (s, 1H), 9.64 (s, 1H), 8.89 (s, 1H), 8.38 (s, 1H), 8.11 (d, *J* = 8.5 Hz, 2H), 8.06 (s, 1H), 7.96 (d, *J* = 8.3 Hz, 2H), 7.59–7.52 (m, 1H), 7.46 (dd, *J* = 6.7, 3.3 Hz, 2H), 7.31 (dd, *J* = 3.4, 2.3 Hz, 1H) and 6.87 (dd, *J* = 3.4, 1.9 Hz, 1H) ppm. ^13^C-NMR (176 MHz, DMSO-*d_6_*) δ 163.23, 153.50, 151.56, 151.07, 144.52, 143.39, 133.56, 132.22, 131.83, 130.41 (2C), 128.96, 128.11 (2C), 127.32, 126.15, 123.28, 119.32 (2C), 104.66 and 99.20 ppm. Mass (ESI): *m/z* 391 [^35^(Cl)M + H]^+^, 393 [^37^(Cl)M + H]^+^, 413 [^35^(Cl)M + Na]^+^, 415 [^37^(Cl)M + Na]^+^; HRMS (ESI): Calc. for C_20_H_15_ClN_6_O [M]^+^ 390.0996, found 389.9511.

##### (*E*)-4-((7*H*-Pyrrolo[2,3-*d*]pyrimidin-4-yl)amino)-*N*’-(3-chlorobenzylidene)benzohydrazide (**5f**)

White solid (138 mg, 0.354 mmol, 95%). Mp. 281 °C. FT-IR (KBr): ν (cm^−1^) = 3520, 3448, 3215, 3094, 2988, 2850, 1626, 1612, 1575, 1466, 1430, 1354, 1292, 1250, 1187, 1128, 1059, 897, 827, 783, 734, 685 and 520 cm^−1^. ^1^H-NMR (700 MHz, DMSO-*d_6_*) δ 11.87 (s, 1H), 11.85 (s, 1H), 9.61 (s, 1H), 8.43 (s, 1H), 8.36 (s, 1H), 8.09 (dd, 2H), 7.92 (d, *J* = 8.3 Hz, 2H), 7.78 (s, 1H), 7.68 (s, 1H), 7.49 (d, *J* = 4.5 Hz, 2H), 7.29 (dd, *J* = 3.4, 2.3 Hz, 1H) and 6.85 (dd, *J* = 3.5, 1.8 Hz, 1H) ppm. ^13^C-NMR (176 MHz, DMSO-*d_6_*) δ 163.31, 153.51, 151.57, 151.07, 145.79, 144.47, 137.25, 134.13, 131.24 (2C), 130.02, 128.95, 126.64, 126.22, 123.27, 119.32 (2C), 104.65 and 99.20 ppm. Mass (ESI): *m/z* 391 [^35^(Cl)M + H]^+^, 393 [^37^(Cl)M + H]^+^; HRMS (ESI): Calc. for C_20_H_15_ClN_6_O [M]^+^ 390.0996, found 389.9585.

##### (*E*)-4-((7*H*-Pyrrolo[2,3-*d*]pyrimidin-4-yl)amino)-*N*’-(4-chlorobenzylidene)benzohydrazide (**5g**)

White solid (134 mg, 0.343 mmol, 92%). Mp. 314 °C. FT-IR (KBr): ν (cm^−1^) = 3418, 3350, 3086, 2964, 2848, 1648, 1609, 1573, 1474, 1419, 1349, 1281, 1236, 1185, 1046, 899, 824, 721, 633 and 604 cm^−1^. ^1^H-NMR (700 MHz, DMSO-*d_6_*) δ 11.87 (s, 1H), 11.83 (s, 1H), 9.63 (s, 1H), 8.47 (s, 1H), 8.38 (s, 1H), 8.11 (d, *J* = 8.4 Hz, 2H), 7.94 (d, *J* = 8.3 Hz, 2H), 7.77 (d, *J* = 8.1 Hz, 2H), 7.54 (d, *J* = 8.0 Hz, 2H), 7.31 (dd, *J* = 8.1 Hz, 1H) and 6.87 (dd, *J* = 3.3, 1.7 Hz, 1H) ppm. ^13^C-NMR (176 MHz, DMSO-*d6*) δ 163.24, 153.52, 151.57, 151.07, 146.19, 144.43, 134.81, 133.94, 129.42 (4C), 129.10, 128.91 (2C), 126.35, 123.26, 119.34 (2C), 104.65 and 99.20 ppm. Mass (ESI): *m/z* 391 [M + H]^+^, 413 [M + Na]^+^; HRMS (ESI): Calc. for C_20_H_15_ClN_6_O [M]^+^ 390.0996, found 389.9400.

##### (*E*)-4-((7*H*-Pyrrolo[2,3-*d*]pyrimidin-4-yl)amino)-*N*’-(2-fluorobenzylidene)benzohydrazide (**5h**)

White solid (130 mg, 0.347 mmol, 93%). Mp. 307 °C. FT-IR (KBr): ν (cm^−1^) = 3419, 3365, 3094, 2966, 2849, 1648, 1609, 1572, 1475, 1420, 1350, 1282, 1238, 1188, 1047, 900, 826, 719, 632 and 506 cm^−1^. ^1^H-NMR (700 MHz, DMSO-*d_6_*) δ 11.89 (s, 1H), 11.87 (s, 1H), 9.64 (s, 1H), 8.72 (s, 1H), 8.38 (s, 1H), 8.11 (d, *J* = 8.5 Hz, 2H), 7.98 (s, 1H), 7.95 (d, *J* = 8.4 Hz, 2H), 7.51 (q, *J* = 7.1 Hz, 1H), 7.33 (d, *J* = 7.8 Hz, 1H), 7.32 (d, *J* = 3.1 Hz, 1H), 7.31 (d, *J* = 2.2 Hz, 1H) and 6.87 (dd, *J* = 3.5, 1.7 Hz, 1H) ppm. ^13^C-NMR (176 MHz, DMSO-*d_6_*) δ 162.62 (^1^*J*_F_ = 237 Hz), 160.51, 153.51, 151.52, 151.06, 144.48, 140.26, 132.38, 128.92 (2C), 126.79 (^3^*J*_F_ = 2.6 Hz), 126.26, 125.44 (^3^*J*_F_ = 3.2 Hz), 123.30, 122.45 (^2^*J*_F_ = 9.8 Hz), 119.37 (2C), 116.48 (^2^*J*_F_ = 18.0 Hz), 104.65 and 99.21 ppm. Mass (ESI): *m/z* 375 [M + H]^+^, 397 [M + Na]^+^, 413 [M + K]^+^; HRMS (ESI): Calc. for C_20_H_15_FN_6_O [M]^+^ 374.1291, found 374.0985.

##### (*E*)-4-((*7H*-Pyrrolo[2,3-*d*]pyrimidin-4-yl)amino)-*N*’-(3-fluorobenzylidene)benzohydrazide (**5i**)

White solid (134 mg, 0.358 mmol, 96%). Mp. 295 °C. FT-IR (KBr): ν (cm^−1^) = 3432, 3227, 3119, 3063, 2924, 1653, 1610, 1576, 1474, 1293, 1235, 1194, 1065, 958, 889, 785, 728, 688 and 521 cm^−1^. ^1^H-NMR (700 MHz, DMSO-*d_6_*) δ 11.87 (s, 1H), 11.83 (s, 1H), 9.64 (s, 1H), 8.44 (s, 1H), 8.34 (s, 1H), 8.08 (d, *J* = 8.8 Hz, 2H), 7.91 (d, *J* = 8.3 Hz, 2H), 7.56 (t, *J* = 10.7 Hz, 2H), 7.50 (q, *J* = 7.4 Hz, 1H), 7.28 (dd, *J* = 3.4, 2.3 Hz, 1H), 7.26 (td, *J* = 8.6, 2.6 Hz, 1H) and 6.84 (dd, *J* = 3.5, 1.9 Hz, 1H) ppm. ^13^C-NMR (176 MHz, DMSO-*d_6_*) δ 163.42, 162.90 (d, ^1^*J*_CF_ = 244 Hz), 153.51, 151.50, 151.05, 146.26, 144.45, 137.50 (d, ^3^*J*_CF_ = 8 Hz), 131.42 (d, ^3^*J*_CF_ = 8 Hz), 128.93 (2C), 126.20, 123.94, 123.30, 119.38 (2C), 117.19 (d, ^2^*J*_CF_ = 21 Hz), 113.44 (d, ^2^*J*_CF_ = 21 Hz), 104.64 and 99.21 ppm. Mass (ESI): *m/z* 375 [M + H]^+^, 397 [M + Na]^+^; HRMS (ESI): Calc. for C_20_H_15_FN_6_O [M]^+^ 374.1291, found 374.2093.

##### (*E*)-4-((7*H*-Pyrrolo[2,3-*d*]pyrimidin-4-yl)amino)-*N*’-(4-fluorobenzylidene)benzohydrazide (**5j**)

White solid (134 mg, 0.358 mmol, 96%). Mp. 309 °C. FT-IR (KBr): ν (cm^−1^) = 3430, 3232, 1651, 1606, 1576, 1504, 1473, 1287, 1236, 1218, 1191, 1059, 963, 892, 840, 729, 676 and 512 cm^−1^. ^1^H-NMR (700 MHz, DMSO-*d_6_*) δ 11.85 (s, 1H), 11.75 (s, 1H), 9.60 (s, 1H), 8.46 (s, 1H), 8.36 (s, 1H), 8.09 (d, *J* = 8.8 Hz, 2H), 7.92 (d, *J* = 8.3 Hz, 2H), 7.78 (dd, *J* = 7.2 Hz, 2H), 7.34–7.26 (m, 3H) and 6.85 (dd, *J* = 3.5, 1.7 Hz, 1H) ppm. ^13^C-NMR (176 MHz, DMSO-*d6*) δ ^13^C NMR (176 MHz, DMSO) δ 163.51 (d, ^1^*J*_CF_ = 248 Hz), 163.21, 153.52, 151.56, 151.08, 146.41, 144.37, 131.59 (d, ^4^*J*_CF_ = 2.9 Hz, 2C), 129.62 (d, ^3^*J*_CF_ = 8.6 Hz), 128.87 (2C), 126.44, 123.25, 119.34 (2C), 116.38 (d, ^2^*J*_CF_ = 21.8 Hz, 2C), 104.64 and 99.20 ppm. Mass (ESI): *m/z* 375 [M + H]^+^; HRMS (ESI): Calc. for C_20_H_16_FN_6_O [M + H]^+^ 375.1364, found 375.5109.

##### (*E*)-4-((7*H*-Pyrrolo[2,3-*d*]pyrimidin-4-yl)amino)-*N*’-(3,4-dichlorobenzylidene)benzohydrazide (**5k**)

White solid (147 mg, 0.347 mmol, 93%). Mp. 294 °C. FT-IR (KBr): ν (cm^−1^) = 3349, 3299, 3202, 3111, 2967, 1629, 1606, 1557, 1503, 1460, 1405, 1286, 1238, 1190, 1121, 1080, 891, 751, 598 and 516 cm^−1^. ^1^H-NMR (700 MHz, DMSO-*d_6_*) δ 11.96 (s, 1H), 11.87 (s, 1H), 9.64 (s, 1H), 8.44 (s, 1H), 8.38 (s, 1H), 8.12 (d, *J* = 8.8 Hz, 2H), 8.01–7.89 (m, 3H), 7.74 (d, *J* = 5.4 Hz, 2H), 7.31 (dd, *J* = 3.4, 2.4 Hz, 1H) and 6.87 (dd, *J* = 3.5, 1.9 Hz, 1H) ppm. ^13^C-NMR (176 MHz, DMSO-*d_6_*) δ 163.36, 153.50, 151.57, 151.06, 144.76, 144.53, 135.85, 132.53, 132.19, 131.57, 128.98 (2C), 128.86, 127.30, 126.15, 123.27, 119.32 (2C), 104.67 and 99.20 ppm. Mass (ESI): *m/z* 425 [M (^35^(Cl) + H]^+^, 427 [M (^37^(Cl) + H]^+^, 447 [M + Na]^+^; HRMS (ESI): Calc. for C_20_H_14_Cl_2_N_6_O [M]^+^ 424.0606, found 424.4530.

##### (*E*)-4-((7*H*-Pyrrolo[2,3-*d*]pyrimidin-4-yl)amino)-*N*’-(5-bromo-2-hydroxybenzylidene)benzohydrazide (**5l**)

White solid (152 mg, 0.358 mmol, 96%). Mp. 334 °C. FT-IR (KBr): ν (cm^−1^) = 3456, 3350, 3316, 3226, 3116, 1666, 1612, 1470, 1464, 1420, 1339, 1373, 1204, 892, 738, 628 and 593 cm^−1^. ^1^H-NMR (700 MHz, DMSO-*d_6_*) δ 12.11 (s, 1H), 11.87 (s, 1H), 11.41 (s, 1H), 9.65 (s, 1H), 8.62 (s, 1H), 8.38 (s, 1H), 8.12 (d, *J* = 8.5 Hz, 2H), 7.96 (d, *J* = 8.4 Hz, 2H), 7.80 (s, 1H), 7.44 (dd, *J* = 8.7, 2.5 Hz, 1H), 7.32 (dd, *J* = 3.4, 2.3 Hz, 1H), 6.92 (d, *J* = 8.7 Hz, 1H) and 6.87 (dd, *J* = 3.5, 1.9 Hz, 1H) ppm. ^13^C-NMR (176 MHz, DMSO-*d_6_*) δ 163.02, 156.90, 153.49, 151.58, 151.06, 145.59, 144.65, 133.88, 131.03, 128.99 (2C), 125.69, 123.31, 121.85, 119.34, 119.16 (2C), 110.88, 104.68 and 99.20 ppm. Mass (ESI): *m/z* 451 [^79^(Br)M + H]^+^, 453 [^81^(Br)M + H]^+^, 473 [^79^(Br)M + Na]^+^, 475 [^81^(Br)M + Na]^+^.

##### (*E*)-4-((7*H*-Pyrrolo[2,3-*d*]pyrimidin-4-yl)amino)-*N*’-(3-(trifluoromethyl)benzylidene)benzohydrazide (**5m**)

White solid (147 mg, 0.347 mmol, 93%). Mp. 280 °C. FT-IR (KBr): ν (cm^−1^) = 3480, 3438, 3236, 3103, 2991, 2851, 1656, 1620, 1575, 1525, 1475, 1425, 1355, 1290, 1249, 1199, 1158, 1121, 1074, 895, 719 and 530 cm^−1^. ^1^H-NMR (700 MHz, DMSO-*d_6_*) δ 11.96 (s, 1H), 11.87 (s, 1H), 9.64 (s, 1H), 8.56 (s, 1H), 8.38 (s, 1H), 8.11 (d, *J* = 8.8 Hz, 2H), 8.09 (s, 1H), 8.04 (d, *J* = 7.7 Hz, 1H), 7.95 (d, *J* = 8.3 Hz, 2H), 7.81 (d, *J* = 7.7 Hz, 1H), 7.73 (t, *J* = 7.7 Hz, 1H), 7.31 (dd, *J* = 3.4, 2.3 Hz, 1H) and 6.87 (dd, *J* = 3.4, 1.9 Hz, 1H) ppm. ^13^C NMR (176 MHz, DMSO-*d_6_*) δ ^13^C-NMR (176 MHz, DMSO) δ 163.42, 153.51, 151.56, 151.06, 145.74, 144.51, 136.17, 131.50, 130.54, 130.10 (q, ^1^*J*_CF_ = 64, 32 Hz), 128.97 (2C), 126.39 (d, ^2^*J*_CF_ = 78 Hz), 125.31, 123.76, 123.36 (d, ^3^*J*_CF_ = 3.8 Hz), 123.28, 119.34 (2C), 104.66 and 99.20 ppm. Mass (ESI), *m/z* 425 [M + H]^+^. HRMS (ESI): Calc. for C_21_H_15_F_3_N_6_O [M]^+^ 424.1259 found 424.0000.

### 3.3. Biological Evaluation

#### 3.3.1. In Vitro Cytotoxicity Assay

The cytotoxicity of compounds **5a**–**m** was assessed against various cancer cell lines and one normal cell line using the described methodology [31,34]. In brief, the cells were cultured in 96-well plates with a density of 1.0 × 10^4^ cells per well, supplemented with 10% fetal bovine serum (FBS), an antibiotic cocktail, and RPMI 11,640 medium. The cells were then incubated for 48 h with 5% CO_2_ and 100% relative humidity at 37 °C. Subsequently, the cells were exposed to varying concentrations of the synthesized compounds and sunitinib for a duration of 24 h. Following treatment, the addition of 20 µL of MTT solution was added to each well and incubation was continued for another 4 h, after which dimethyl sulfoxide (100 µL) was used to dissolve the resulting insoluble formazan. The absorbance was measured at 570 nm using a BioTek EXL 800 plate reader (Agilent Technologies, CA, USA). By comparing the absorbance of treated samples to untreated samples, the relative cell viability percentage was calculated as (A570 of treated samples/A570 of untreated sample) × 100. The IC_50_ values were determined based on triplicate measurements.

#### 3.3.2. In Vitro Enzyme Assays

In accordance with the documented procedures [31,34], fifteen derivatives of the synthesized compounds were evaluated for their inhibitory effects on EGFR, VEGFR-2, Her2, and CDK2 using specific human ELISA kits (Enzyme-Linked Immunosorbent Assay) designed for each respective kinase enzyme. Initially, various concentrations of the synthesized compounds were combined with specific antibodies for each enzyme in individual wells of a 96-well plate, followed by incubation at room temperature for 2.5 h. Subsequently, the plate was washed, and a biotin antibody (100 µL), prepared in-house, was added and allowed to incubate at ambient temperature for 1 h. After another washing step, a solution of streptavidin (100 µL) was introduced to each well and left for 45 min at room temperature. Following a third round of washing, a TMB Substrate (100 µL) reagent was applied to all wells and incubated for 30 min at room temperature. Finally, the reaction was terminated by adding 50 µL of a stop solution, and the absorbance was directly measured at 450 nm. A standard curve was constructed, illustrating the relationship between concentration (*X*-axis) and absorbance (*Y*-axis).

#### 3.3.3. Analysis of Cell Cycle Progression

To investigate the impact of the synthesized compound **5k** on cell cycle progression, the ab139418 Propidium Iodide flow cytometry kit/BD was utilized [31]. Initially, HepG2 cells were seeded in triplicate at a density of 2 × 10^5^ cells per well in 6-well plates and incubated for 24 h. Subsequently, the cells were treated with the selected compounds at concentrations corresponding to their respective IC_50_ values and incubated for an additional 24 h. Following treatment, the HepG2 cells were fixed with 70% ethanol and incubated for 12 h at 4 °C. Subsequently, the wells were washed with cold PBS, treated with RNase A (100 µL) at 37 °C for 30 min, and stained with Propidium Iodide (400 µL) in a dark environment at room temperature for another 30 min. The labeled cells were then analyzed using the Epics XLMCL™ flow cytometer equipment (Beckman Coulter, Apeldoorn, the Netherlands). Finally, the experimental results were analyzed using Turku Centre for Biotechnology software version 2.5.1 (Turku, Finland).

#### 3.3.4. Annexin-V/Propidium Iodide (PI) Double Staining Assay

To investigate the apoptotic effects of the synthesized compound **5k**, HepG2 cells were cultured in triplicate at a density of 2.0 × 10^5^ cells per well. Subsequently, the cells were treated with the selected compounds at their respective IC_50_ values determined by the MTT assay [34,35]. After 24 h, the cells were detached using trypsin, collected, and then centrifuged. Following two washes with PBS, the cells were resuspended in 0.1 mL of binding buffer. Dual staining was performed by adding Annexin V-FITC (5 µL) and propidium iodide (5 µL) to the cell suspension, which was then incubated in the dark for 15 min at room temperature. Flow cytometry analysis was performed using the Epics XL-MCL™ flow cytometry equipment (Beckman Coulter, Apeldoorn, The Netherlands) with an excitation wavelength of 488 nm and an emission wavelength of 530 nm. Finally, the experimental results were analyzed using flowing “Turku Centre for Biotechnology” software, version 2.5.1 (Turku, Finland).

#### 3.3.5. Apoptotic Protein Levels Assay

The ELISA technique was employed to assess the impact of compound **5k** on the alteration of protein levels associated with caspase-3, BAX, and Bcl-2. Specifically, the following ELISA kits were utilized: KHO1091 (InvitrogenTM, Grand Island, NY, USA) for caspase-3, EIA-4860 and EIA-4487 for BAX, (DRU International INC., Mountainside, NJ, USA), and 99-0042 (InvitrogenTM, Grand Island, NY, USA) for Bcl-2 for the respective measurements. The experimental procedures were conducted according to the instructions provided by the manufacturers and our recently published article [31,34,36,37]. Initially, HepG2 cells were cultured in triplicate in 96-well plates. Following a 24-h incubation period, the cells were subjected to various concentrations of compound **5k**, while a control group was treated with 0.1% DMSO (*V*/*V*). After an additional 24-h incubation, the absorbance levels were assessed using a BioTek Synergy H1 Multi-mode Reader (Agilent Technologies, Santa Clara, CA, USA) at wavelengths ranging from 450 to 650 nm.

### 3.4. In Silico Studies

#### 3.4.1. Molecular Docking

Molecular docking analysis of compound **5k** with the ATP active binding pockets of CDK2, EGFR, Her2, and VEGFR2 were studied according to the previously described methods [34,37]. The X-ray crystal structures of the EGFR kinase domain bound to erlotinib (PDB ID: 4HJO), the VEGFR2 kinase domain bound to sorafenib (PDB ID: 4ASD), the kinase domain of human Her2 bound to TAK-285 (PDB ID: 3RCD), and the CDK2 kinase bound to sunitinib were acquired from The Protein Data Bank (http://www.rcsb.org, (accessed on 5 June 2023). For docking studies, Discovery Studio, AutoDock Tools, Vina, and PyRx (The Scripps Research Institute, La Jolla, CA, USA) software programs were employed. First, all additional molecules, such as water, ligands, and sulfate, were removed from the downloaded protein crystal structures and the obtained files were saved as PDB format. Second, the previously saved files were converted to PDBQT format after adding polar hydrogens by using AutoDock Tools. Third, the co-crystallized ligands were separated by Discovery Studio saved in a PDB file and converted to PDBQT file by using AutoDock Tools. Finally, PyRx was utilized to conduct the docking simulations. The best pose of compound **5K** superimposed with the reference standard positive control was exploited to investigate the possible binding interactions with the target kinase enzymes.

#### 3.4.2. In Silico ADMET Studies

The in silico pkCSM descriptors algorithm protocol was utilized to predict the absorption, distribution, metabolism, elimination, and toxicity (ADMET) profiles of the chosen synthesized compounds, following the methodology described in the referenced study [33].

## 4. Conclusions

In conclusion, the findings of this study demonstrate that the halogenated “(*E*)-4-((7*H*-Pyrrolo[2,3-d]pyrimidin-4-yl)amino)-*N*’-benzylidenebenzohydrazide” compounds possess excellent cytotoxic properties against various cancer cell lines and possess the ability to inhibit multiple tyrosine kinases. These results establish them as highly promising candidates for further development as kinase inhibitors, holding great potential for innovative therapeutic approaches targeting diseases characterized by abnormal cell death, particularly cancer. The study underscores the significance of incorporating halogenated-Pyrrolo[2,3-d]pyrimidin tyrosine kinase inhibitors in the development of effective anticancer therapies and introduces a novel compound with prospects for future clinical optimization. Further investigations are warranted to unravel the mechanisms of action underlying the efficacy of compound **5k** and to evaluate its therapeutic applications. Moreover, it is important to emphasize that the synthesis of these compounds is straightforward, high yielding, and does not involve chromatographic purification or complex reagents, including metal-containing ones. This advantageous synthesis route facilitates their potential for industrial-scale production, ensuring a viable pathway for future development and potential clinical translation. Further research is necessary to assess the in vivo efficacy and safety of these compounds, paving the way for their potential utilization in clinical settings.

## Figures and Tables

**Table 1:** The cytotoxicity of compounds **5a**–**m** against specific cancer cell lines in-vitro.

Compound		IC_50_ (µM) *			
No.	R	HepG2	HeLa	MDA-MB-231	MCF-7
**5a**	H	43.15 ± 2.7	68.17 ± 3.3	56.21 ± 3.2	64.90 ± 3.6
**5b**	2-Br	53.21 ± 3.1	90.58 ± 4.7	79.23 ± 4.1	83.94 ± 4.3
**5c**	3-Br	48.53 ± 2.9	85.47 ± 4.1	75.84 ± 3.9	80.69 ± 4.0
**5d**	4-Br	62.42 ± 3.5	95.76 ± 5.1	81.46 ± 4.0	88.23 ± 4.5
**5e**	2-Cl	29.12 ± 2.5	52.96 ± 2.9	48.15 ± 2.6	55.33 ± 3.1
**5f**	3-Cl	74.61 ± 3.9	>100	86.62 ± 4.6	94.73 ± 4.9
**5g**	4-Cl	51.92 ± 3.0	79.71 ± 3.9	67.56 ± 3.7	73.34 ± 3.9
**5h**	2-F	33.20 ± 2.4	49.88 ± 2.8	45.25 ± 2.5	52.44 ± 2.9
**5i**	3-F	45.03 ± 2.8	76.12 ± 3.7	63.77 ± 3.5	69.89 ± 3.8
**5j**	4-F	42.65 ± 2.8	71.70 ± 3.5	58.45 ± 3.3	66.36 ± 3.6
**5k**	3,4-di-Cl	41.54 ± 2.6	57.82 ± 3.1	51.44 ± 2.8	59.07 ± 3.5
**5l**	2-OH,5-Br	36.30 ± 2.6	46.07 ± 2.4	41.32 ± 2.4	47.06 ± 2.5
**5m**	3-CF_3_	39.79 ± 2.7	64.53 ± 3.4	53.71 ± 3.0	61.86 ± 3.3
**Sunitinib**		11.35 ± 1.0	21.84 ± 1.7	19.04 ± 1.5	24.83 ± 1.8

* IC_50_ values are the mean ± SD of triplicate measurements.

**Table 2:** Effects of **5e**, **5h**, **5k**, **5l** on the levels of protein kinases.

Compound	IC_50_ (nM) *			
	EGFR	Her2	VEGFR2	CDK2
**5e**	1144	335	593	2118
**5h**	636	206	1044	1023
**5k**	79	40	136	204
**5l**	406	193	1201	599
**Sunitinib**	93	74	261	141
**Erlotinib**	55	-	-	-
**Staurosporine**	-	38	-	281
**Sorafenib**	-	-	41	-

* IC_50_ values are the mean ± SD of triplicate measurements.

**Table 3:** Analysis of cell cycle in HepG2 cells treated with compound **5k**.

Compound/Cell Line	DNA Content (%)			Cell Cycle Distribution Index (CDI)
	%G0–G1	%S	%G2/M	
HepG2	43.86	36.76	19.38	1.35
**5k**/HepG2	49.18	32.52	18.3	1.03

**Table 4:** Analysis of the distribution of apoptotic cells in HepG2 cells treated with compound **5k**.

Sample	Apoptosis			Necrosis
	Total	Early	Late	
HepG2	2.38	0.51	0.29	1.58
**5k**/HepG2	29.54	15.63	9.74	4.17

**Table 5:** Analysis of expression change of apoptotic proteins following treatment with **5k**.

Kinase Protein	Protein Expression (Pg/mL) *		
	Caspase-3	Bax	Bcl-2
HepG2	66.28 ± 4.66	67.09 ± 4.94	11.7 ± 0.36
**5k**/HepG2	453.8 ± 17.4	173.8 ± 6.61	5.146 ± 0.09
Saurosporine	684.6 ± 13.4	276.9 ± 2.26	4.404 ± 0.09

* Protein expression values are the mean ± SD of triplicate measurements.

**Table 6:** Binding energies of compound **5k** and the respective reference standards positive controls against EGFR, VEGFR2, Her2, and CDK2.

Compound	Binding Scores (Kcal/mol)			
	EGFR	VEGFR2	Her2	CDK2
**5k**	−8.7	−9.3	−8.9	−8.7
Reference standards ^a^	−9.1	−11.9	−10.1	−10.1

^a^ Erlotinib for EGFR, sorafenib for VEGFR2, TAK-285 for Her2 and sunitinib CDK2.

**Table 7:** In silico ADMET results of the most active compounds and Sorafenib.

Parameters		5e	5h	5k	5l	Sorafenib
	Compound					
**Molecular properties**						
Molecular weight		390.834	374.379	425.279	372.388	464.831
LogP		4.1188	3.6045	4.7722	3.171	5.5497
H-acceptor		5	5	5	6	4
H-donors		3	3	3	4	3
Surface area		165.036	158.898	175.339	159.527	185.111
**Absorption**						
Water solubility (log mol/L)		−3.562	−3.533	−3.55	−3.439	−5.058
Intestinal absorption (human)		86.295%	85.4%	87.539%	78.832%	84.985%
Skin permeability		−2.739	−2.739	−2.738	−2.738	−2.765
**Distribution**						
BBB permeability		−1.356	−1.395	−1.536	−1.37	−1.675
CNS permeability		−2.231	−2.384	−2.123	−2.535	−1.995
**Metabolism**						
CYP2D6 substrate		Yes	Yes	Yes	Yes	No
CYP3A4 substrate		Yes	Yes	Yes	Yes	Yes
Excretion						
Total clearance (log mL/min/kg)		0.01	0.245	0.016	0.348	0.208
Renal OCT2 substrate		No	No	No	No	No
**Toxicity**						
Max. tolerated dose (human) (log mg/kg/day)		0.374	0.374	0.403	0.41	0.383
Oral rate acute toxicity (LD50) (mol/kg)		2.931	2.906	2.919	2.884	2.299
Oral rate chronic toxicity (LOAEL) (log mg/kg_bw/day)		2.121	2.202	1.826	2.291	0.848
Hepatotoxicity		Yes	Yes	Yes	Yes	Yes
Skin sensitization		No	No	No	No	No

## Data Availability

Data are given in the supporting file and are available upon request.

## Supplementary Materials

The following supporting information can be downloaded at: https://www.mdpi.com/article/10.3390/ph16091324/s1, ^1^H-NMR, ^13^CNMR and Mass spectra.
