# Supplementary material for: Discovery of New Pyrrolo[2,3-d]pyrimidine Derivatives as Potential Multi-Targeted Kinase Inhibitors and Apoptosis Inducers
Source: Pharmaceuticals (Basel). 2023 Sep 19;16(9):1324. doi: 10.3390/ph16091324 (PMC10536928; doi:10.3390/ph16091324)

Discovery of New Pyrrolo[2,3-d]pyrimidine Derivatives as Potential Multi-Targeted Kinase Inhibitors and Apoptosis Inducers

AbdulAziz A. Alotaibi†, Mohammed M. Alanazi*,† and A. F. M. Motiur Rahman*

Department of Pharmaceutical Chemistry, College of Pharmacy, King Saud University, Riyadh 11451, Saudi Arabia

*Corresponding Authors: mmalanazi@ksu.edu.sa ; afmrahman@ksu.edu.sa

†These authors contributed equally to this work

Entry	Content	Page no.
**3**	^{1}H-NMR spectra of **3**	4
**3**	^{1}H-NMR spectra of **3**	5
**3**	^{13}C-NMR spectra of **3**	6
**3**	Mass spectra of **3**	7
**3**	HRMS Mass spectra of **3**	8
**4**	^{1}H-NMR spectra of **4**	9
**4**	^{13}C-NMR spectra of **4**	10
**4**	Mass spectra of **4**	11
**4**	HRMS Mass spectra of **4**	12
**5a**	^{1}H-NMR spectra of **5a**	13
**5a**	^{13}C-NMR spectra of **5a**	14
**5a**	Mass spectra of **5a**	15
**5a**	HRMS Mass spectra of **5a**	16
**5b**	^{1}H-NMR spectra of **5b**	17
**5b**	^{13}C-NMR spectra of **5b**	18
**5b**	Mass spectra of **5b**	19
**5b**	HRMS Mass spectra of **5b**	20

Entry	Content	Page no.
**5c**	^{1}H-NMR spectra of **5c**	21
**5c**	^{13}C-NMR spectra of **5c**	22
**5c**	Mass spectra of **5c**	23
**5c**	HRMS Mass spectra of **5c**	24
**5d**	^{1}H-NMR spectra of **5d**	25
**5d**	^{13}C-NMR spectra of **5d**	26
**5d**	Mass spectra of **5d**	27
**5d**	HRMS Mass spectra of **5d**	28
**5e**	^{1}H-NMR spectra of **5e**	29
**5e**	^{13}C-NMR spectra of **5e**	30
**5e**	Mass spectra of **5e**	31
**5e**	HRMS Mass spectra of **5e**	32
**5f**	^{1}H-NMR spectra of **5f**	33
**5f**	^{13}C-NMR spectra of **5f**	34
**5f**	Mass spectra of **5f**	35
**5f**	HRMS Mass spectra of **5f**	36
**5g**	^{1}H-NMR spectra of **5g**	37

Entry	Content	Page no.
**5g**	^{13}C-NMR spectra of **5g**	38
**5g**	Mass spectra of **5g**	39
**5g**	HRMS Mass spectra of **5g**	40
**5h**	^{1}H-NMR spectra of **5h**	41
**5h**	^{13}C-NMR spectra of **5h**	42
**5h**	Mass spectra of **5h**	43
**5h**	HRMS Mass spectra of **5h**	44
**5i**	^{1}H-NMR spectra of **5i**	45
**5i**	^{13}C-NMR spectra of **5i**	46
**5i**	Mass spectra of **5i**	47
**5i**	HRMS Mass spectra of **5i**	48
**5j**	^{1}H-NMR spectra of **5j**	49
**5j**	^{13}C-NMR spectra of **5j**	50
**5j**	Mass spectra of **5j**	51
**5j**	HRMS Mass spectra of **5j**	52
**5k**	^{1}H-NMR spectra of **5k**	53
**5k**	^{13}C-NMR spectra of **5k**	54
**5k**	Mass spectra of **5k**	55

Entry	Content	Page no.
**5k**	HRMS Mass spectra of **5k**	56
**5l**	^{1}H-NMR spectra of **5l**	57
**5l**	^{13}C-NMR spectra of **5l**	58
**5l**	Mass spectra of **5l**	59
**5l**	HRMS Mass spectra of **5l**	50
**5m**	^{1}H-NMR spectra of **5m**	51
**5m**	^{13}C-NMR spectra of **5m**	52
**5m**	Mass spectra of **5m**	63
**5m**	HRMS Mass spectra of **5m**	64

^{1}H-NMR spectra of **3**

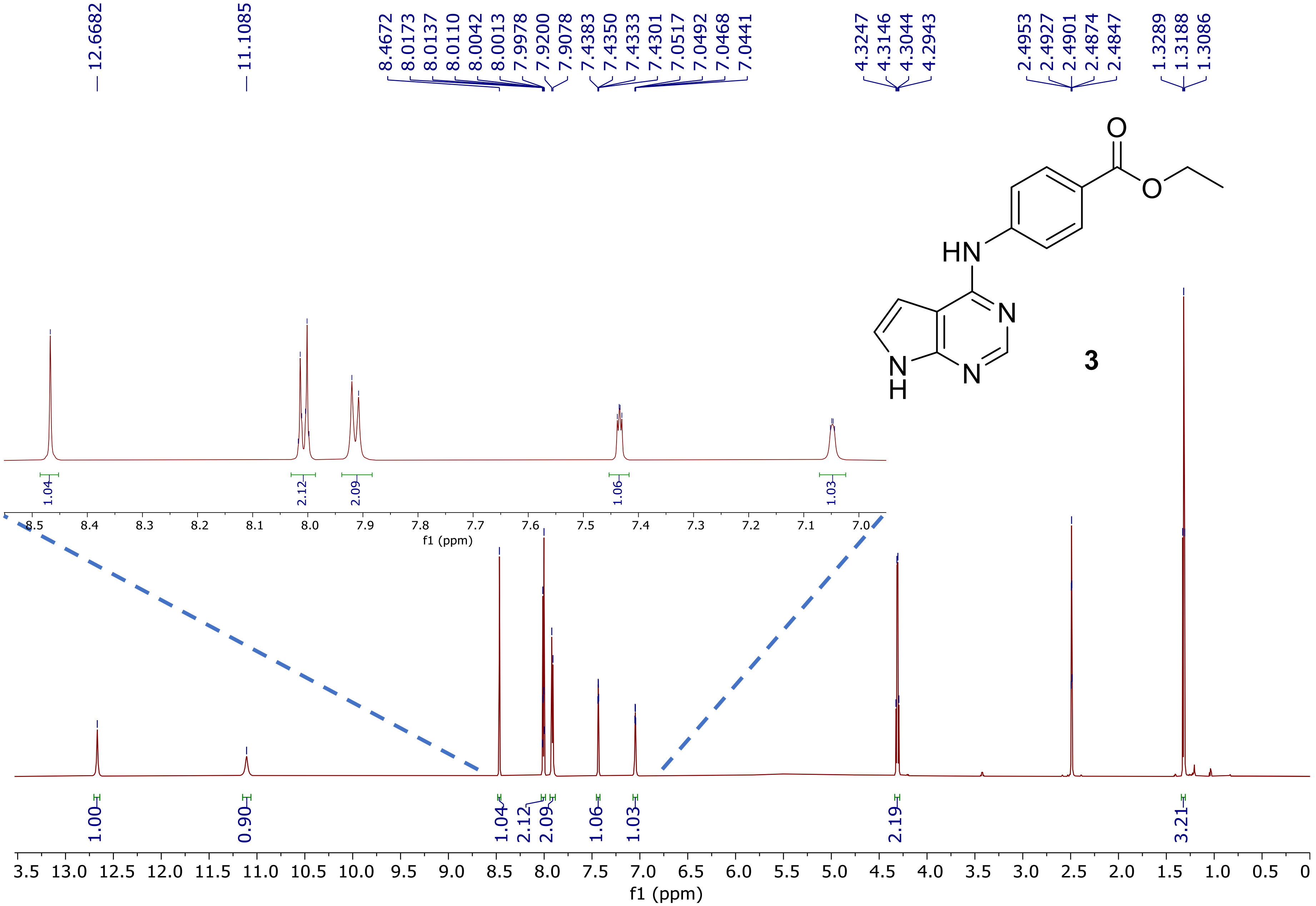

^{13}C-NMR spectra of **3**

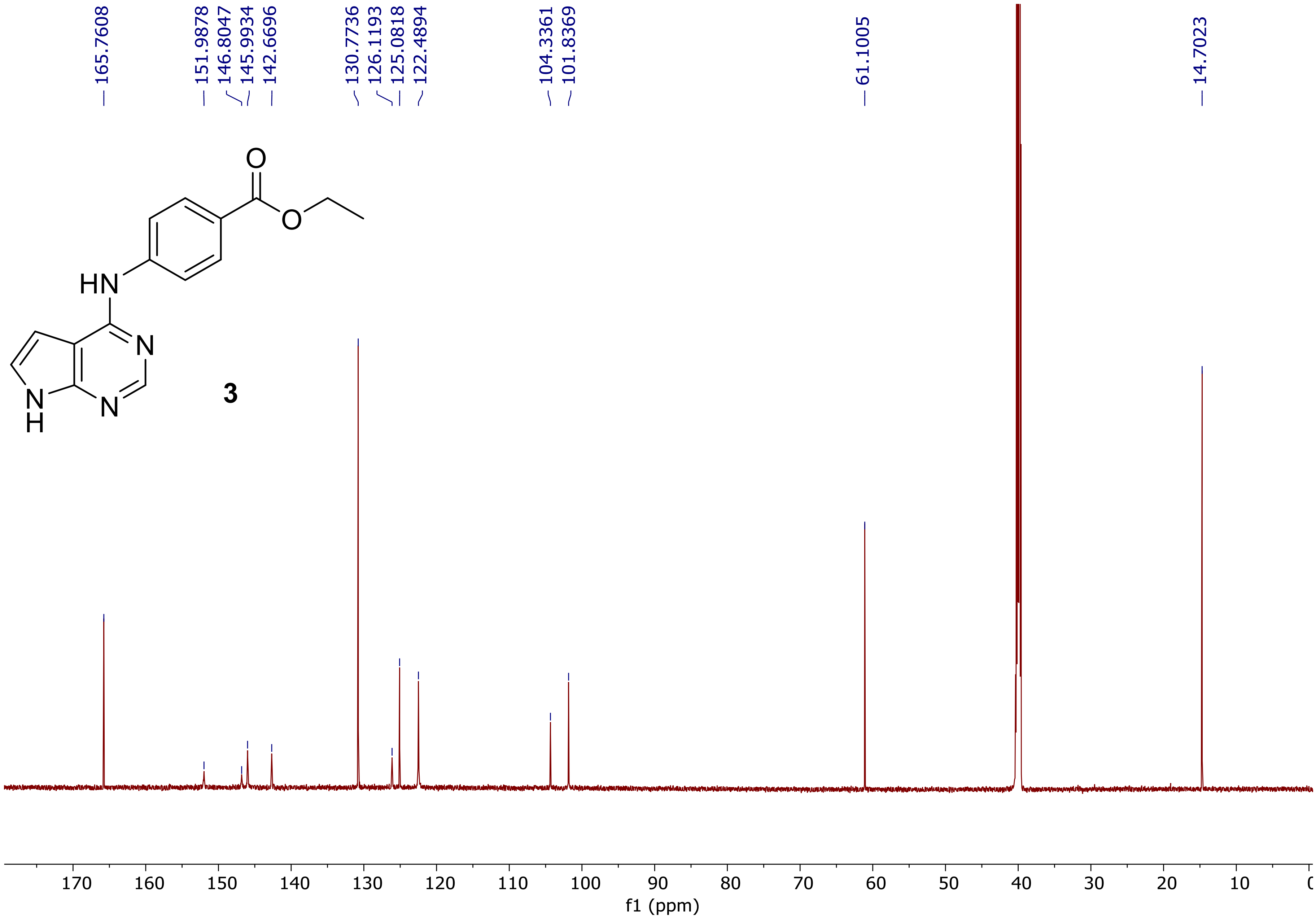

^{13}C-NMR (DEPT135) spectra of 3

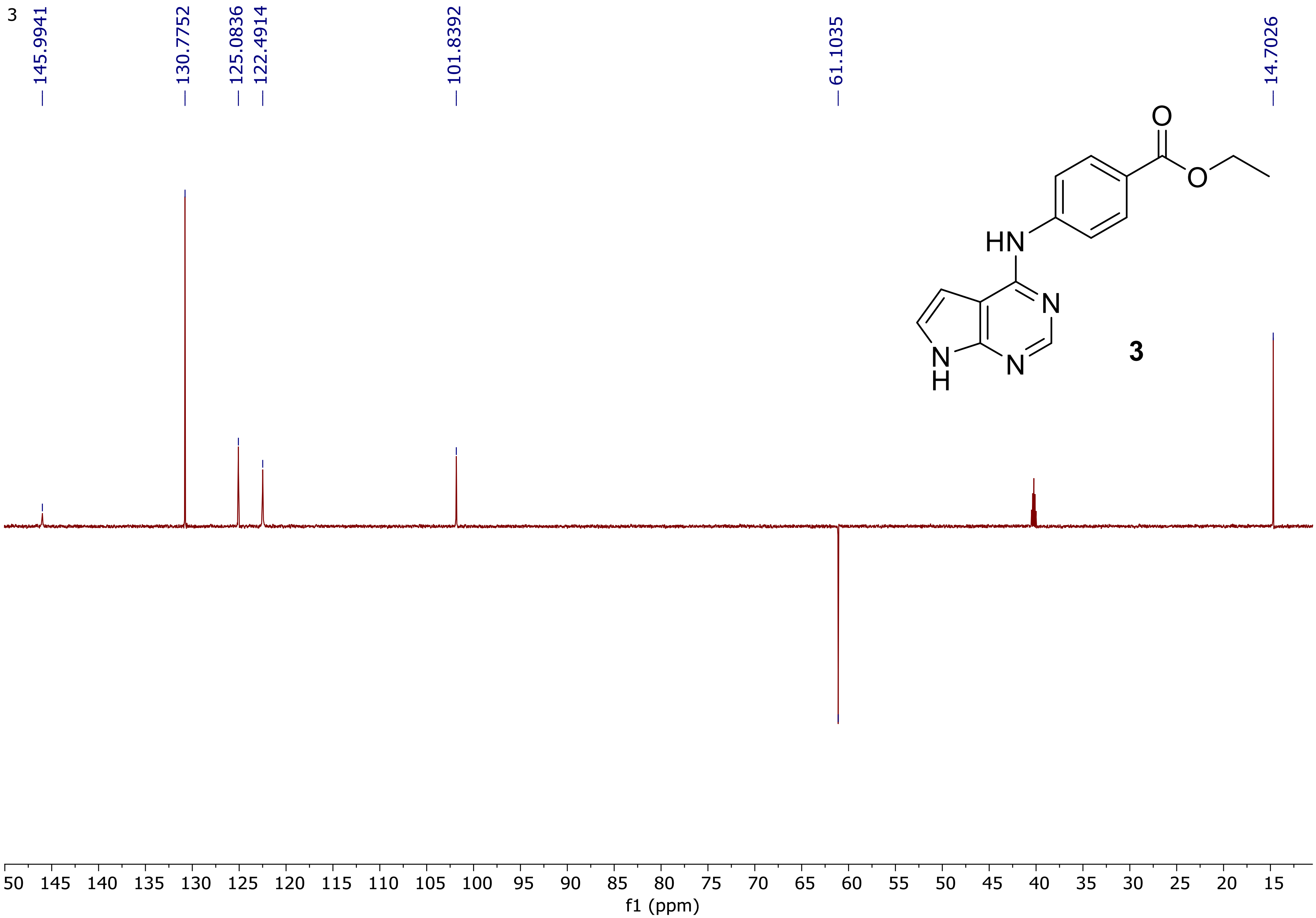

Mass spectra of **3**

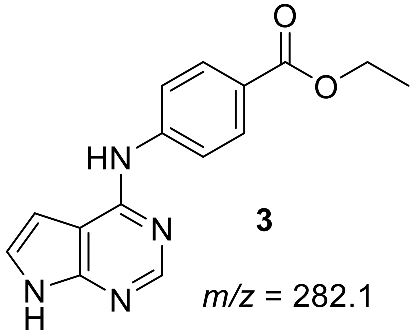

3

m/z = 282.1

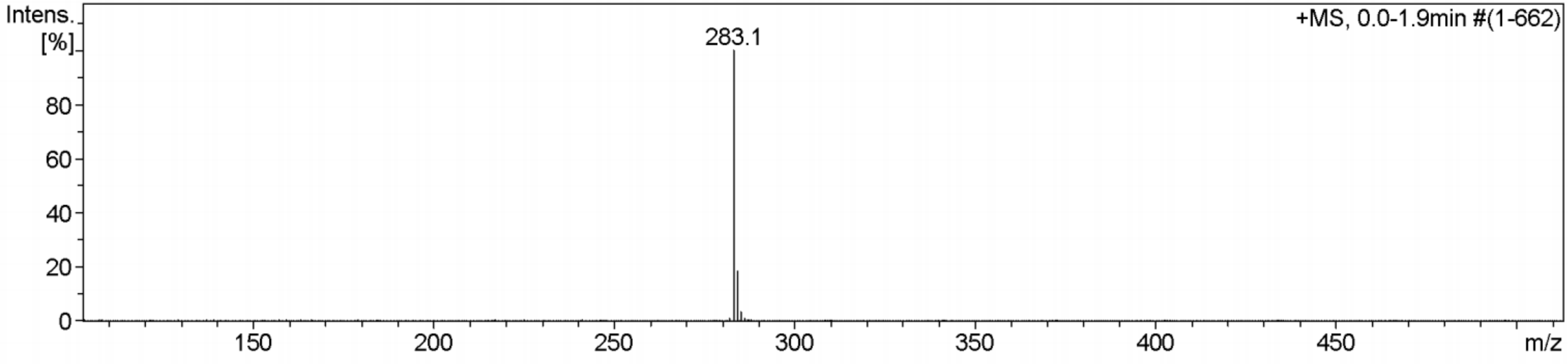

HMRS Mass spectra of **3**

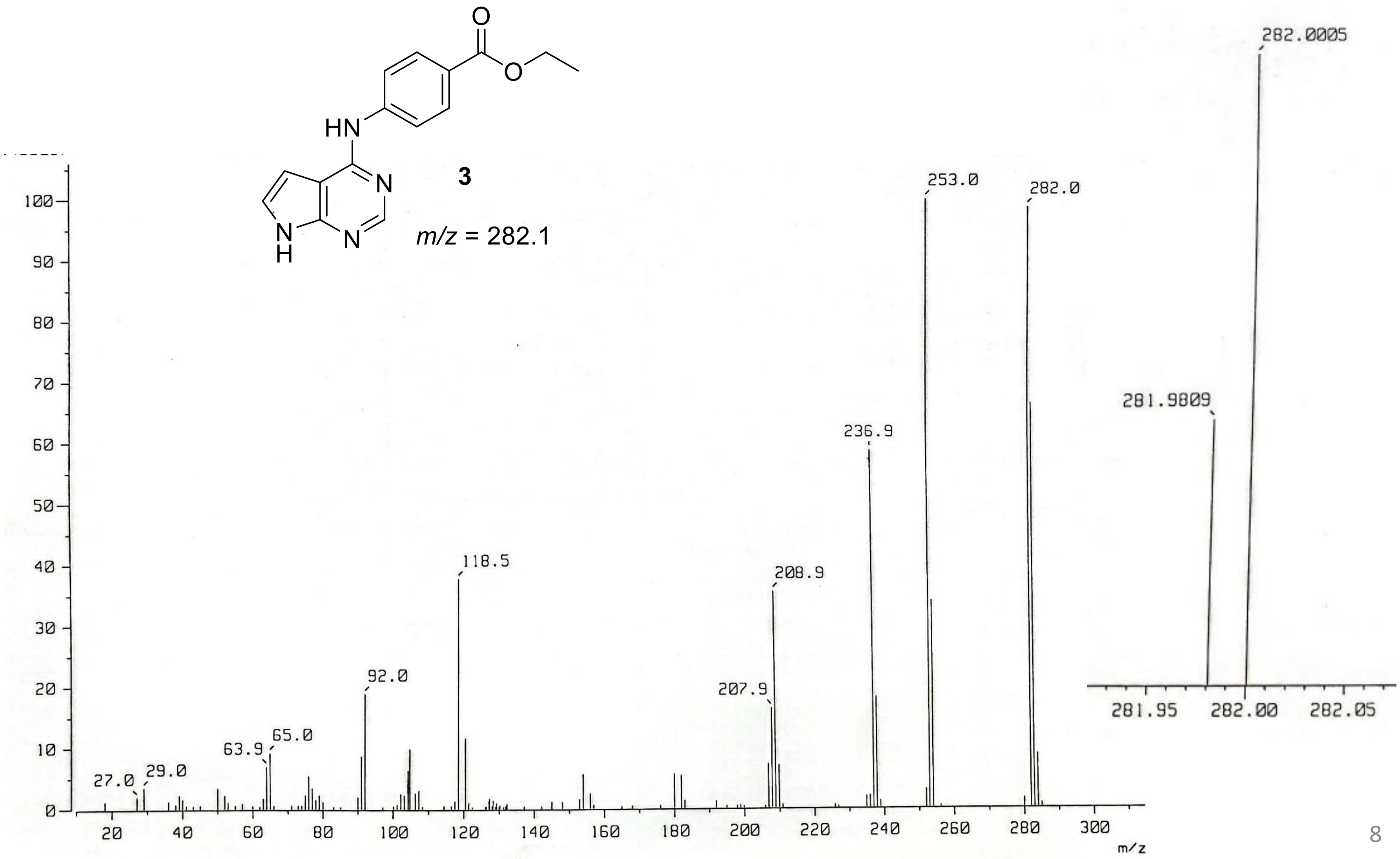

^{1}H-NMR spectra of **4**

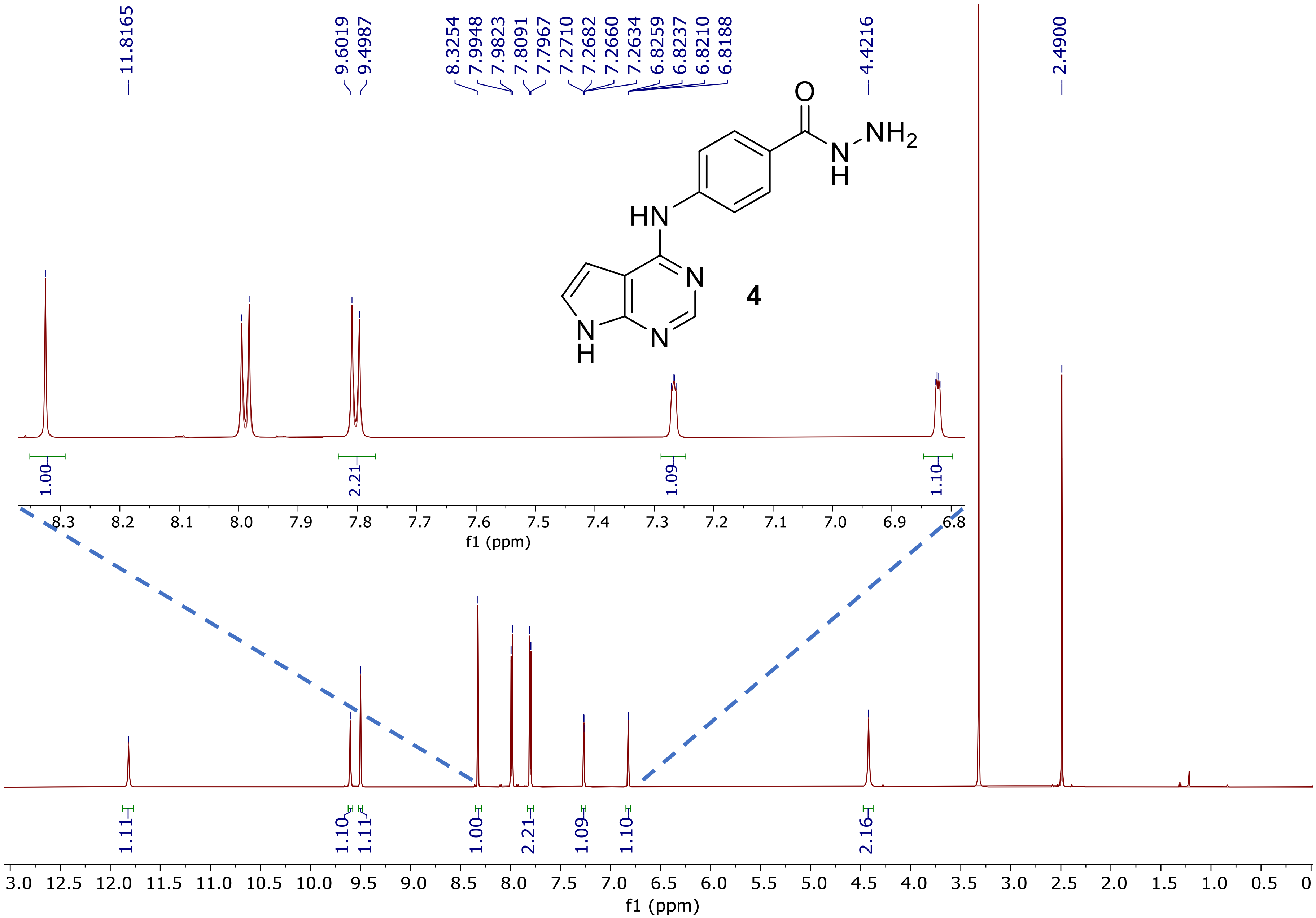

^{13}C-NMR spectra of **4**

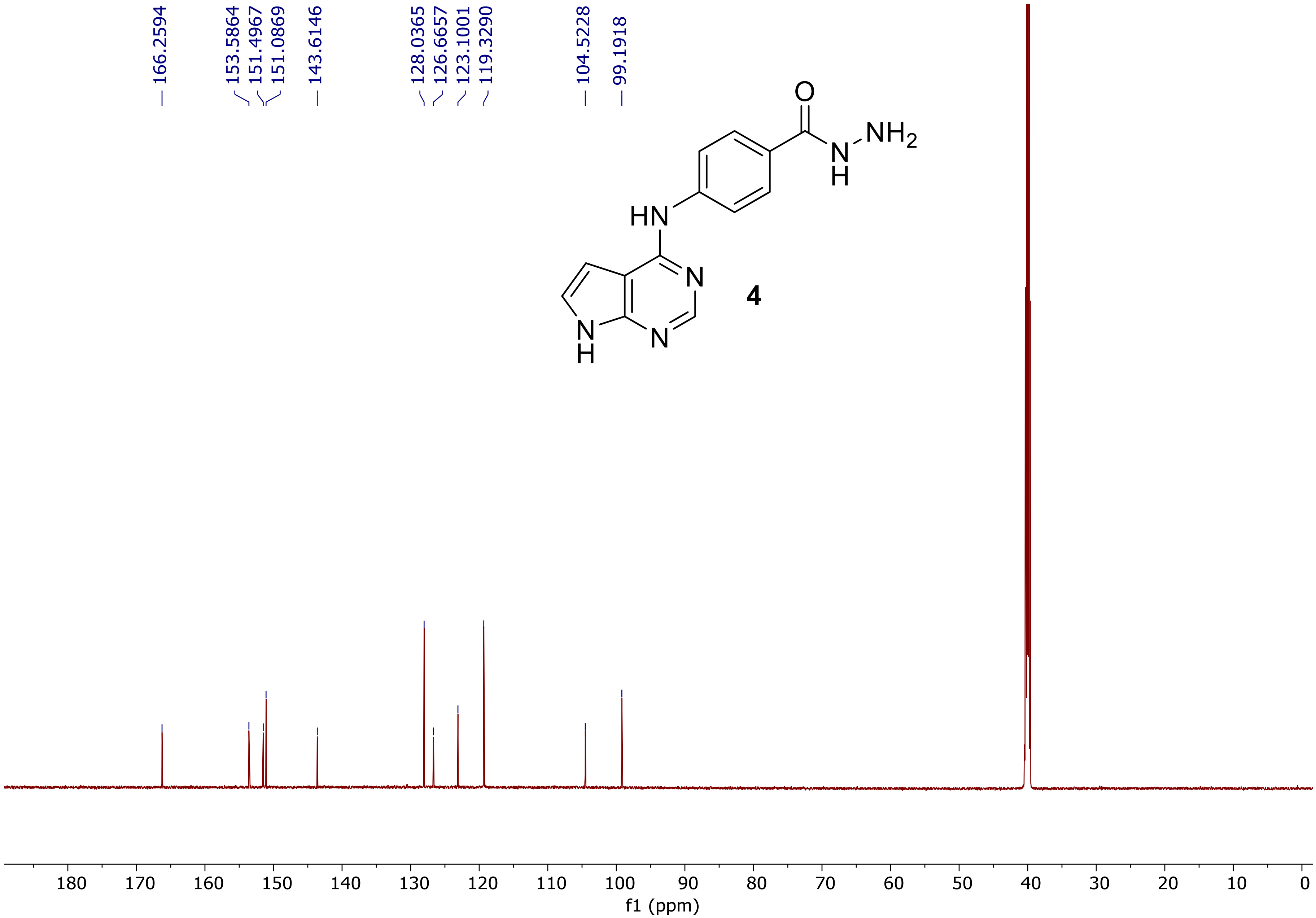

Mass spectra of **4**

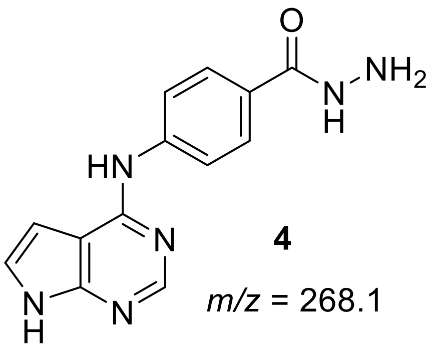

4

m/z = 268.1

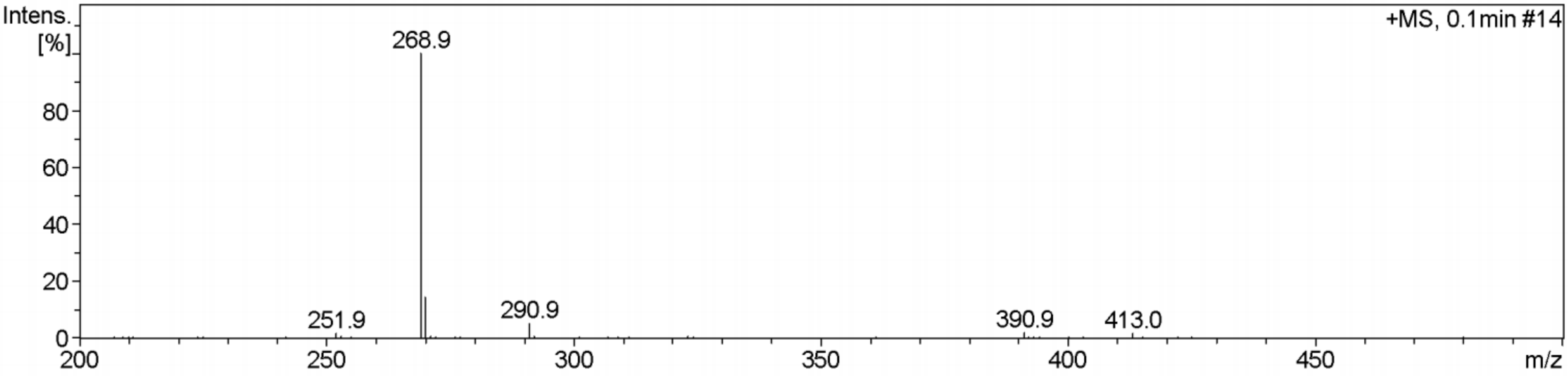

HMRS Mass spectra of **4**

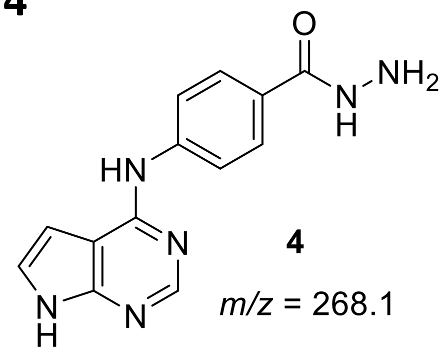

4

m/z = 268.1

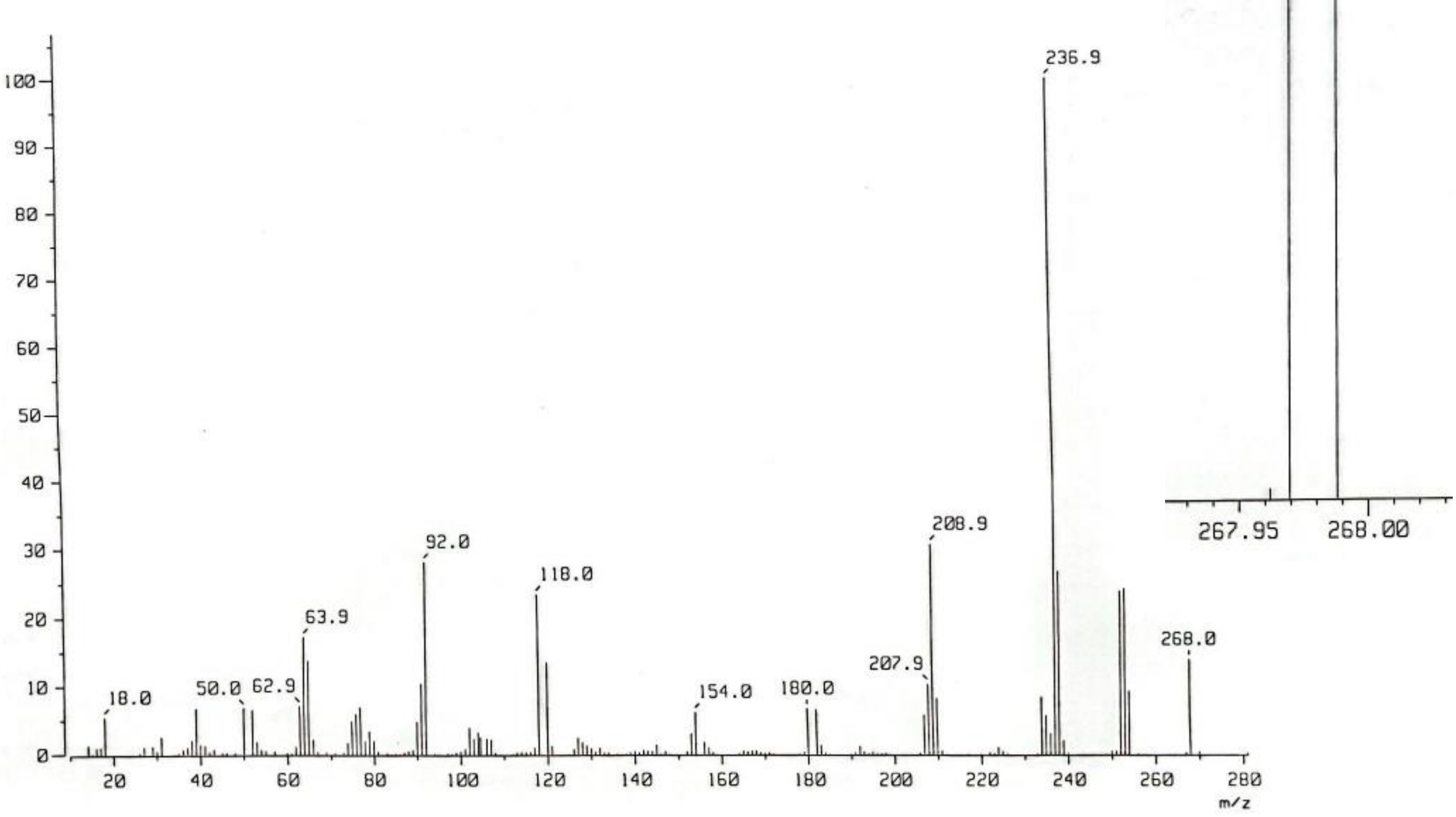

^{1}H-NMR spectra of **5a**

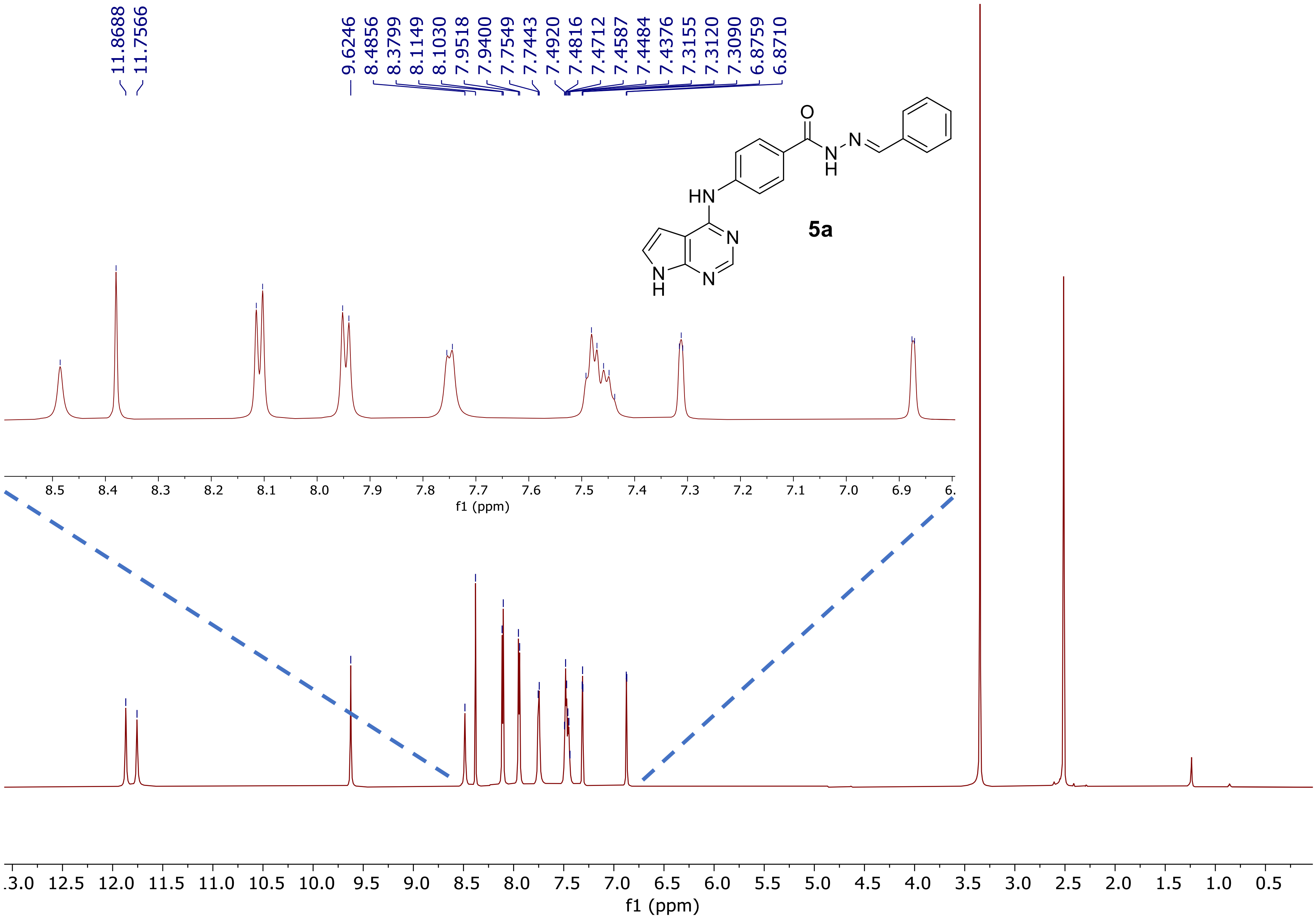

^{13}C-NMR spectra of **5a**

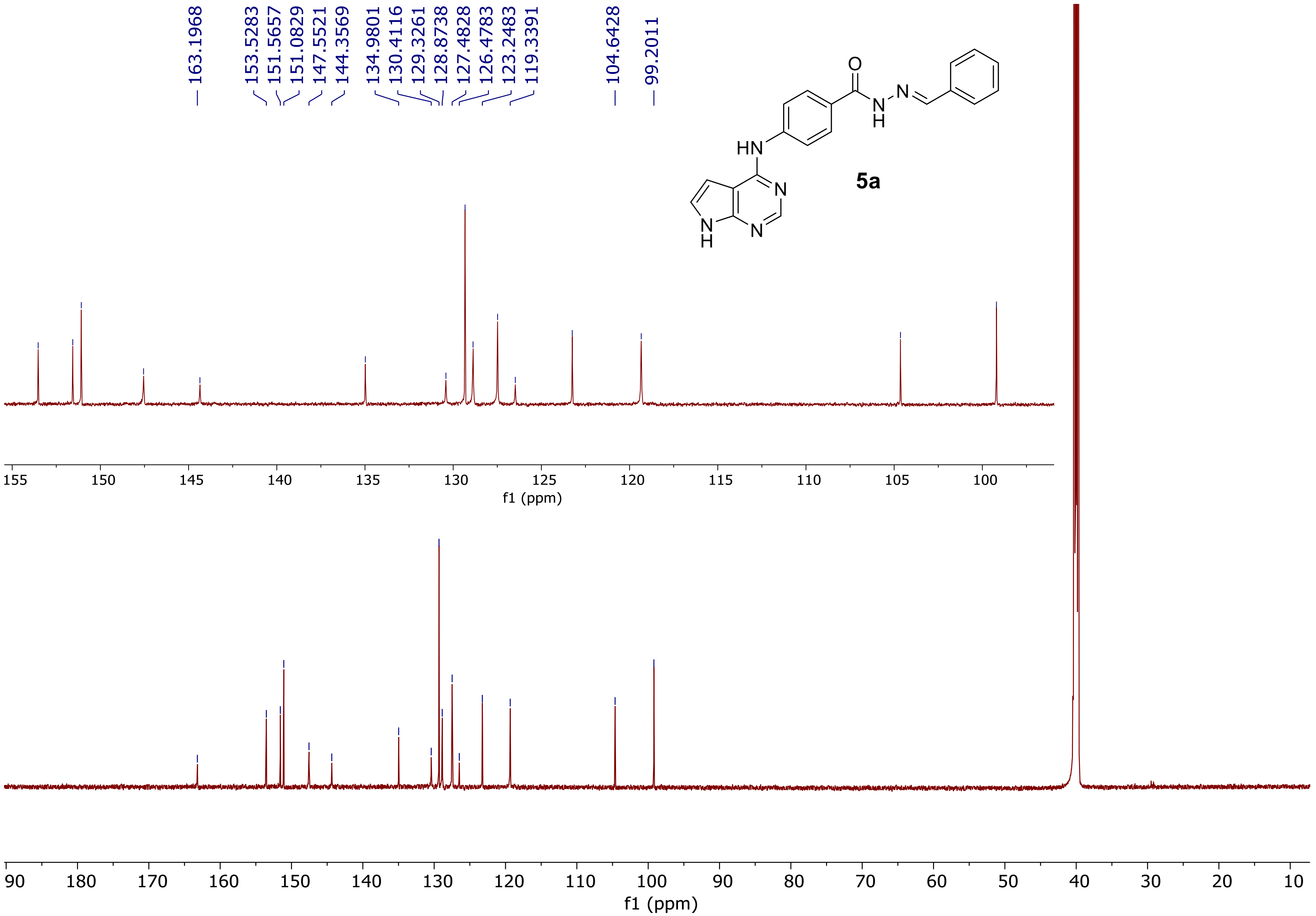

Mass spectra of **5a**

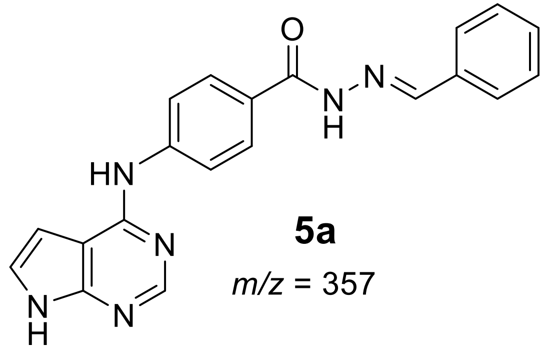

5a

m/z = 357

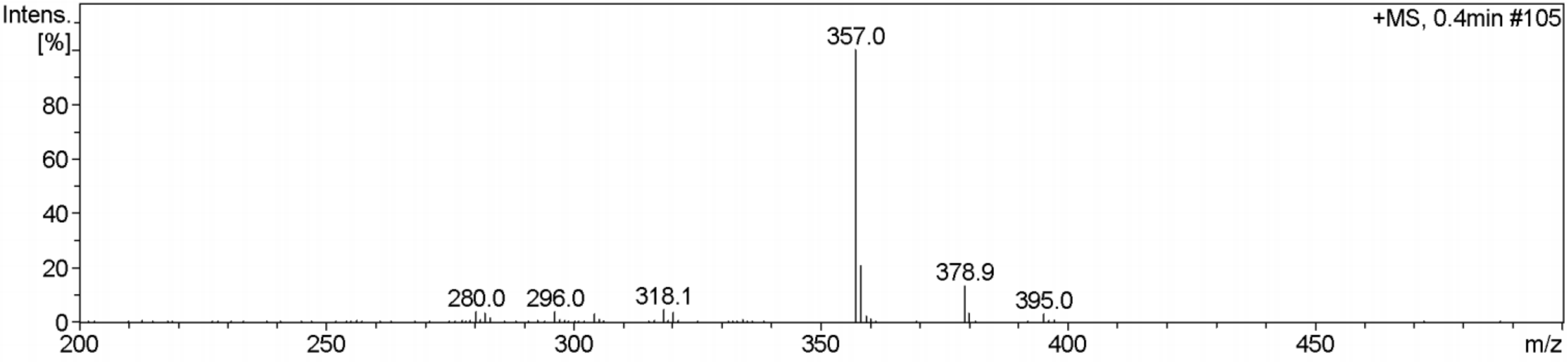

HMRS Mass spectra of **5a**

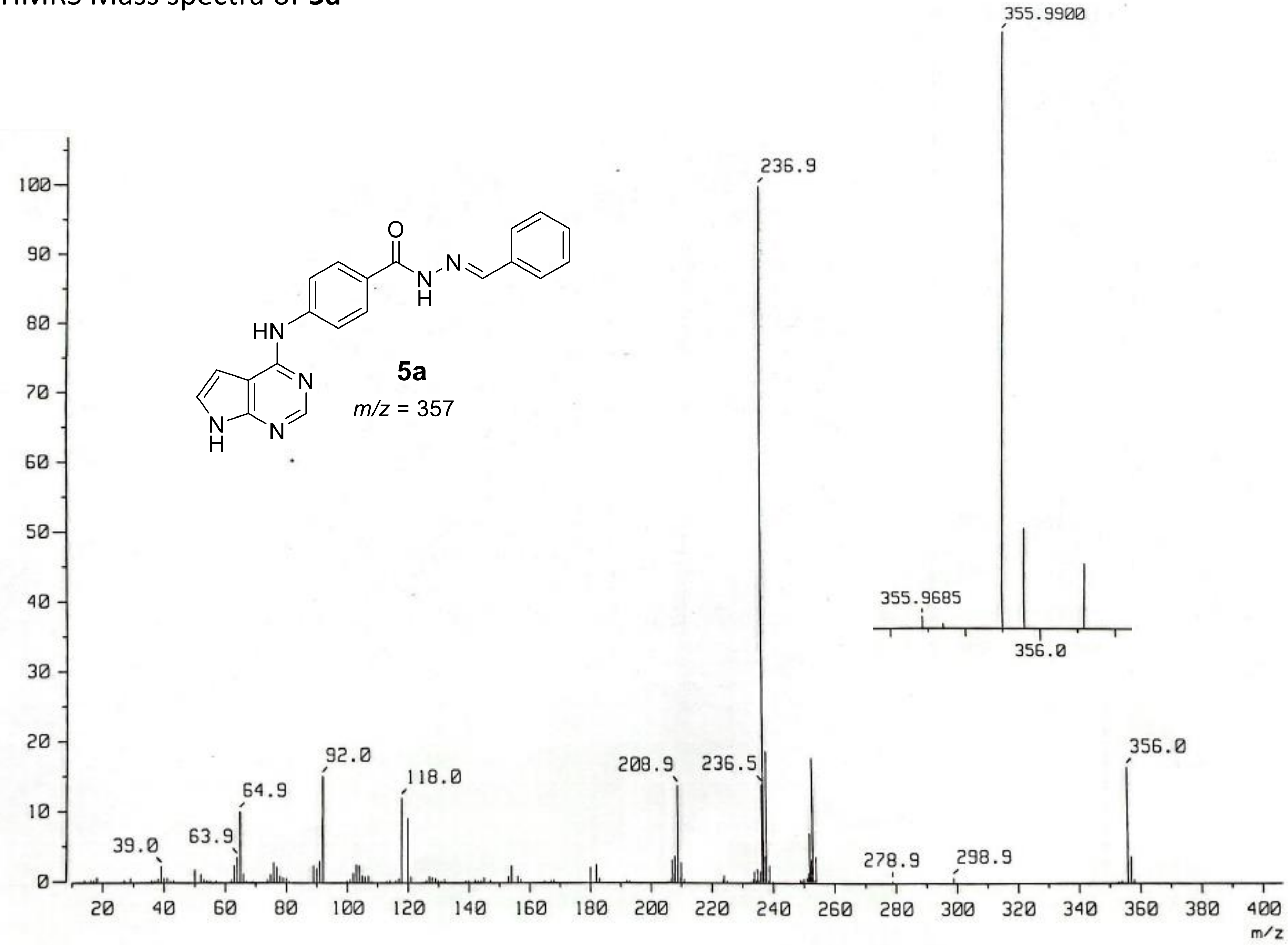

^{1}H-NMR spectra of **5b**

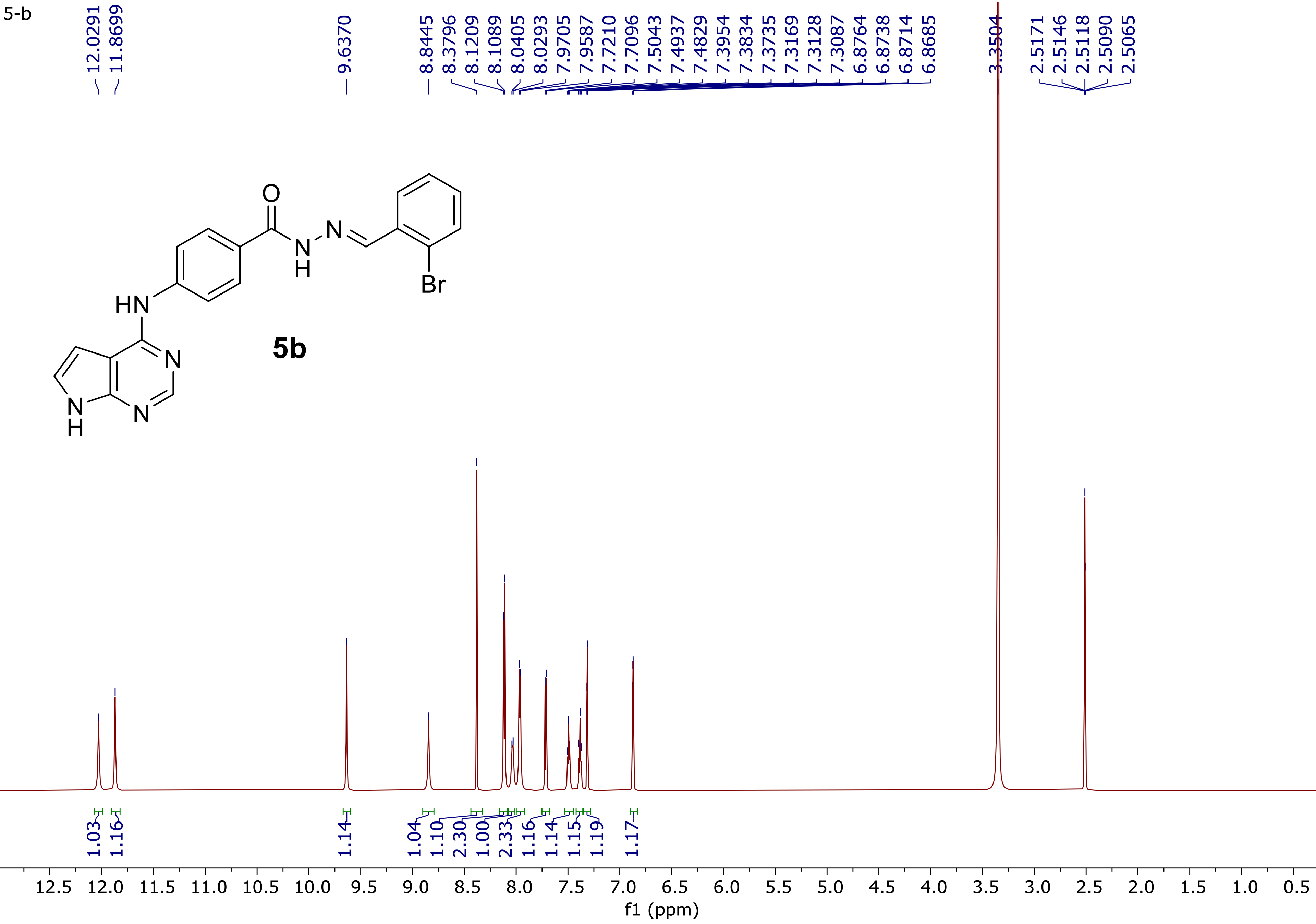

^{13}C-NMR spectra of **5b**

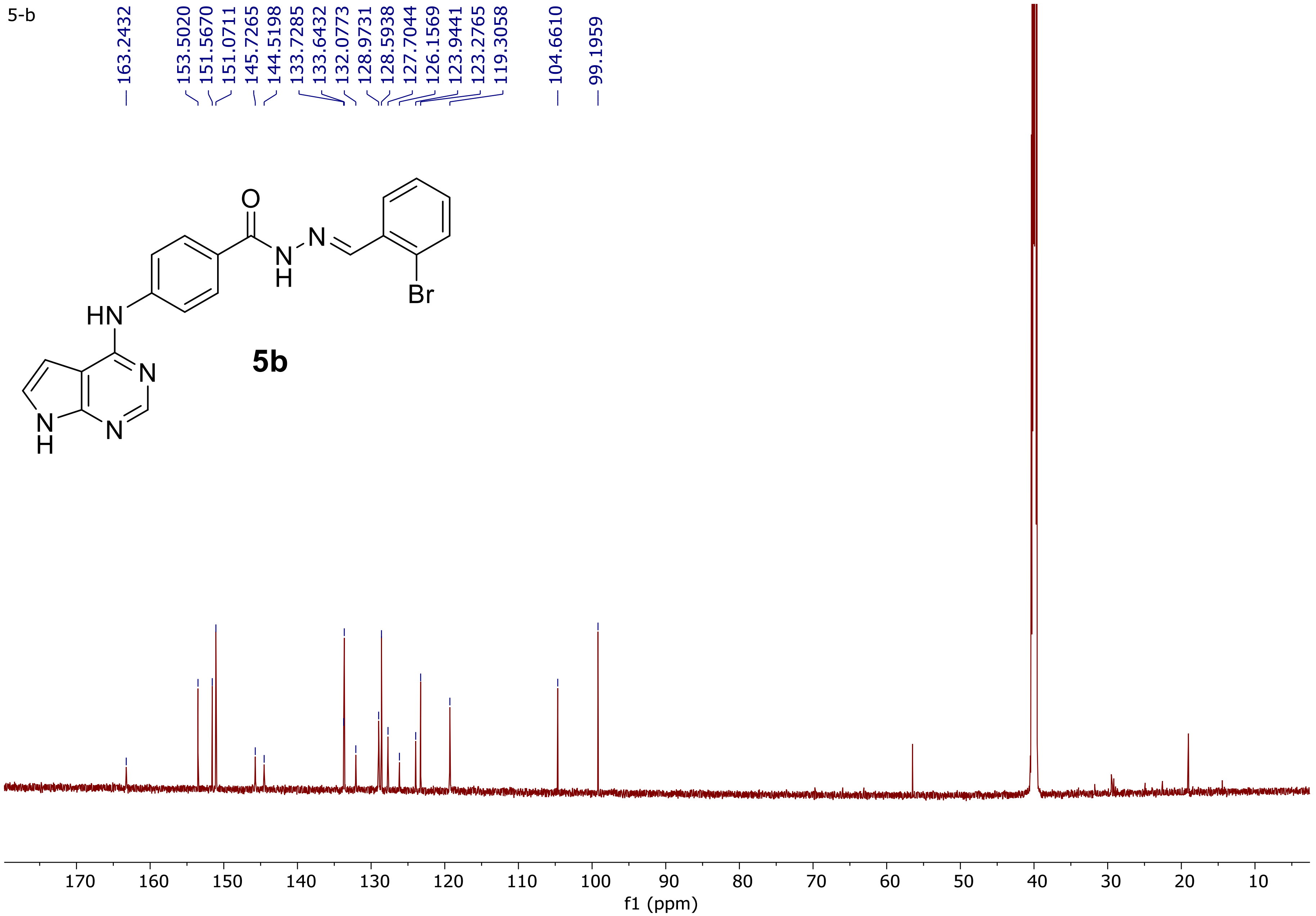

Mass spectra of **5b**

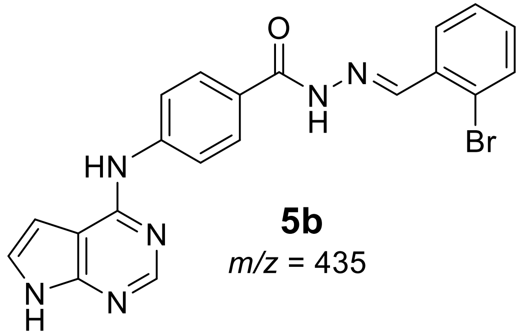

5b
m/z = 435

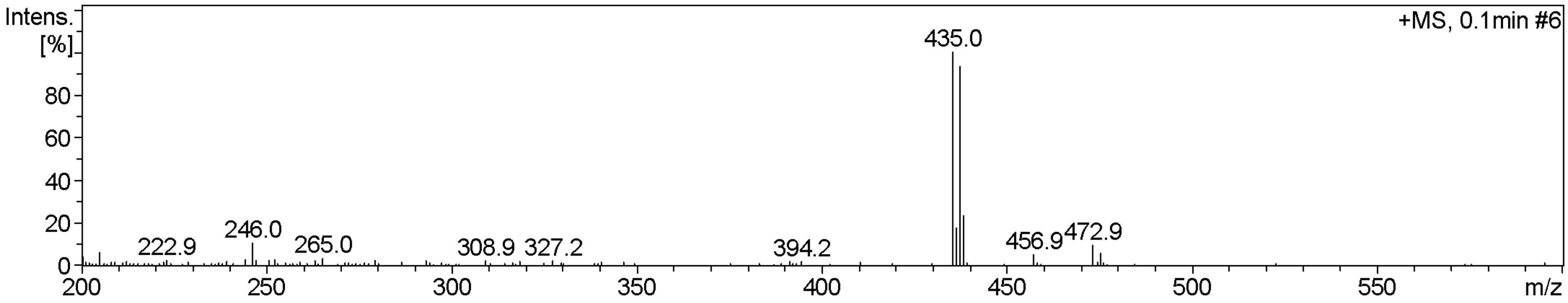

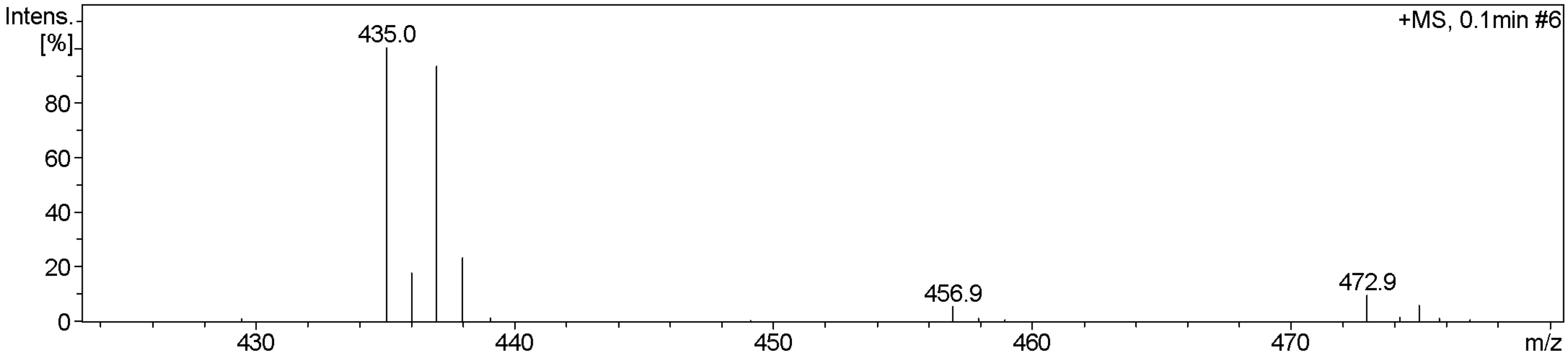

HMRS Mass spectra of **5b**

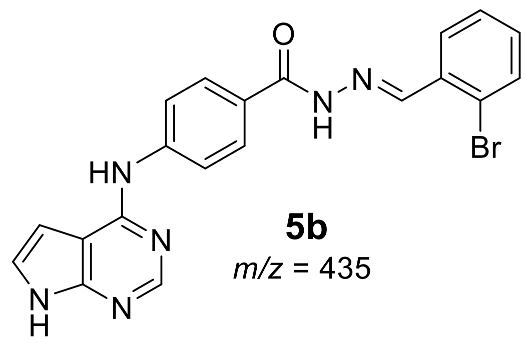

5b

m/z = 435

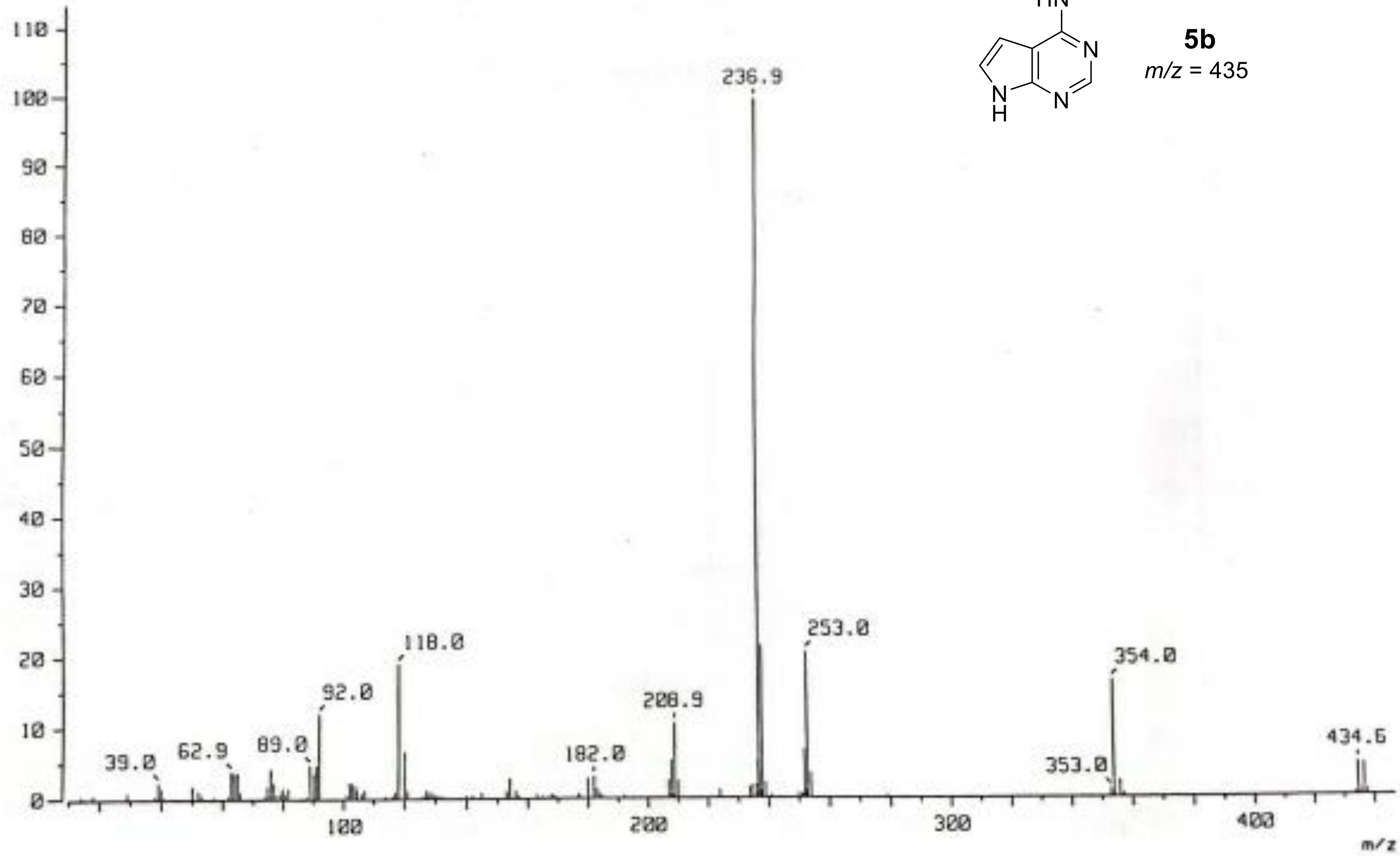

^{1}H-NMR spectra of **5c**

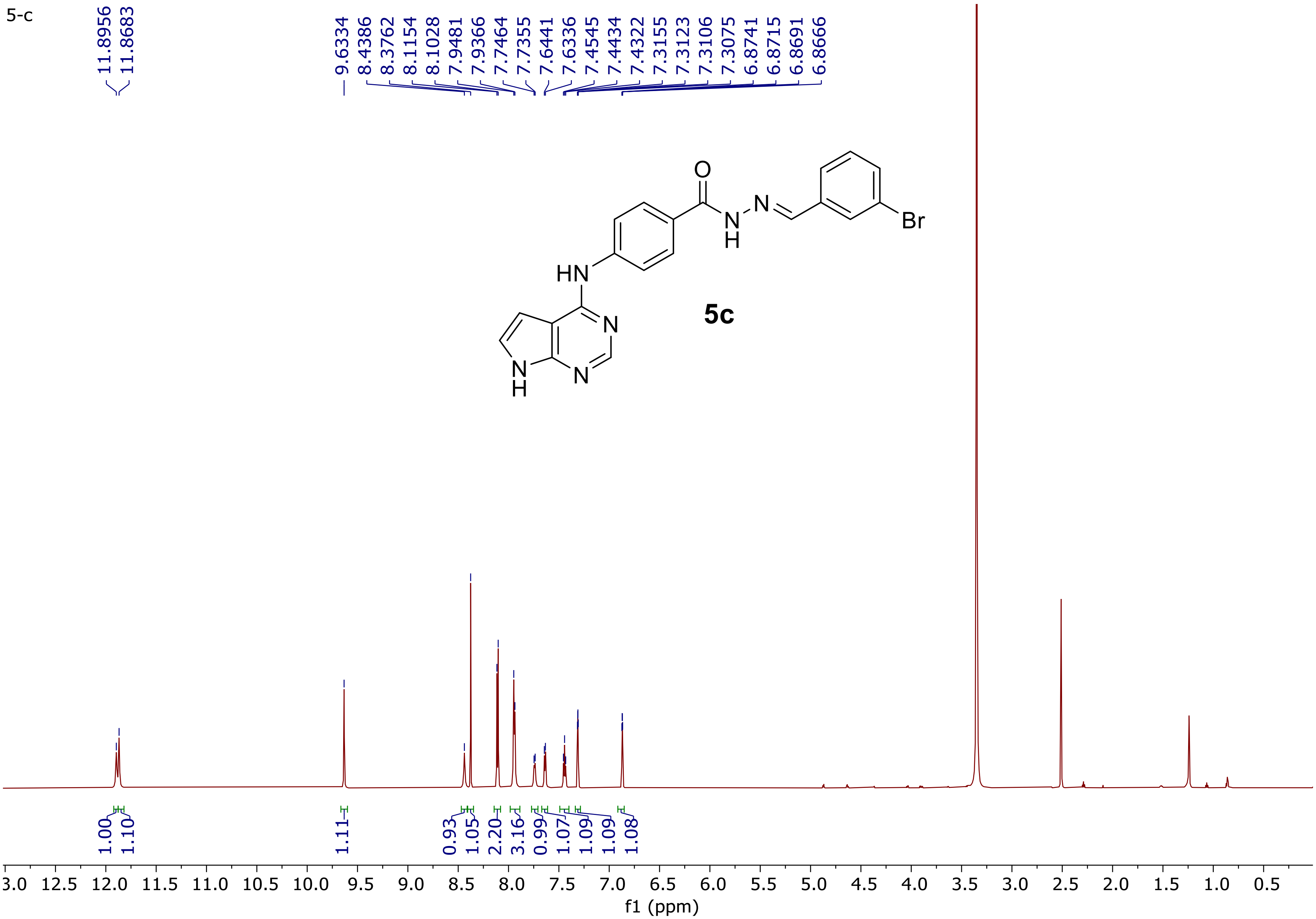

^{13}C-NMR spectra of **5c**

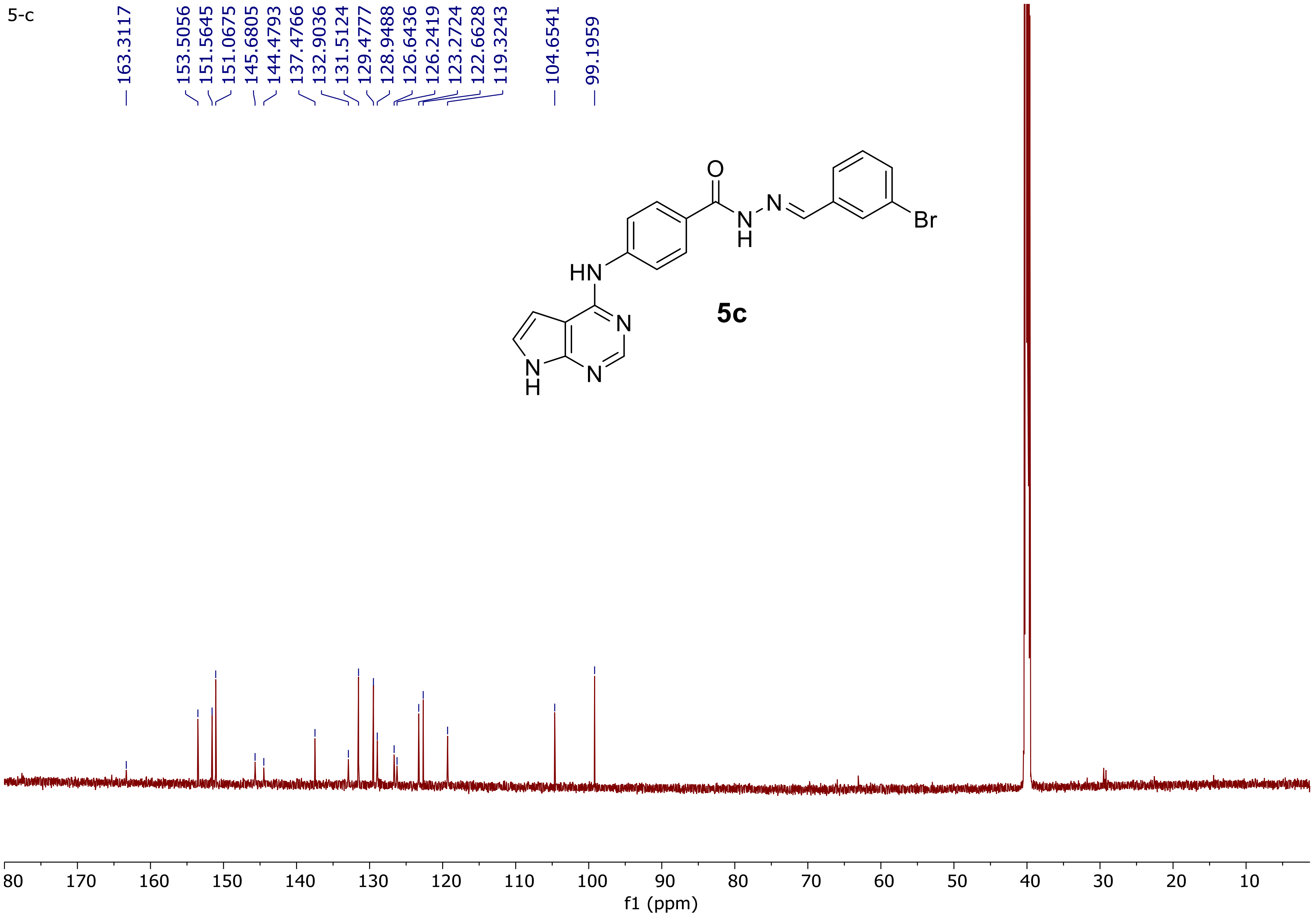

Mass spectra of **5c**

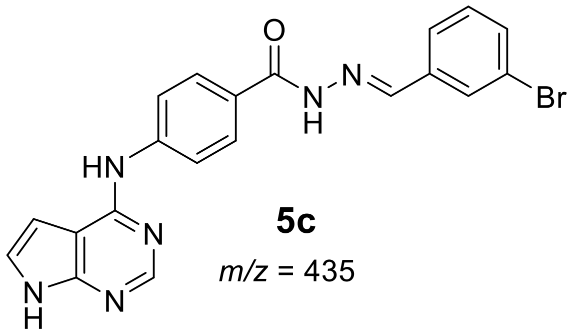

5c
m/z = 435

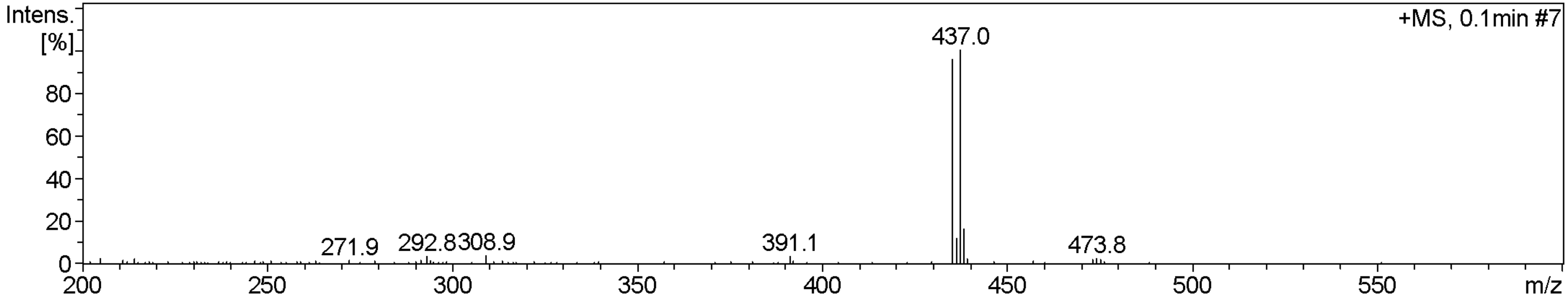

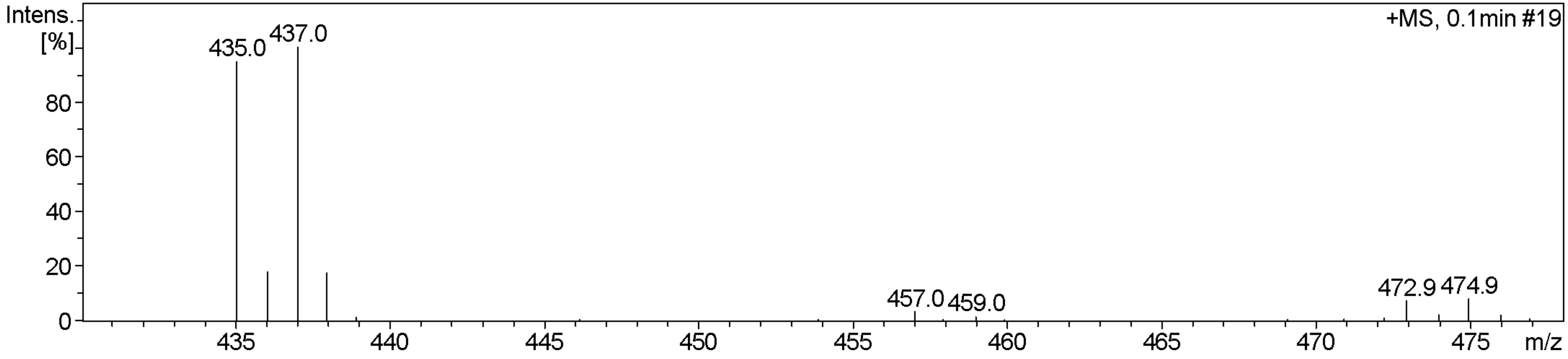

HMRS Mass spectra of **5c**

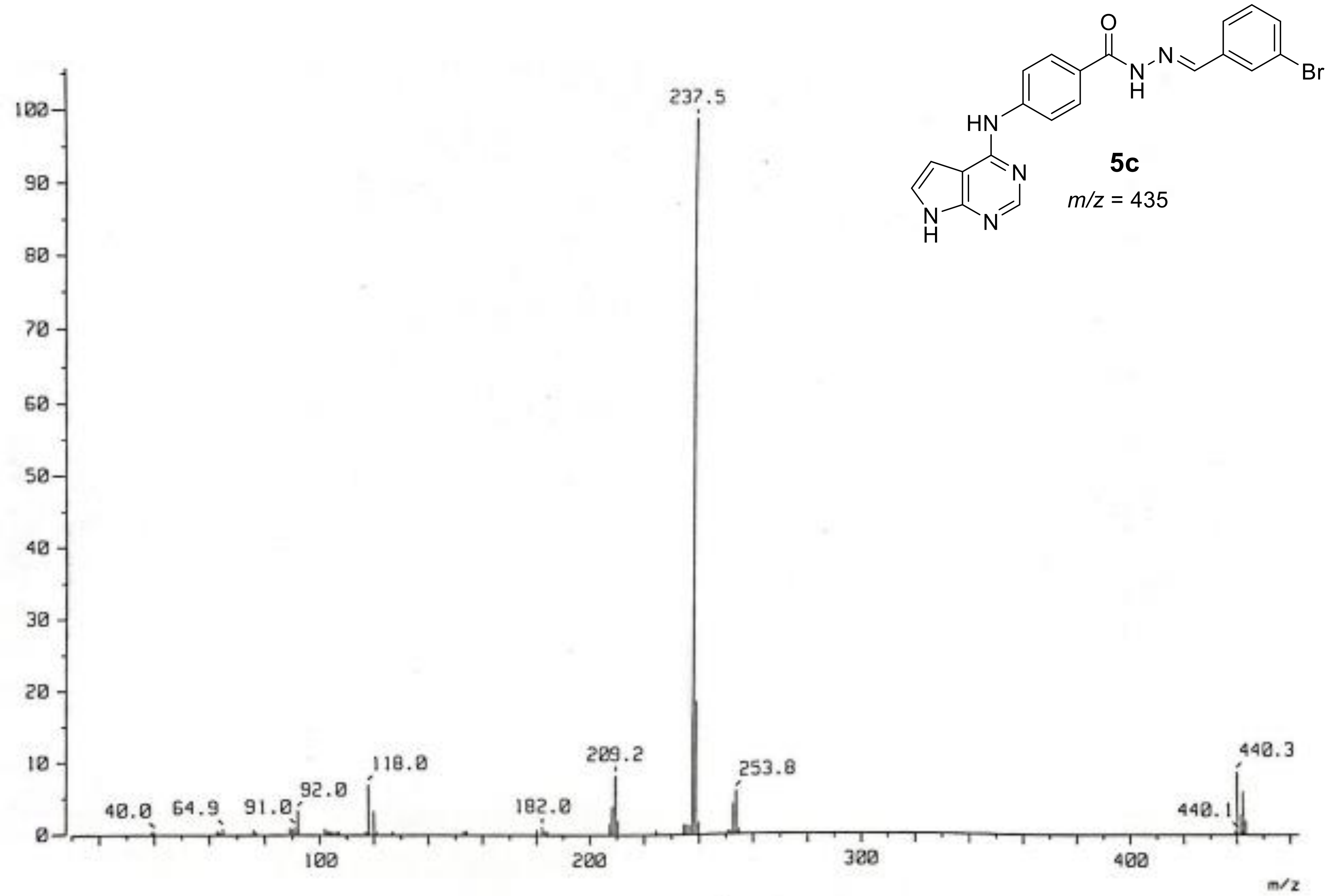

5c

m/z = 435

^{1}H-NMR spectra of **5d**

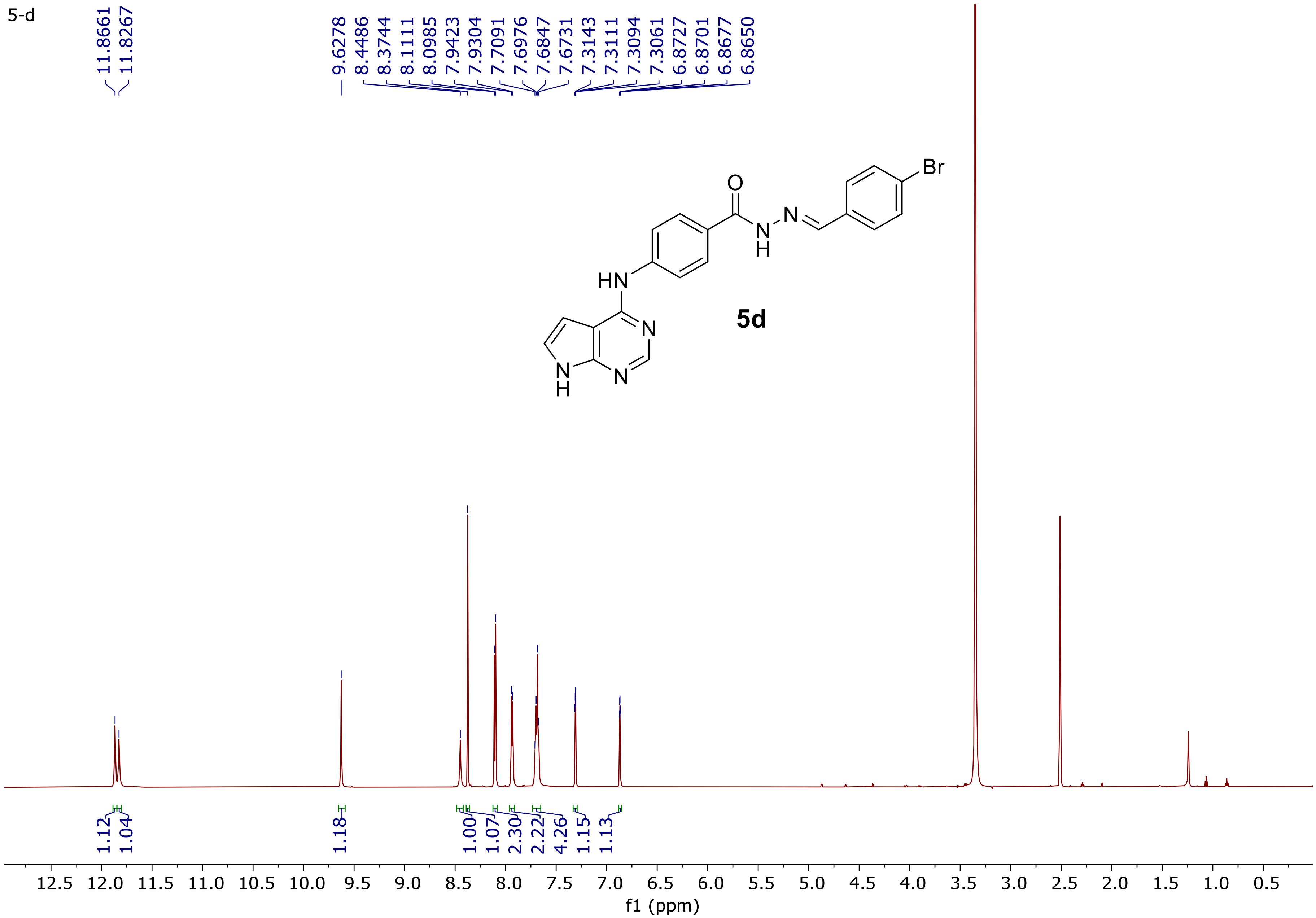

^{13}C-NMR spectra of **5d**

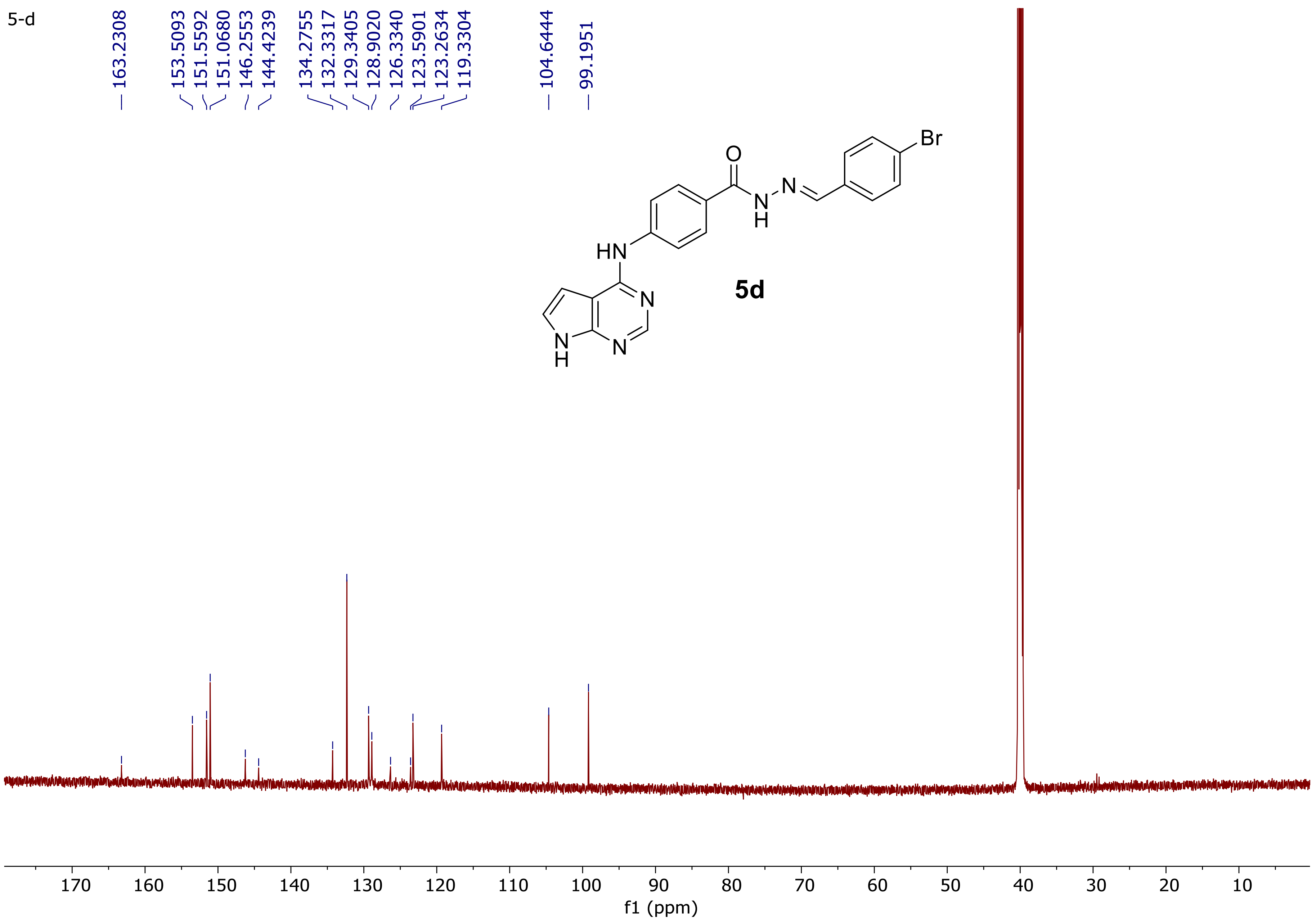

Mass spectra of **5d**

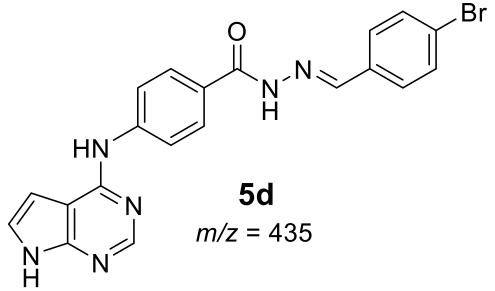

5d
m/z = 435

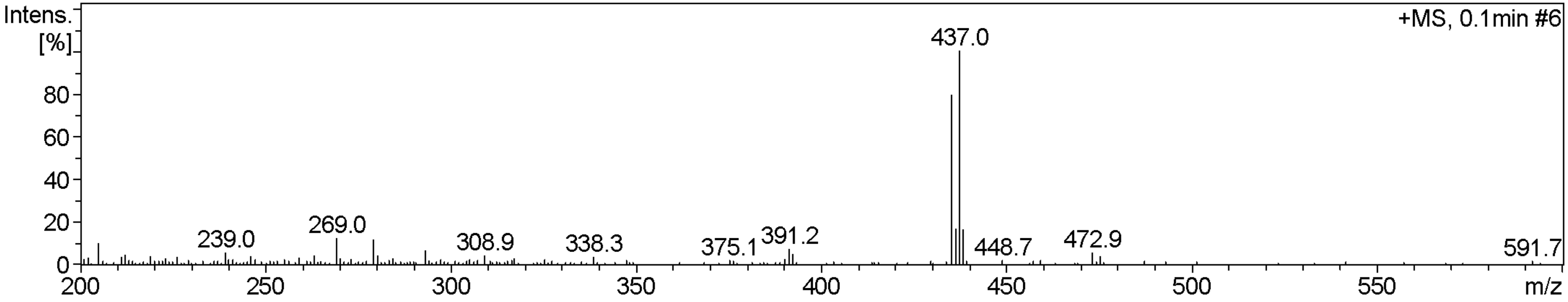

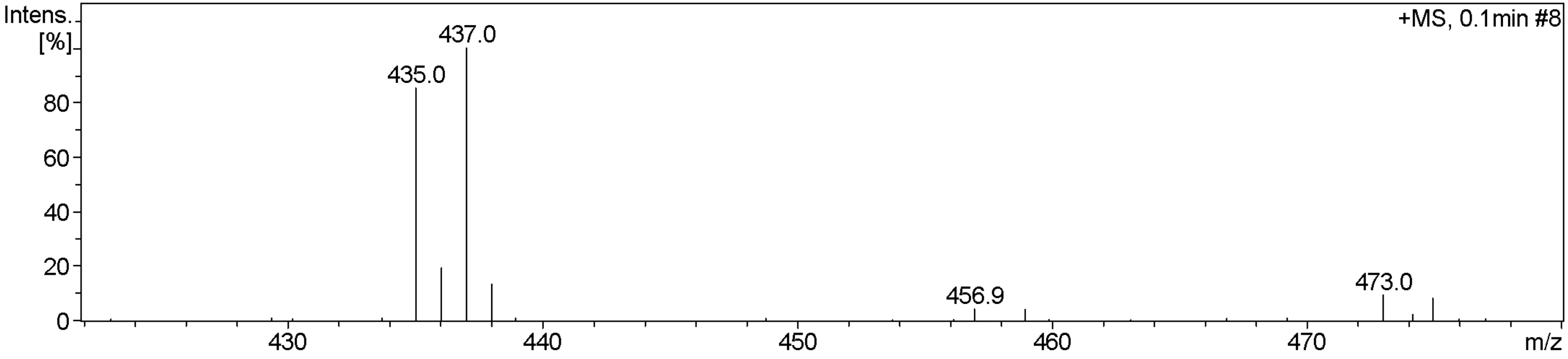

HMRS Mass spectra of **5d**

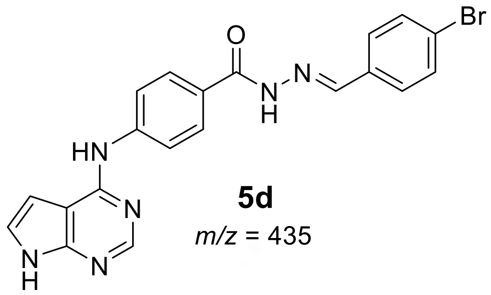

5d

m/z = 435

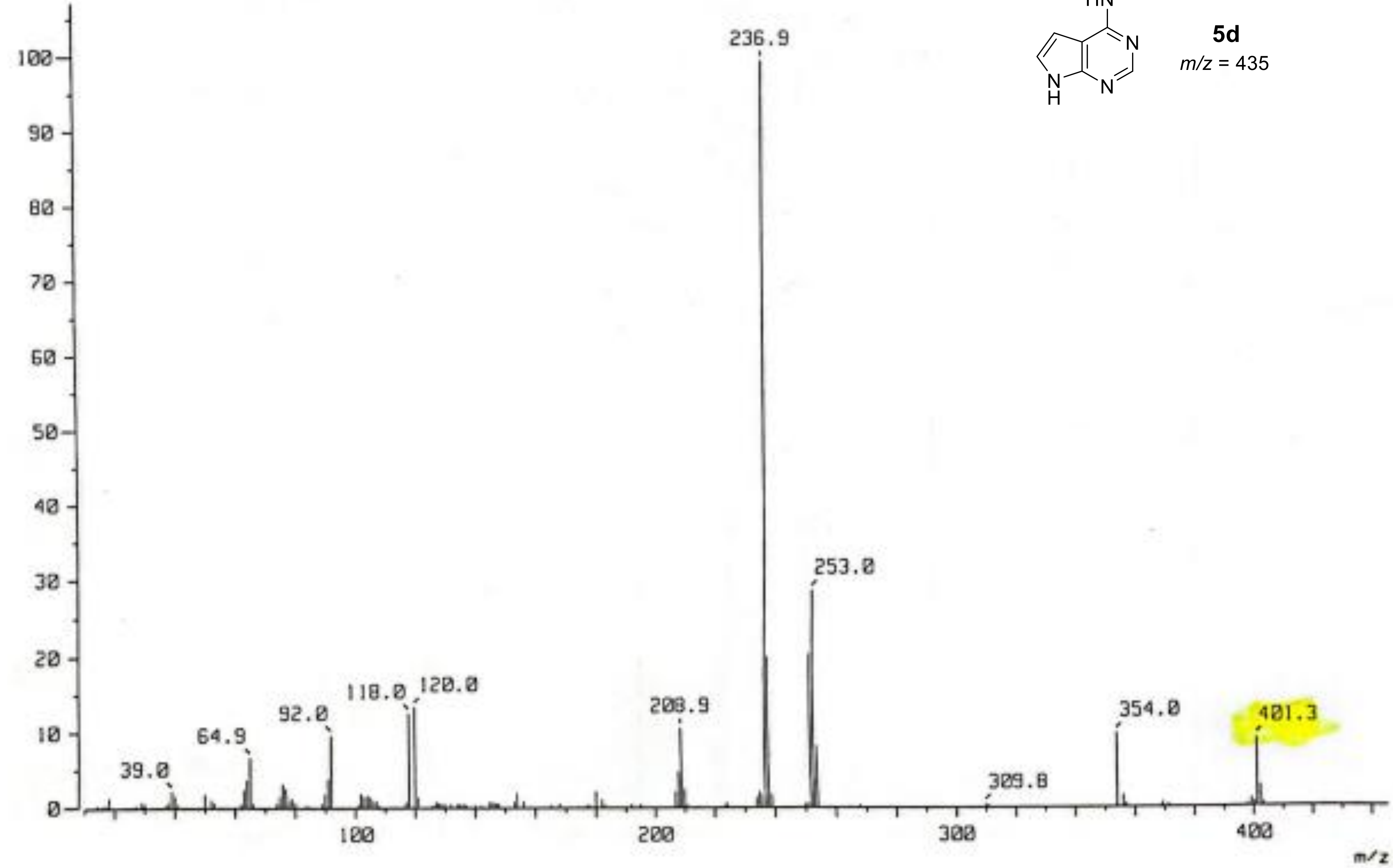

^{1}H-NMR spectra of **5e**

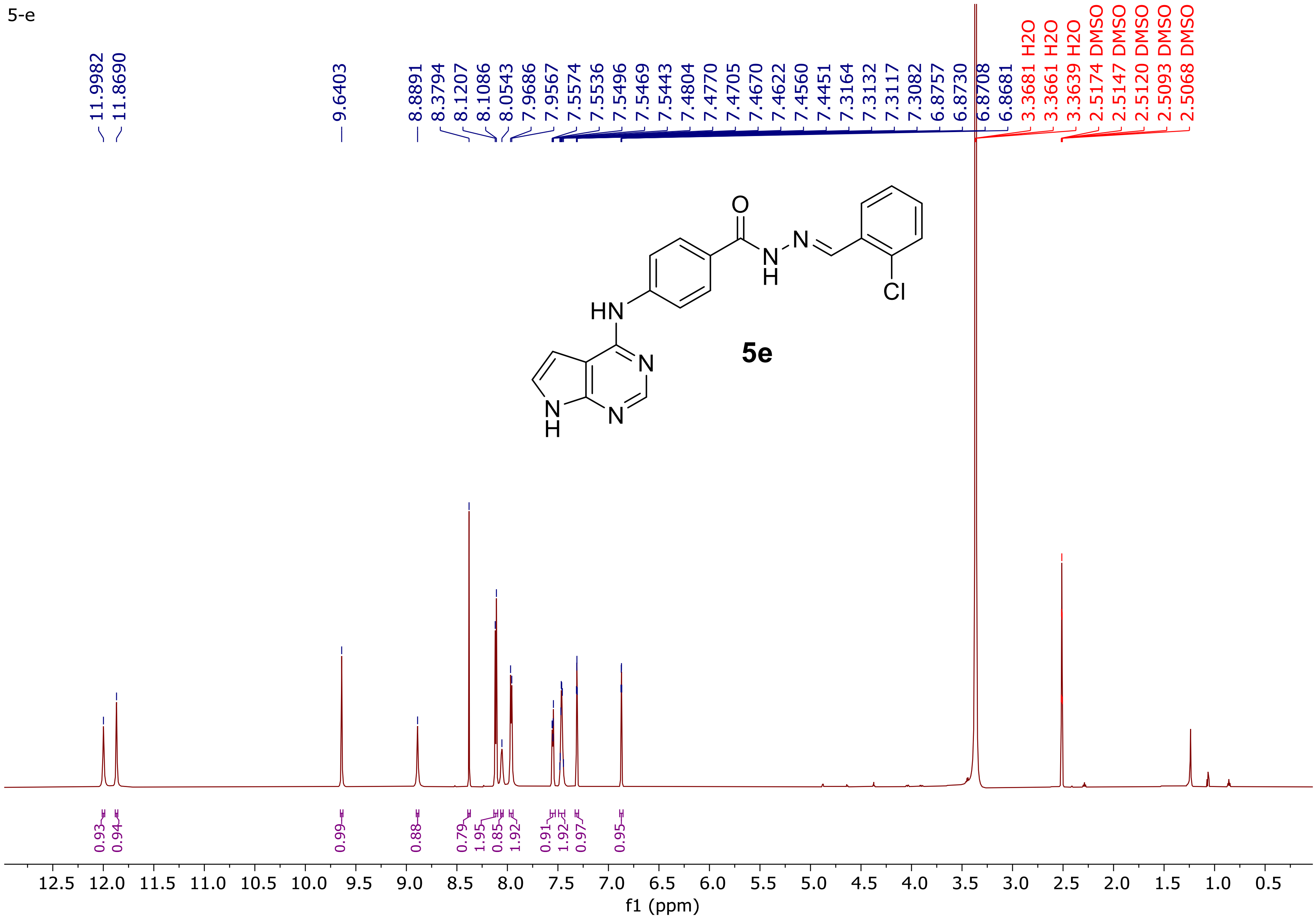

^{13}C-NMR spectra of **5e**

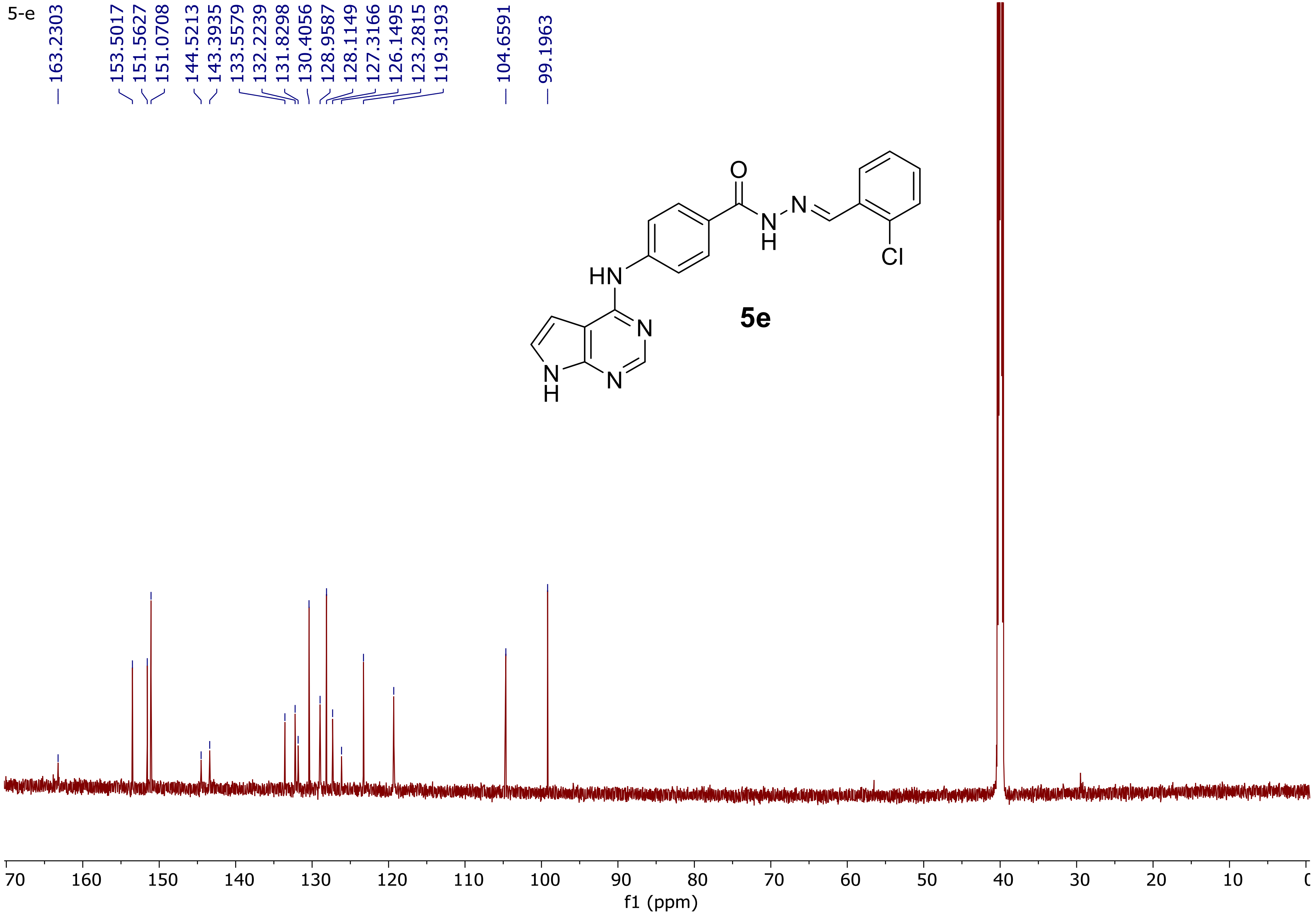

Mass spectra of **5e**

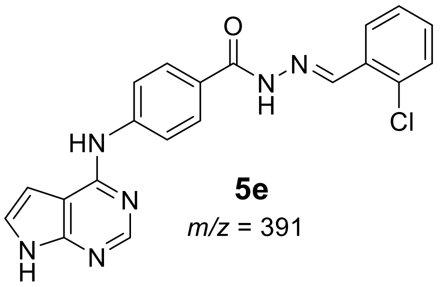

5e
m/z = 391

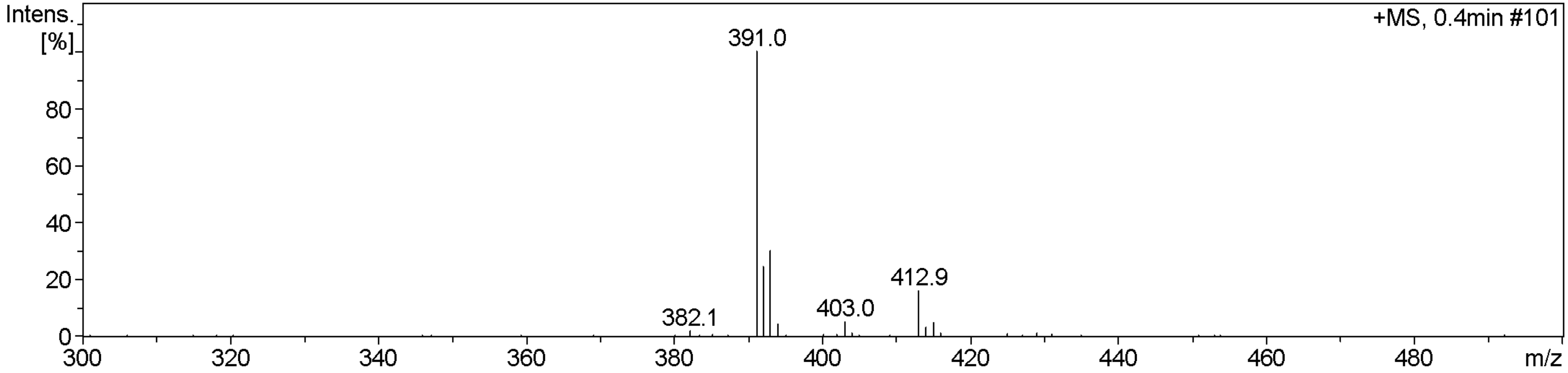

2, 3, 4,

HMRS Mass spectra of **5e**

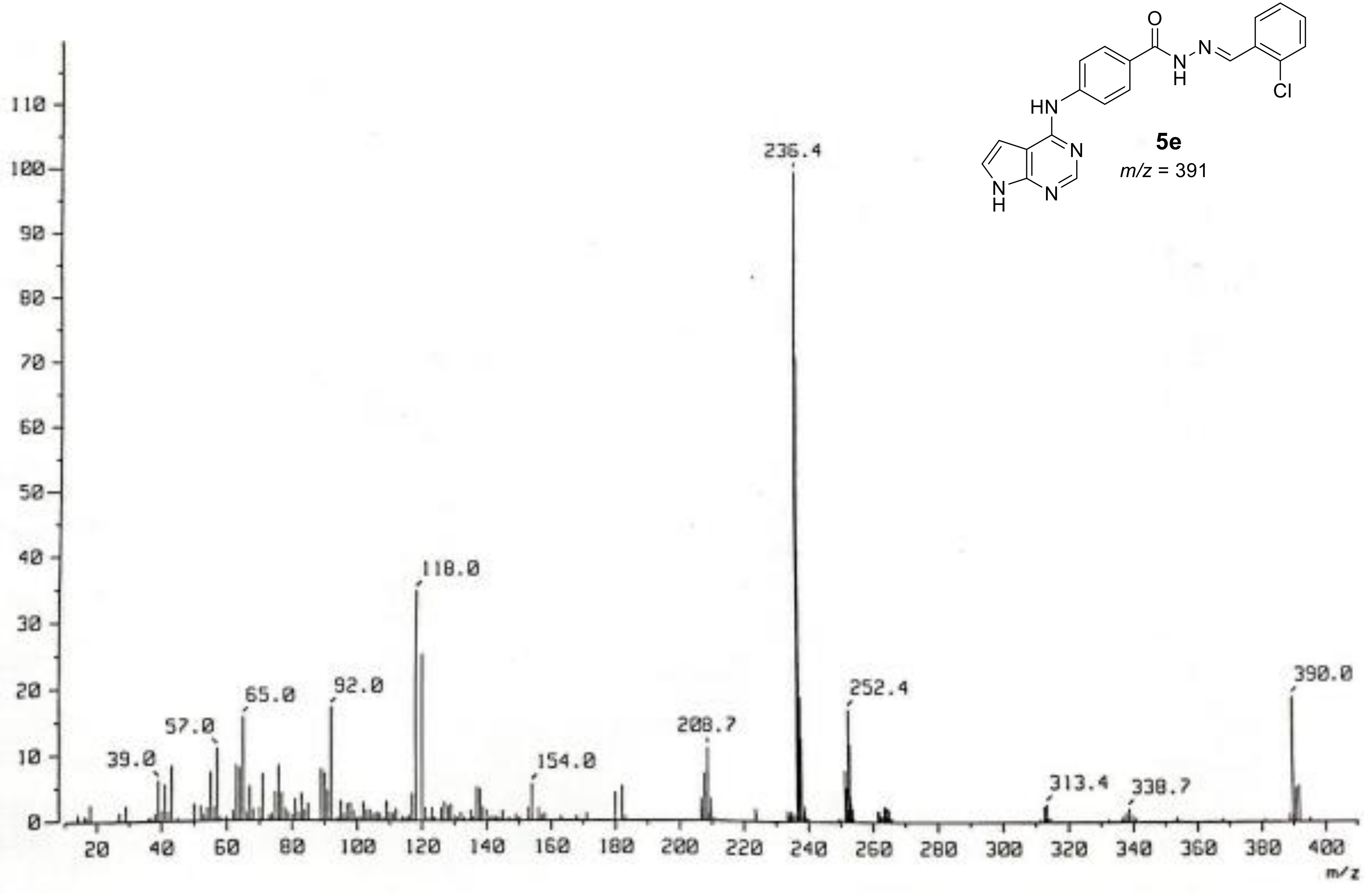

5e

m/z = 391

^{1}H-NMR spectra of **5f**

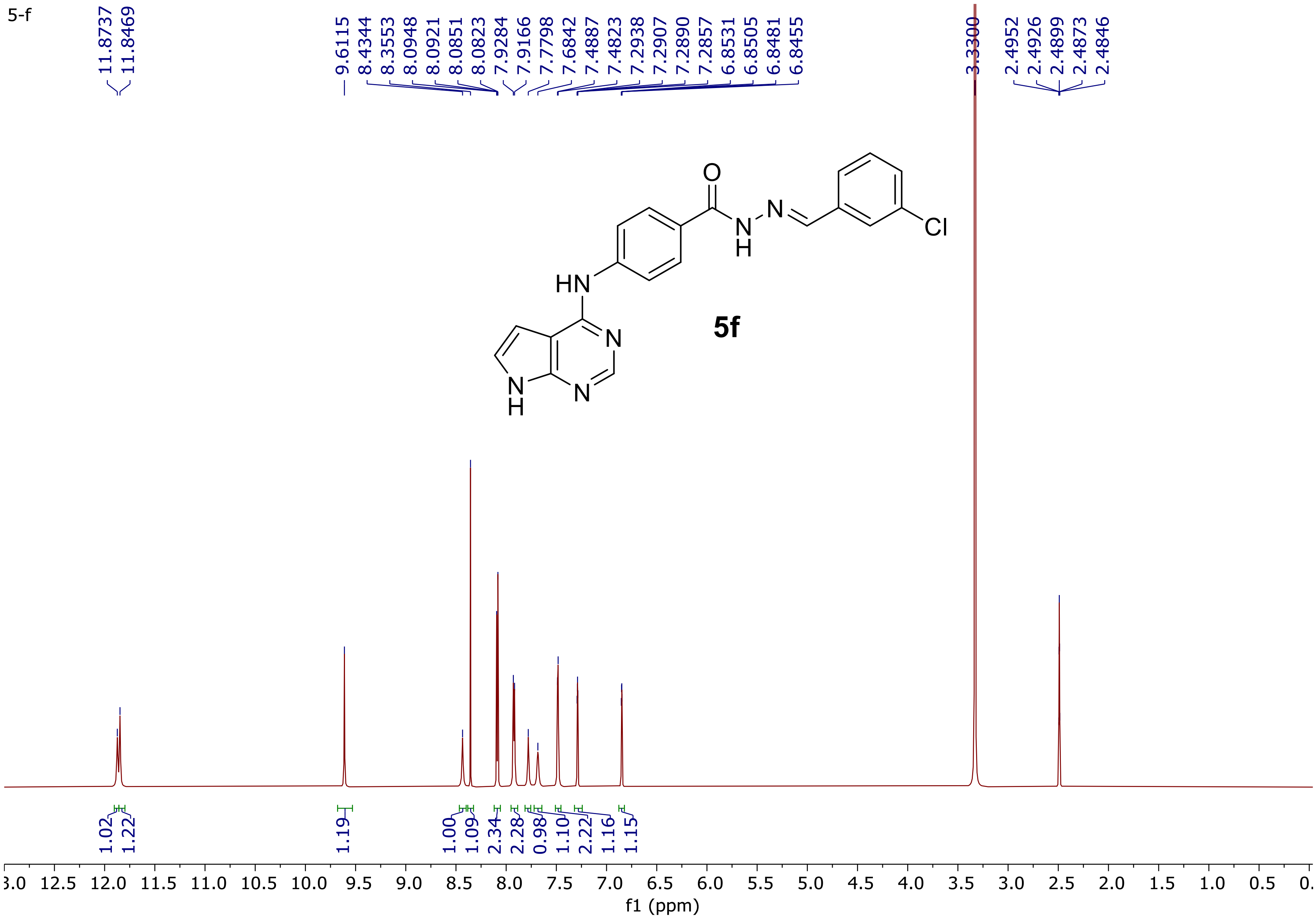

^{13}C-NMR spectra of **5f**

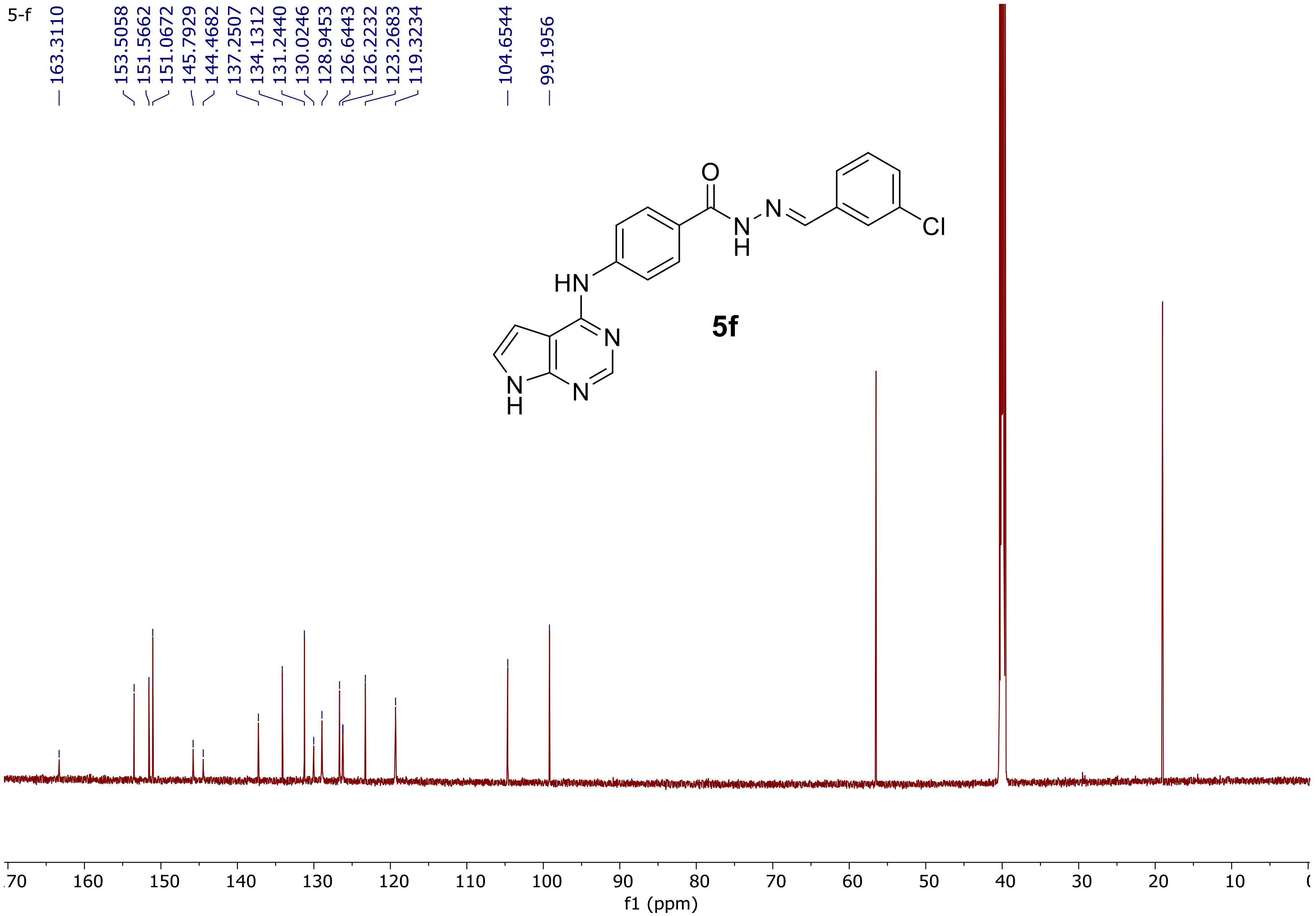

Mass spectra of **5f**

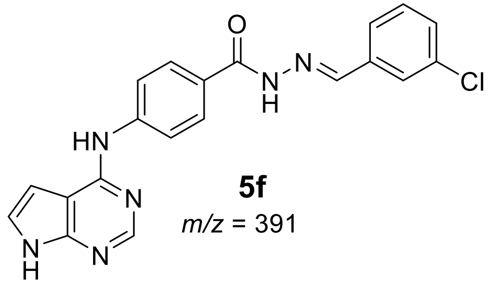

5f
m/z = 391

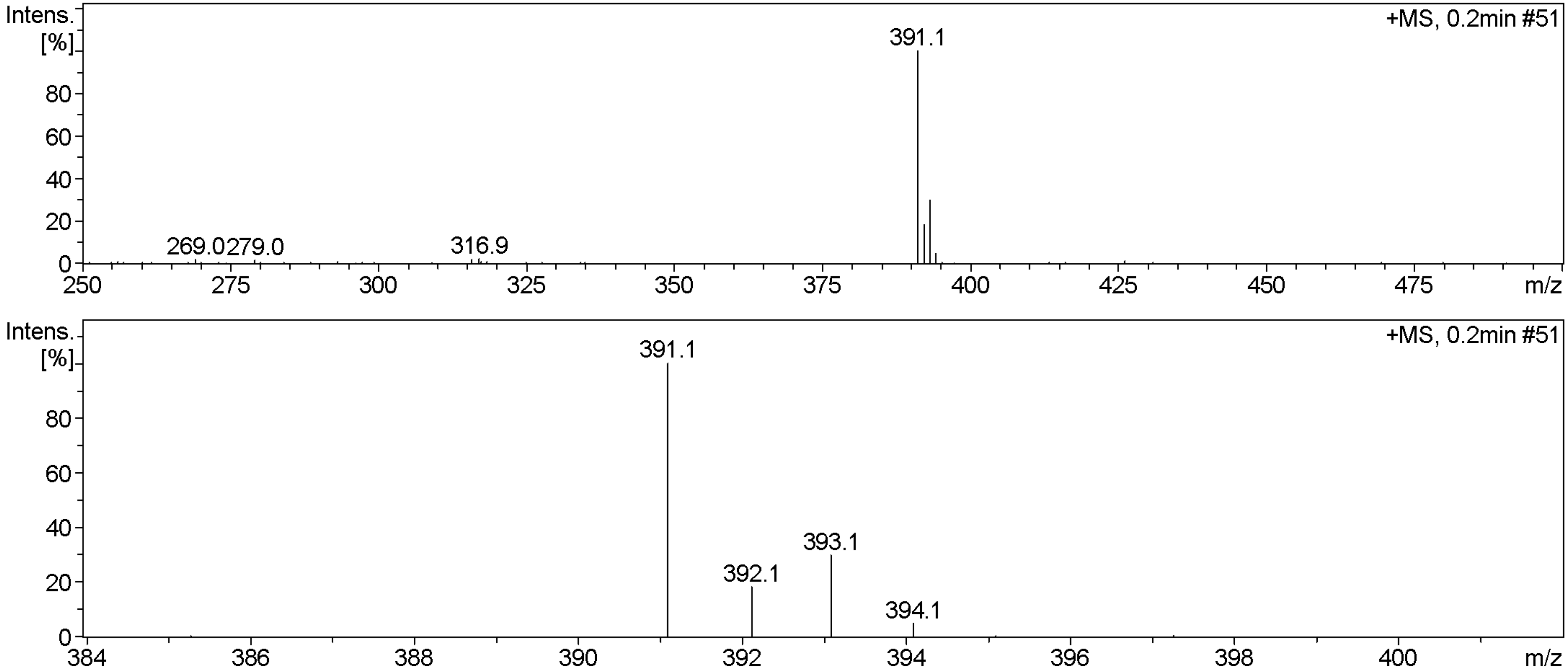

HMRS Mass spectra of **5f**

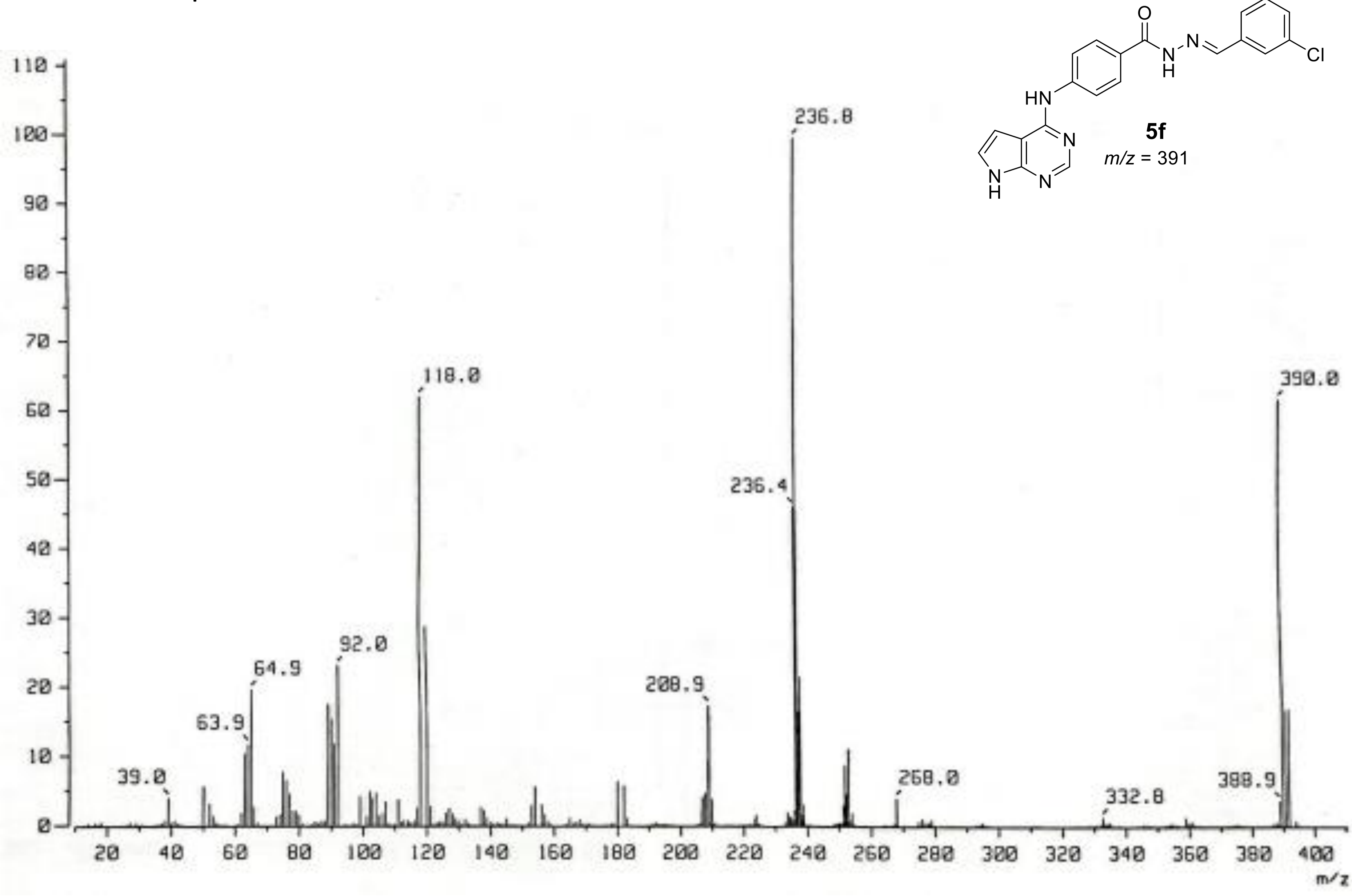

5f
m/z = 391

^{1}H-NMR spectra of **5g**

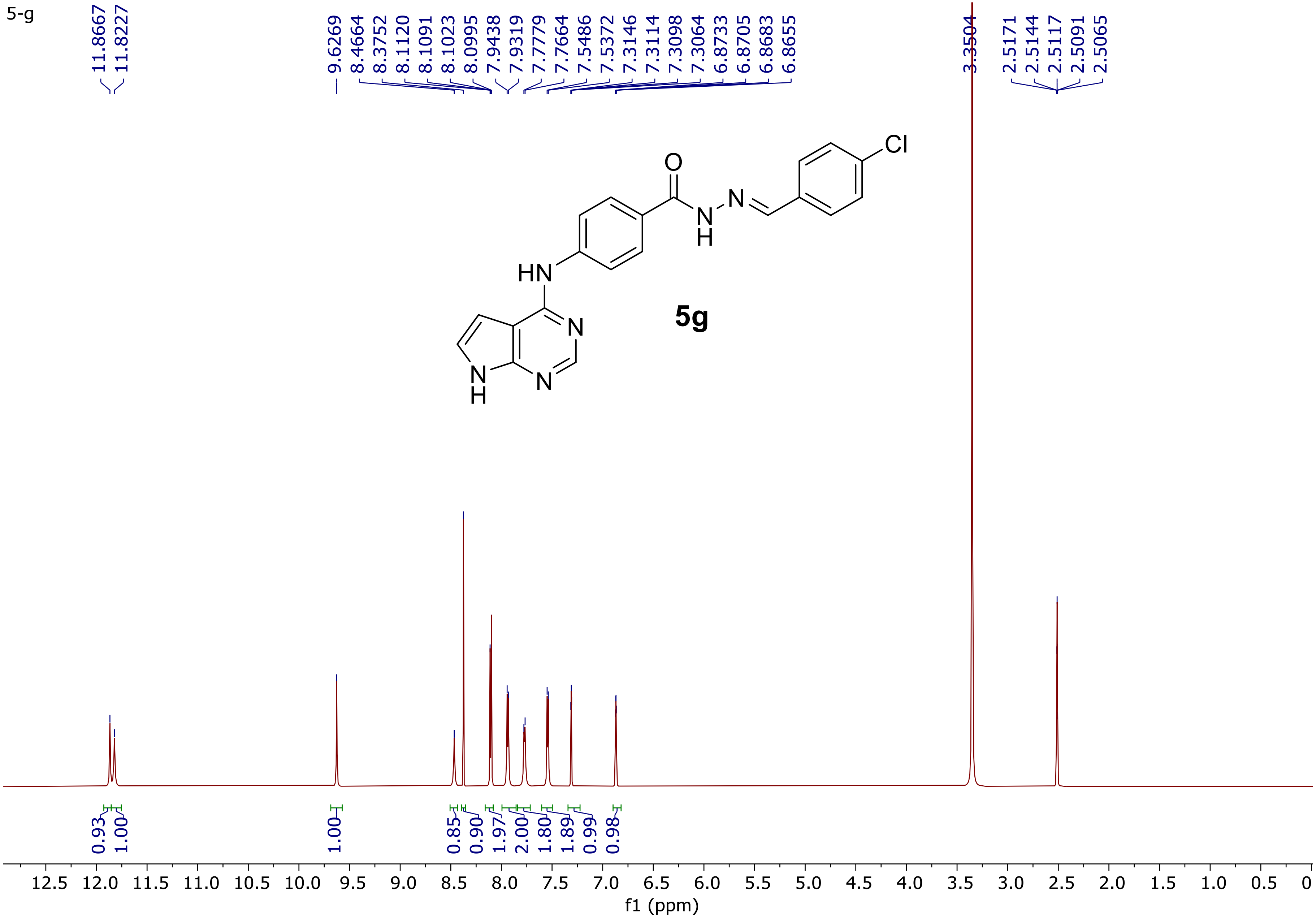

^{13}C-NMR spectra of **5g**

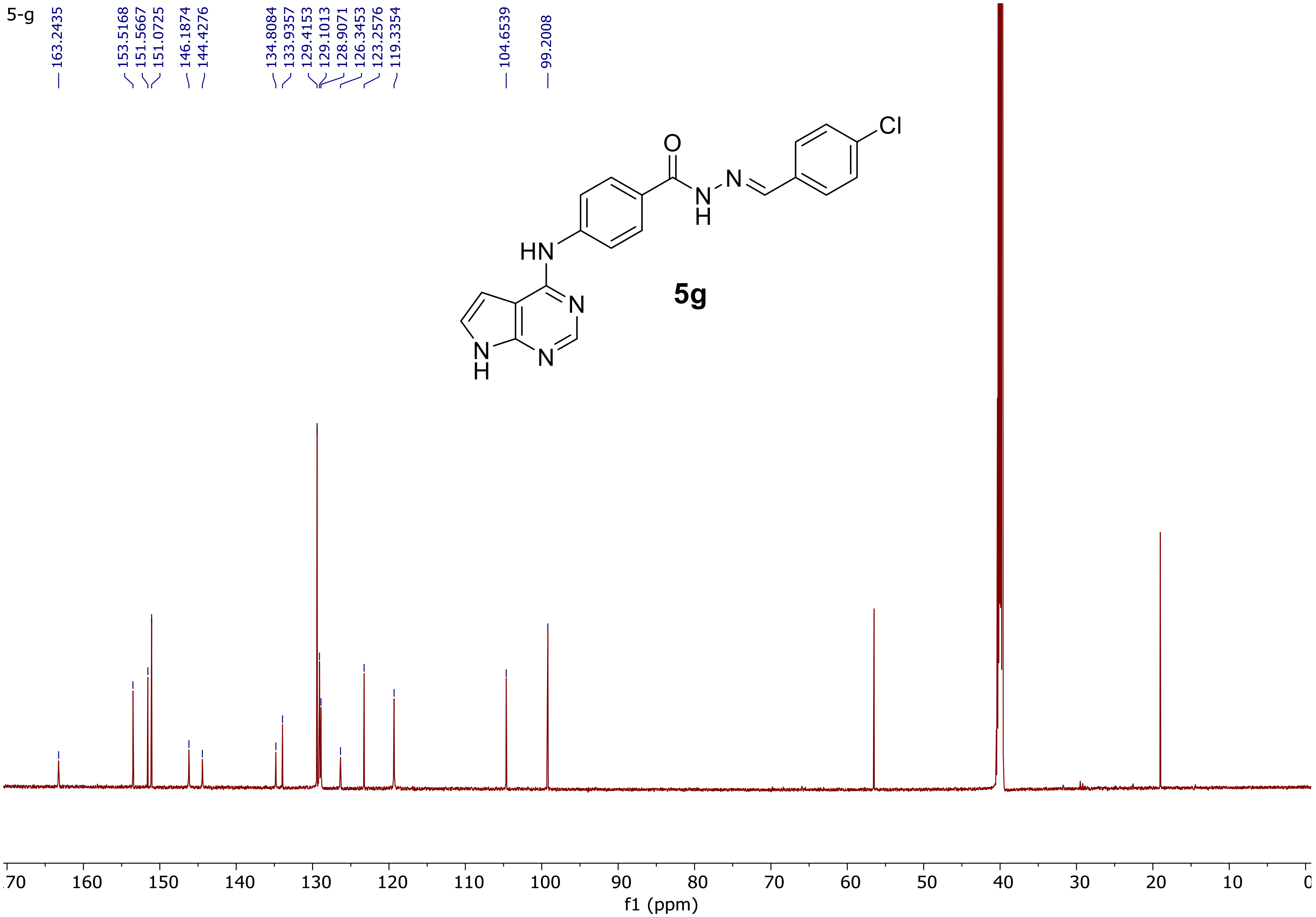

Mass spectra of **5g**

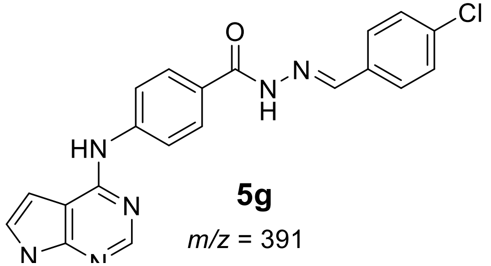

5g
m/z = 391

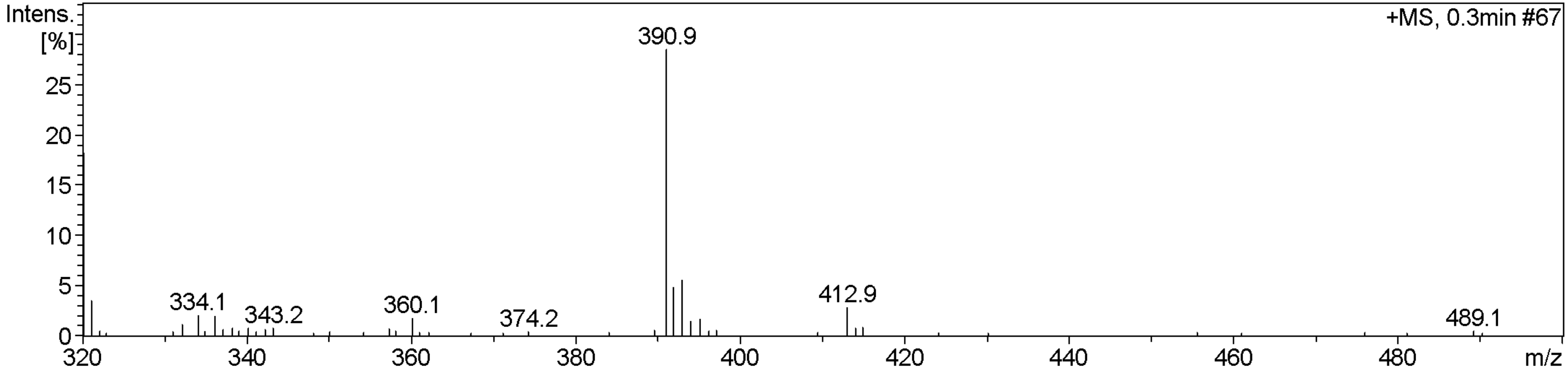

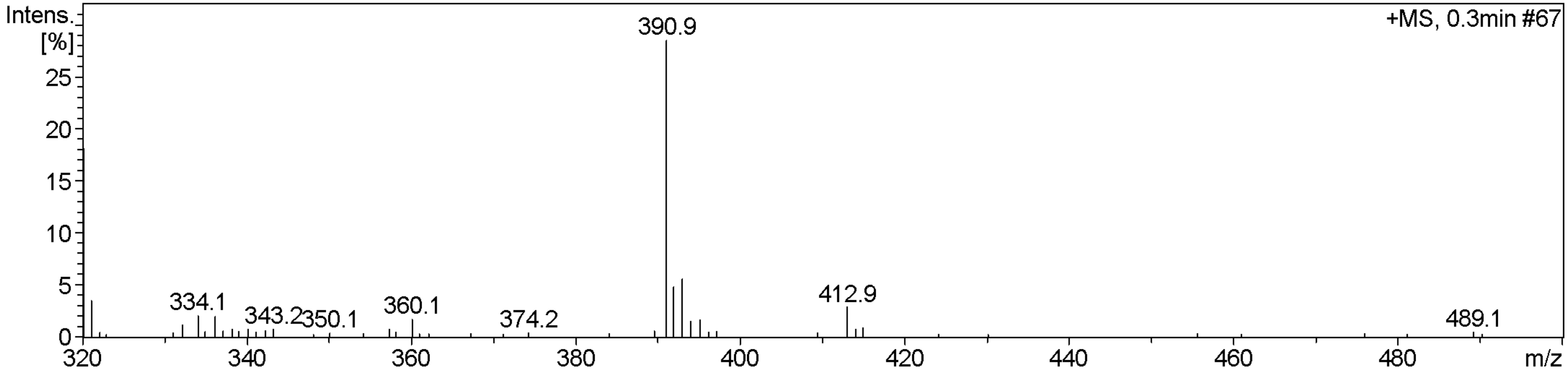

HMRS Mass spectra of **5g**

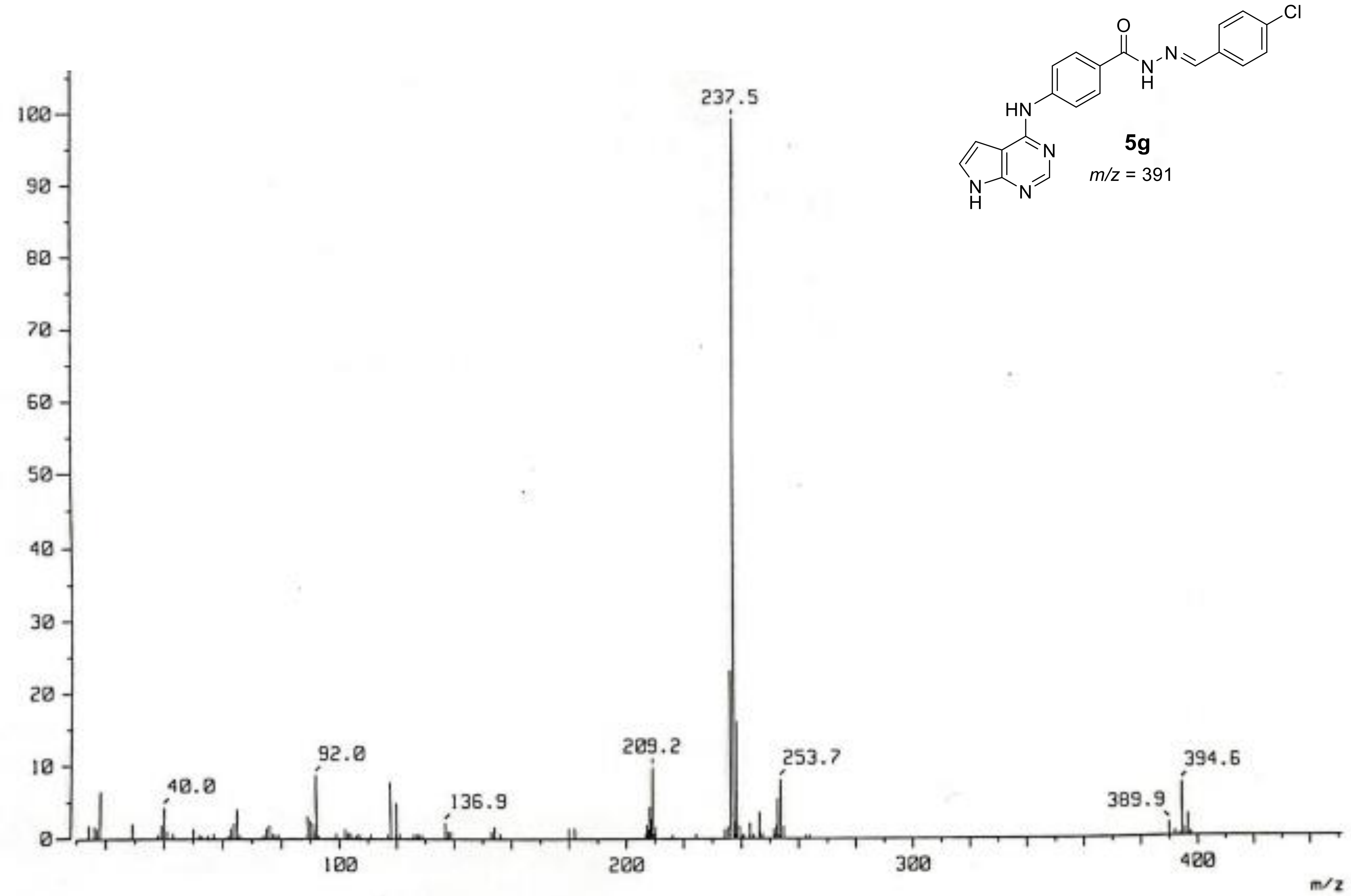

^{1}H-NMR spectra of **5h**

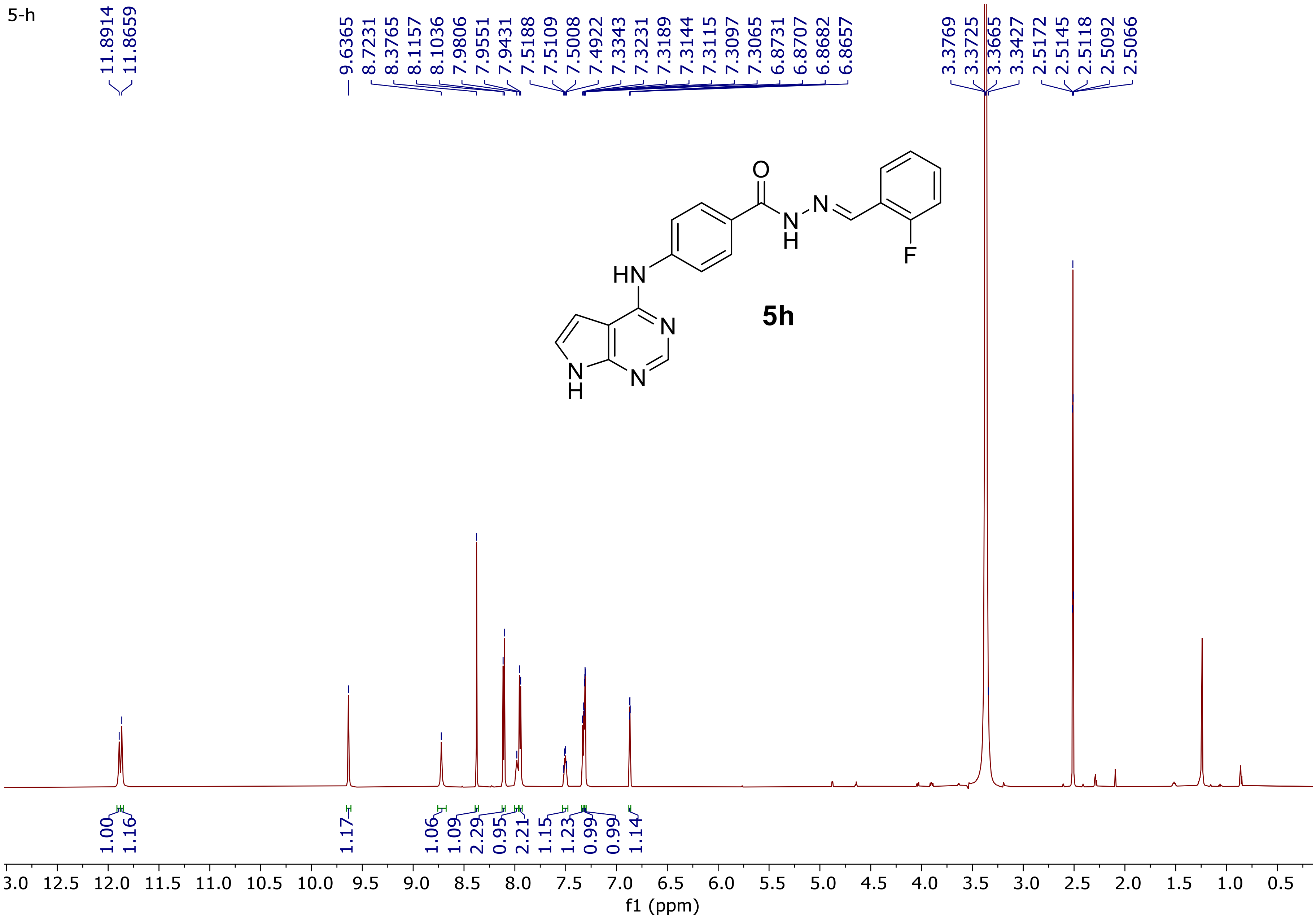

^{13}C-NMR spectra of **5h**

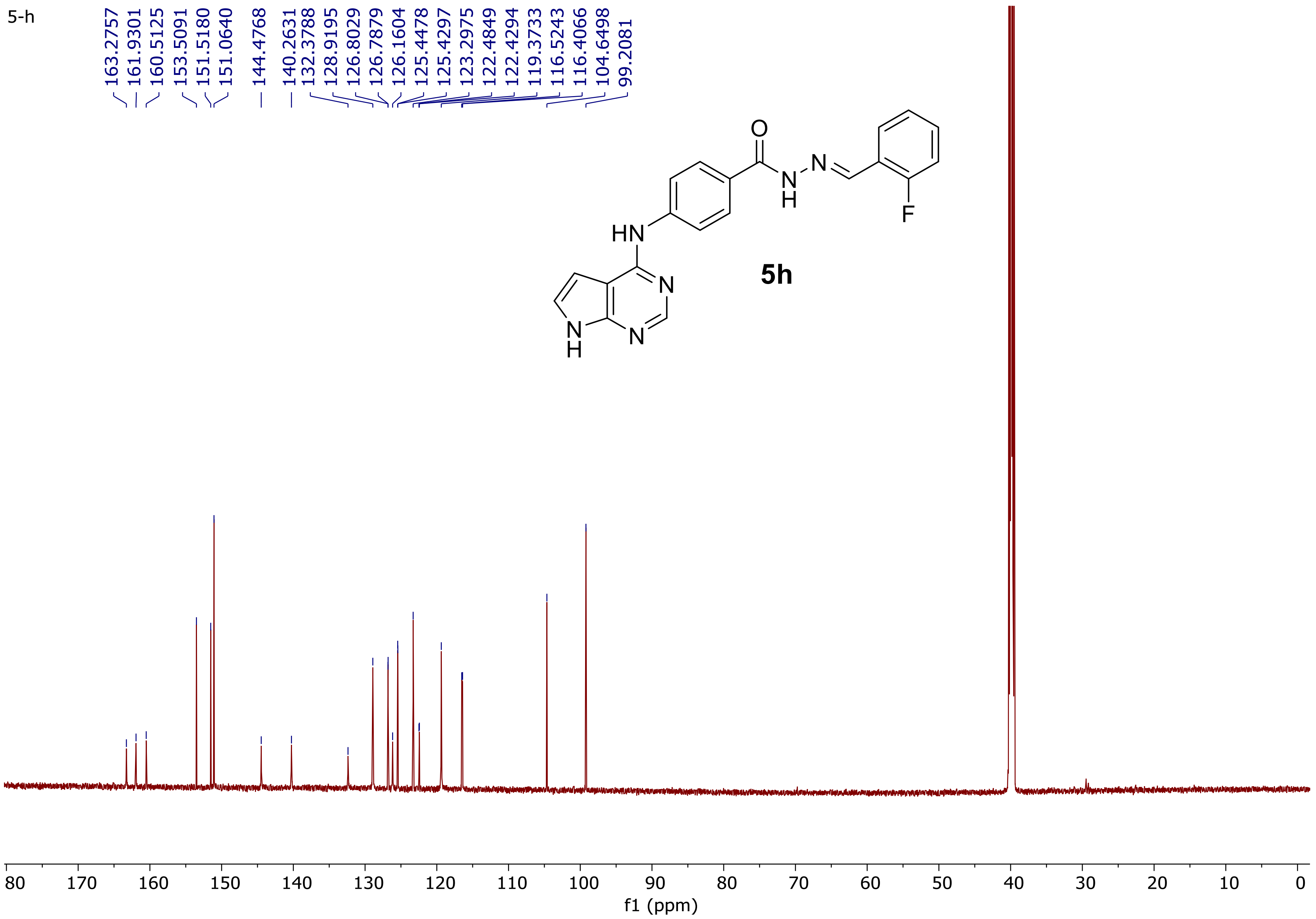

Mass spectra of **5h**

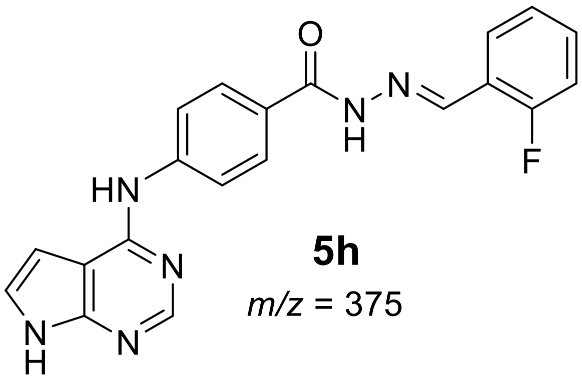

5h
m/z = 375

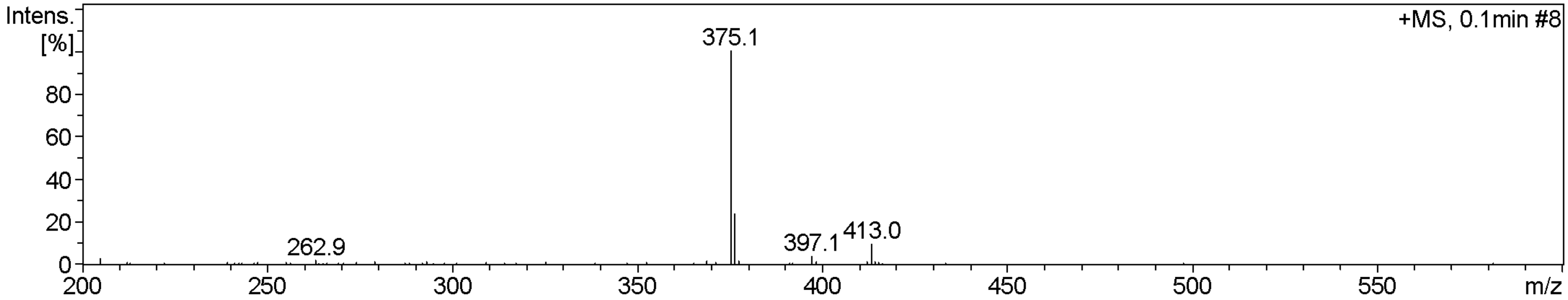

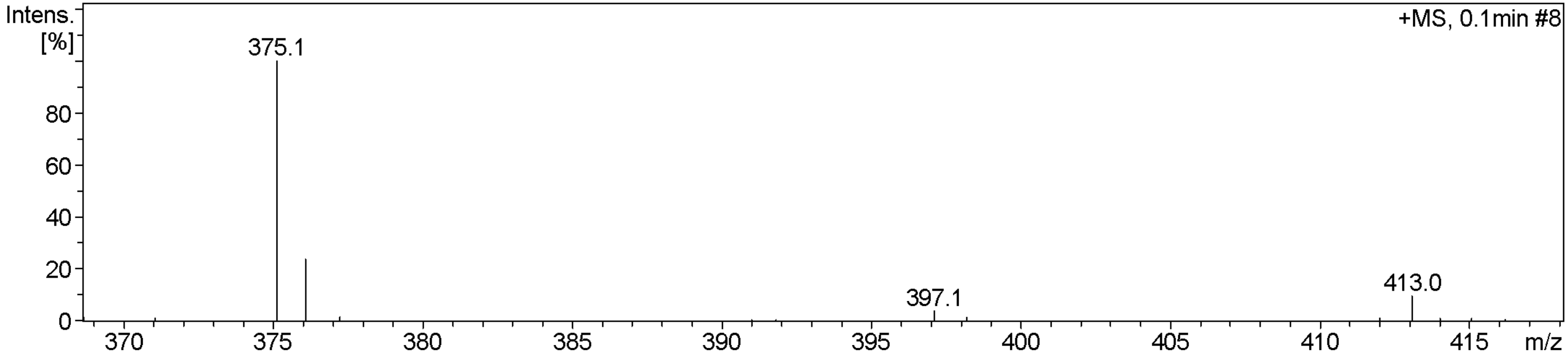

HMRS Mass spectra of **5h**

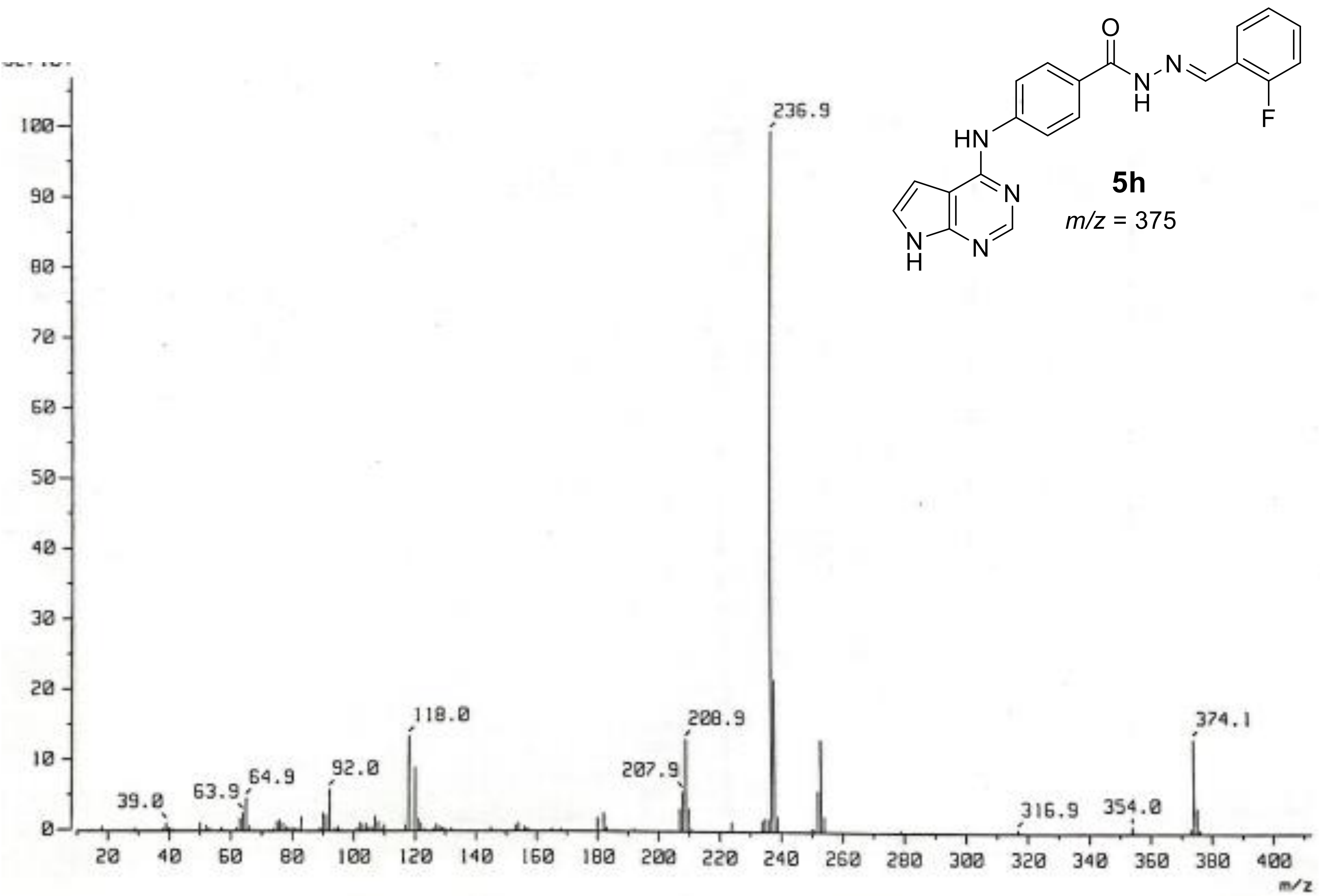

5h

m/z = 375

^{1}H-NMR spectra of **5i**

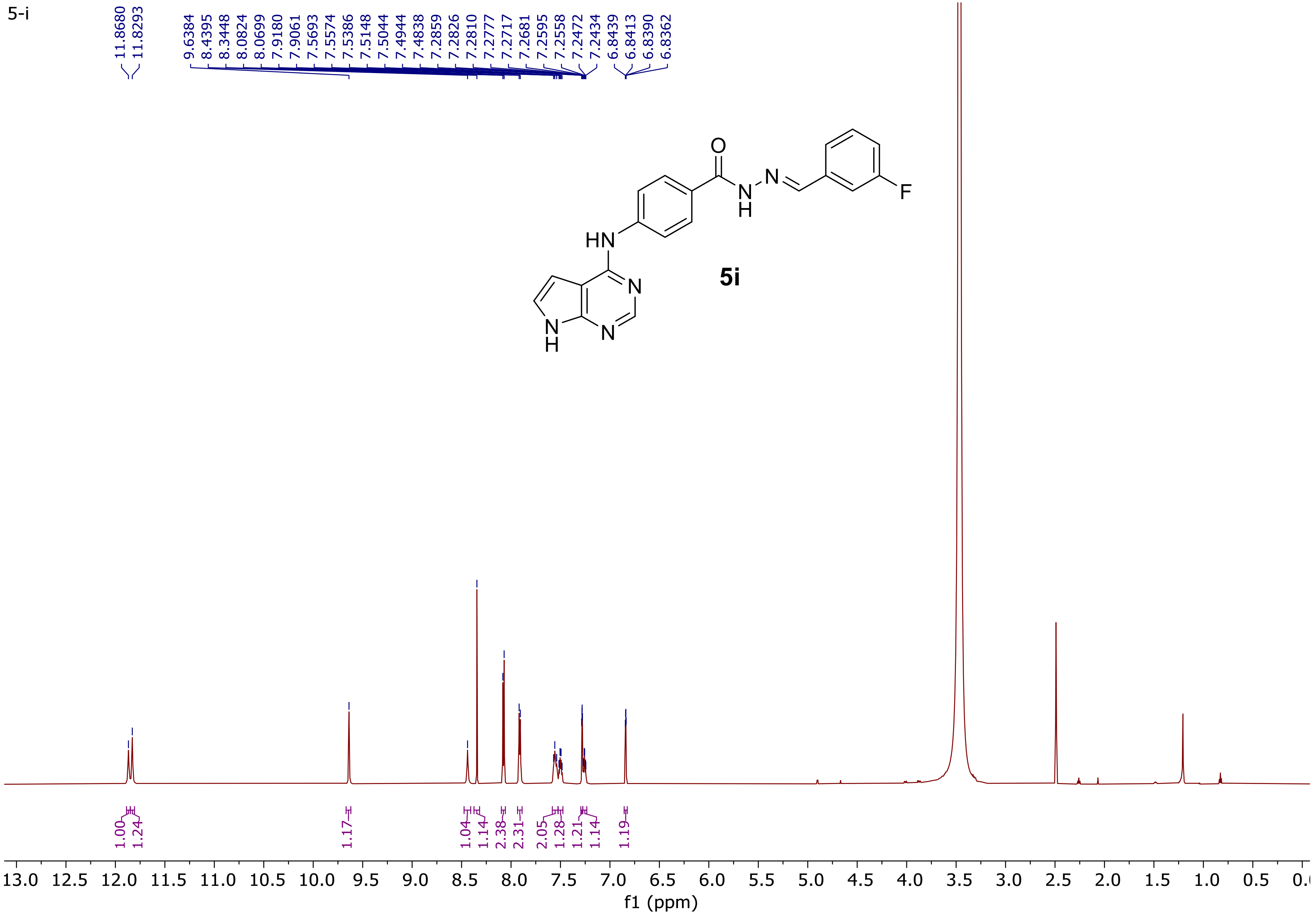

^{13}C-NMR spectra of **5i**

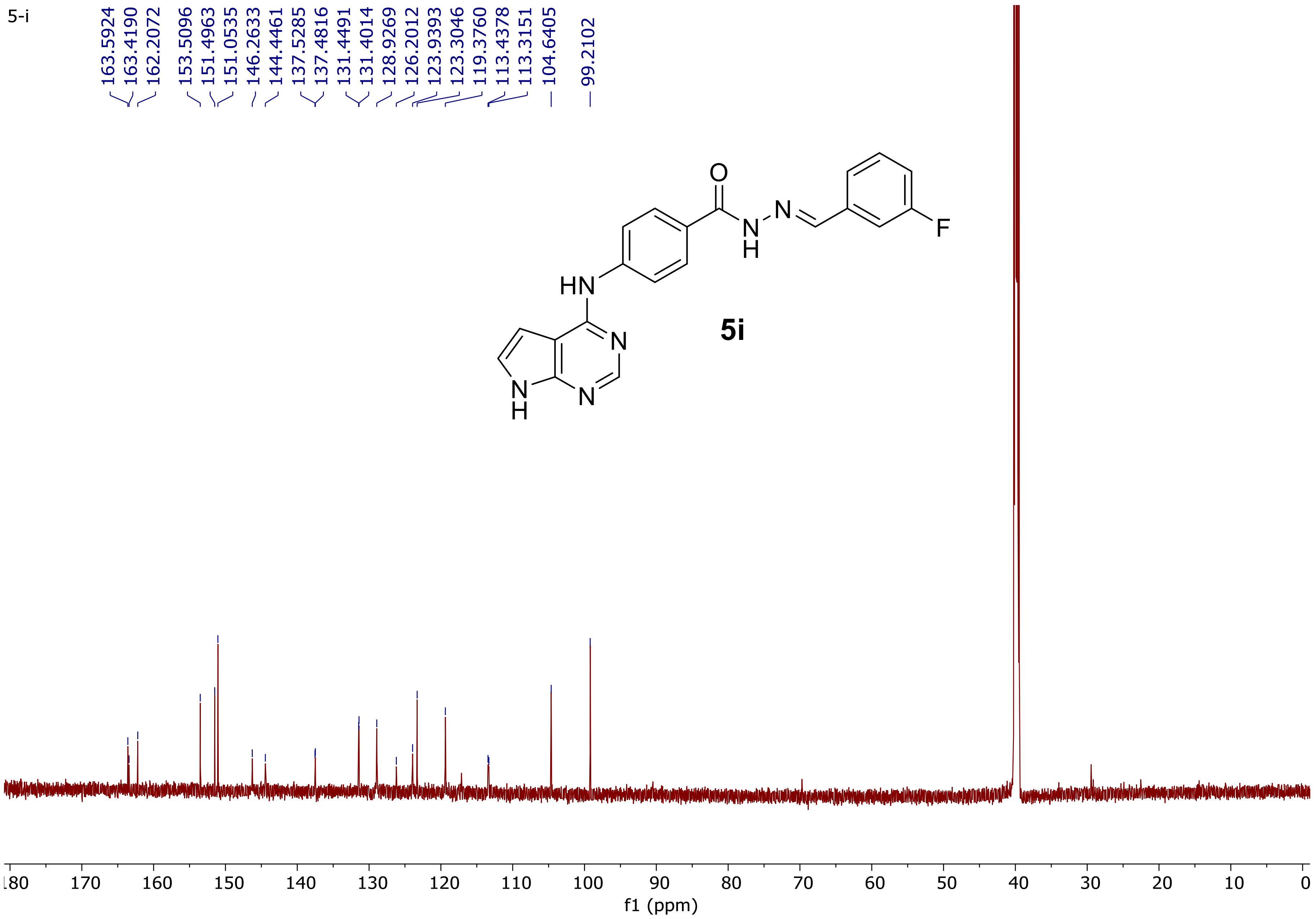

Mass spectra of **5i**

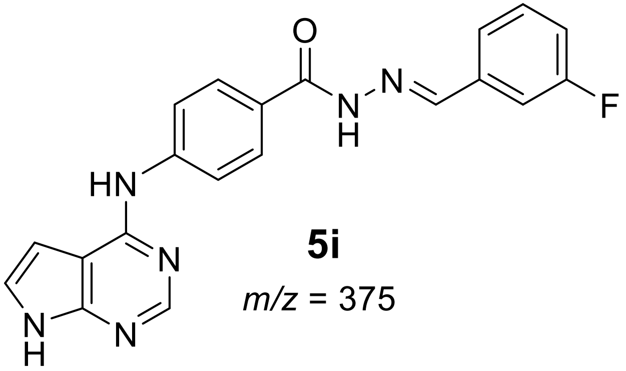

5i

m/z = 375

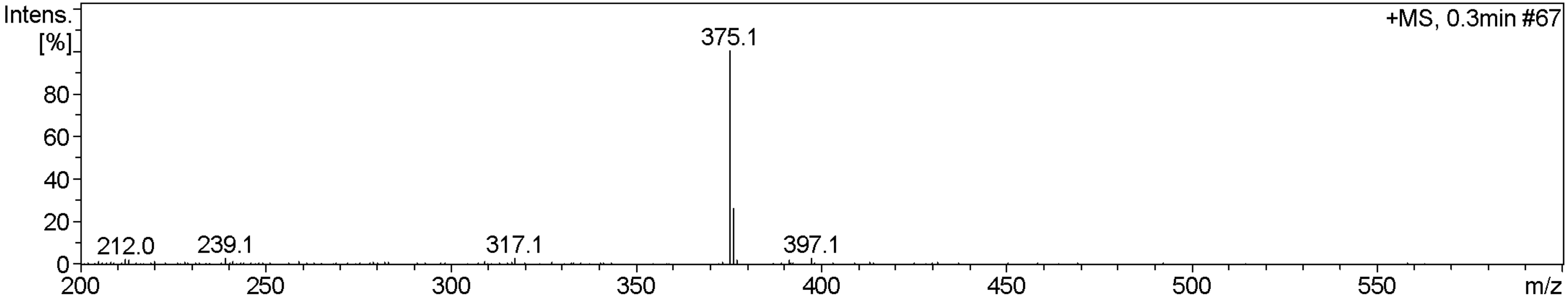

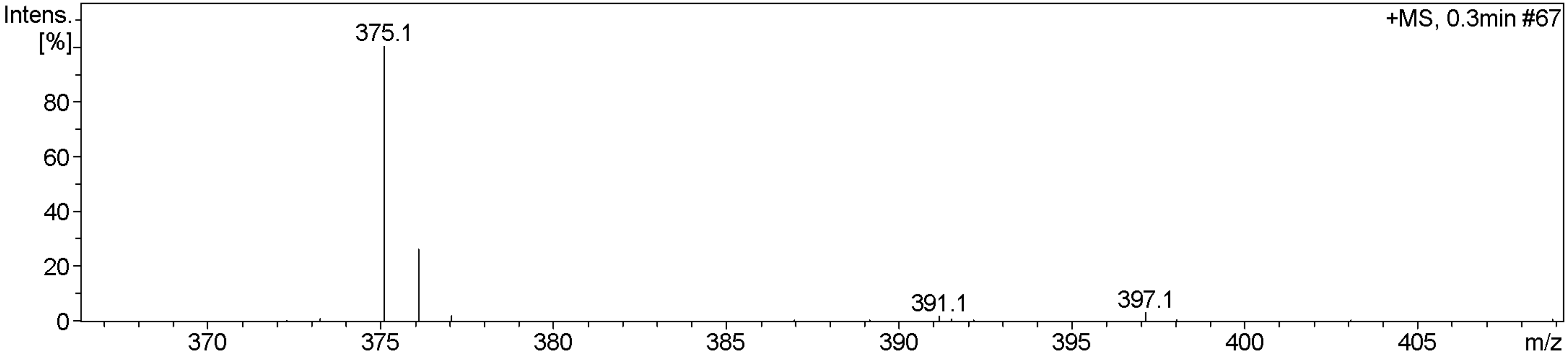

HMRS Mass spectra of **5i**

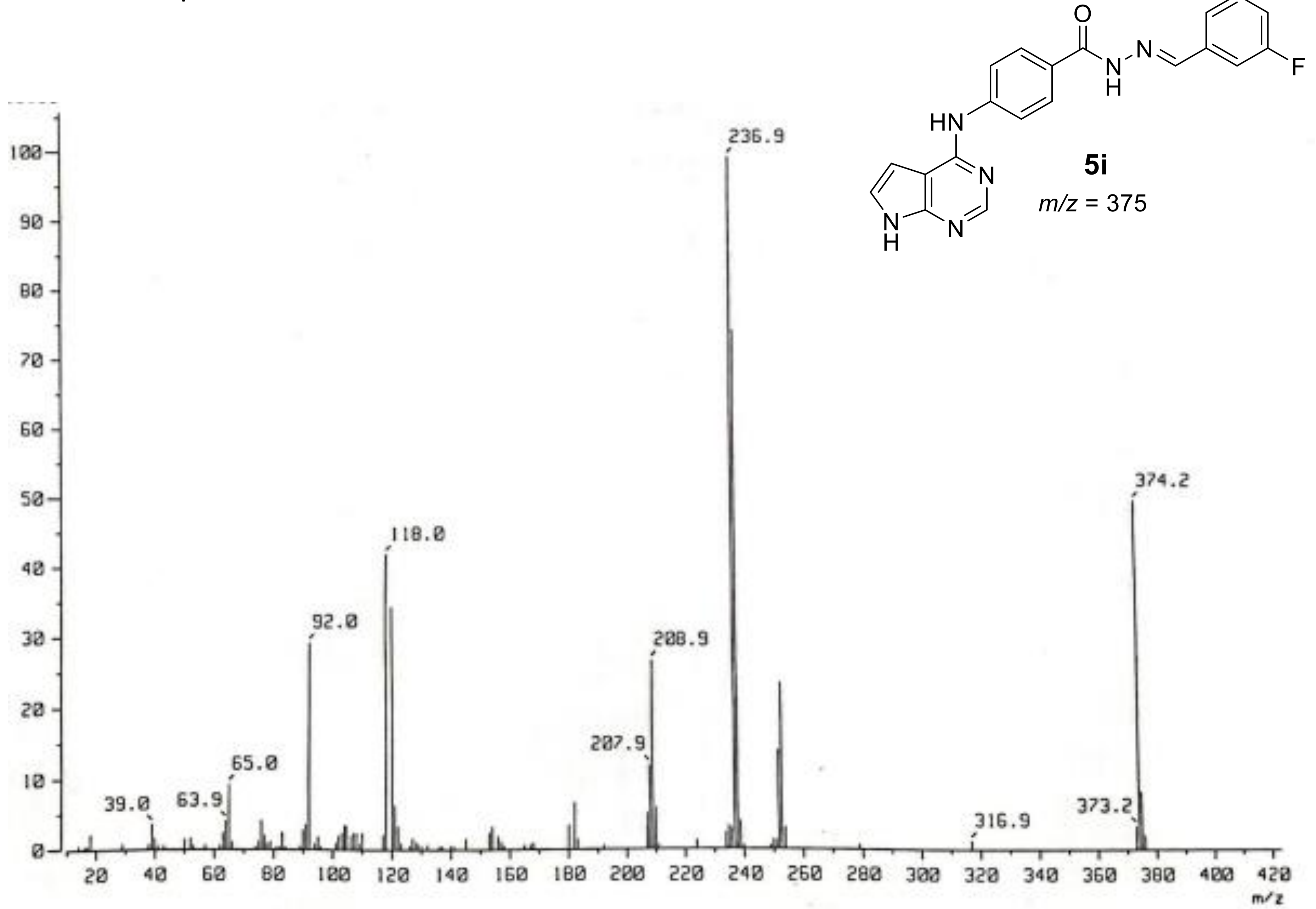

^{1}H-NMR spectra of **5j**

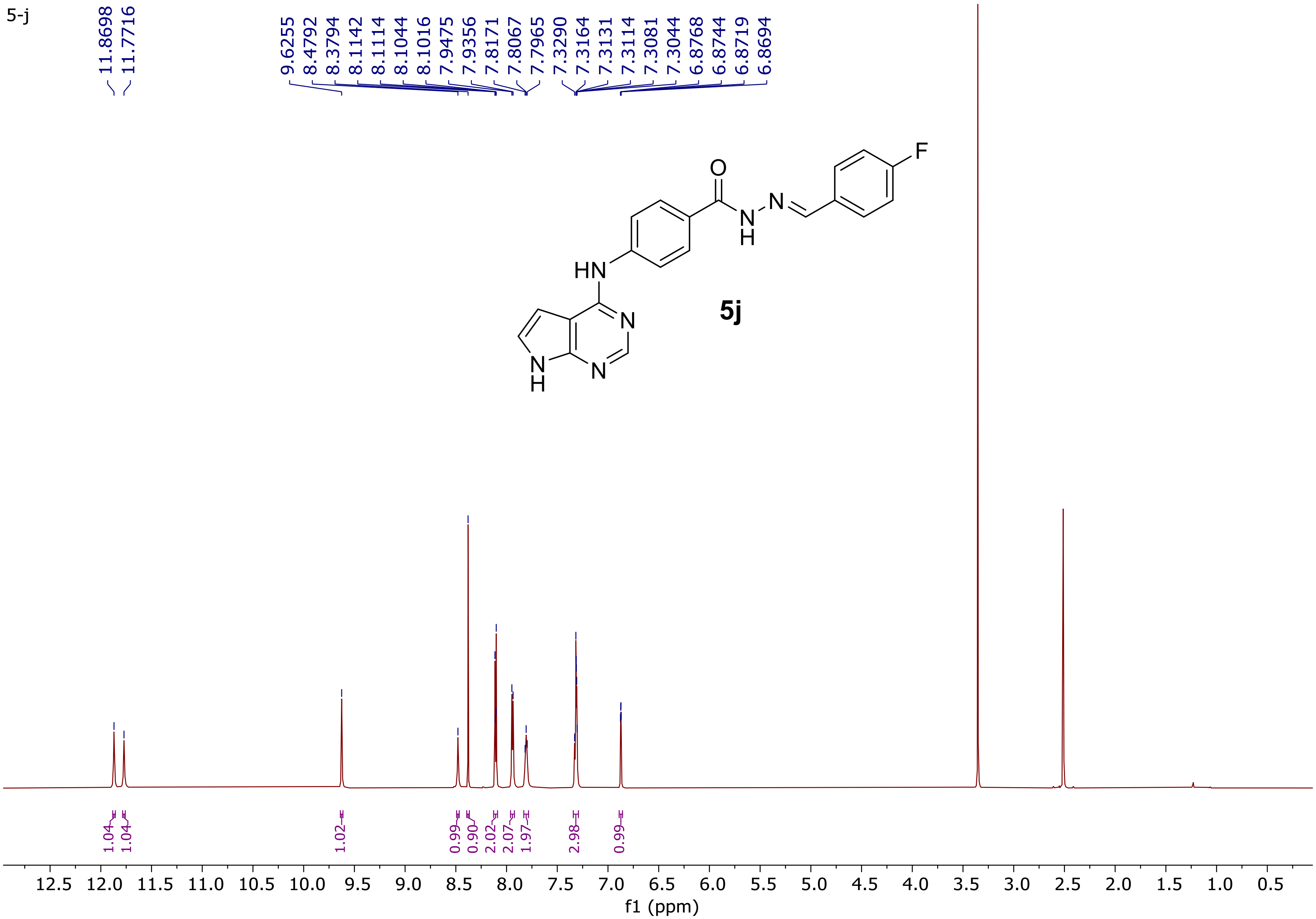

^{13}C-NMR spectra of **5j**

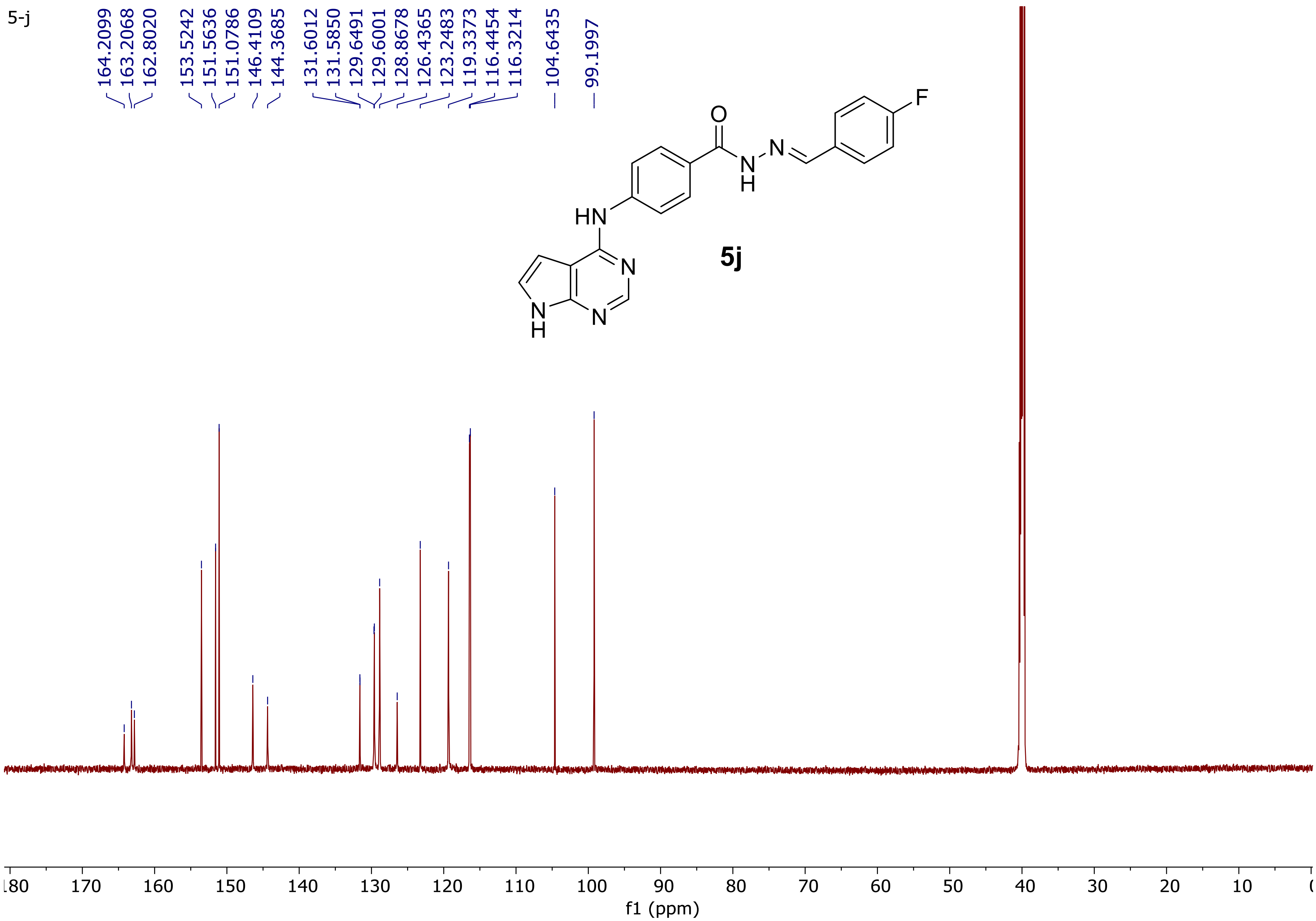

Mass spectra of **5j**

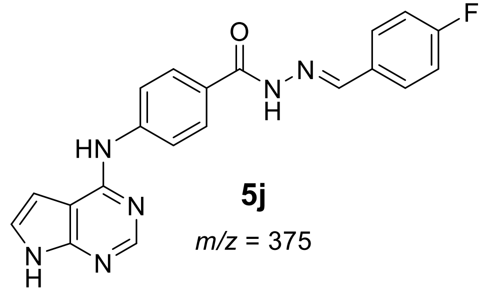

5j

m/z = 375

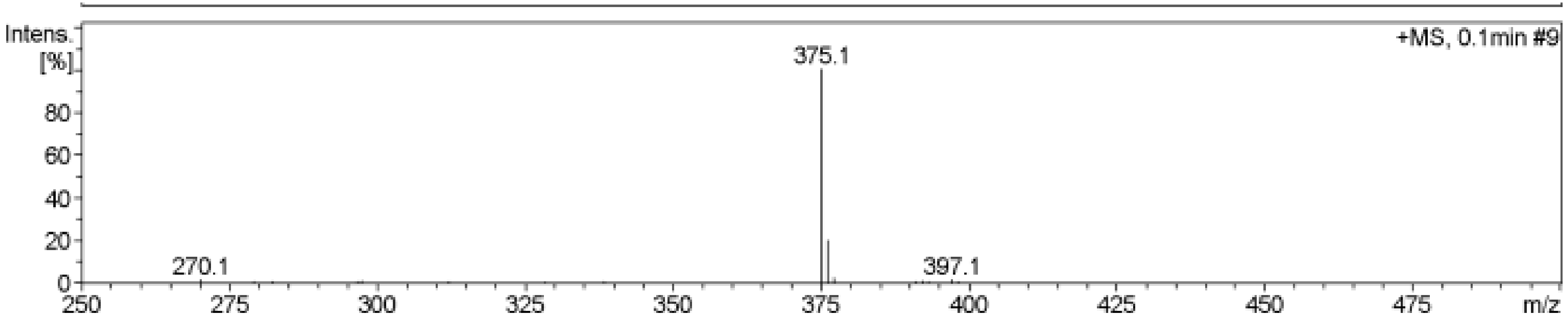

HMRS Mass spectra of **5j**

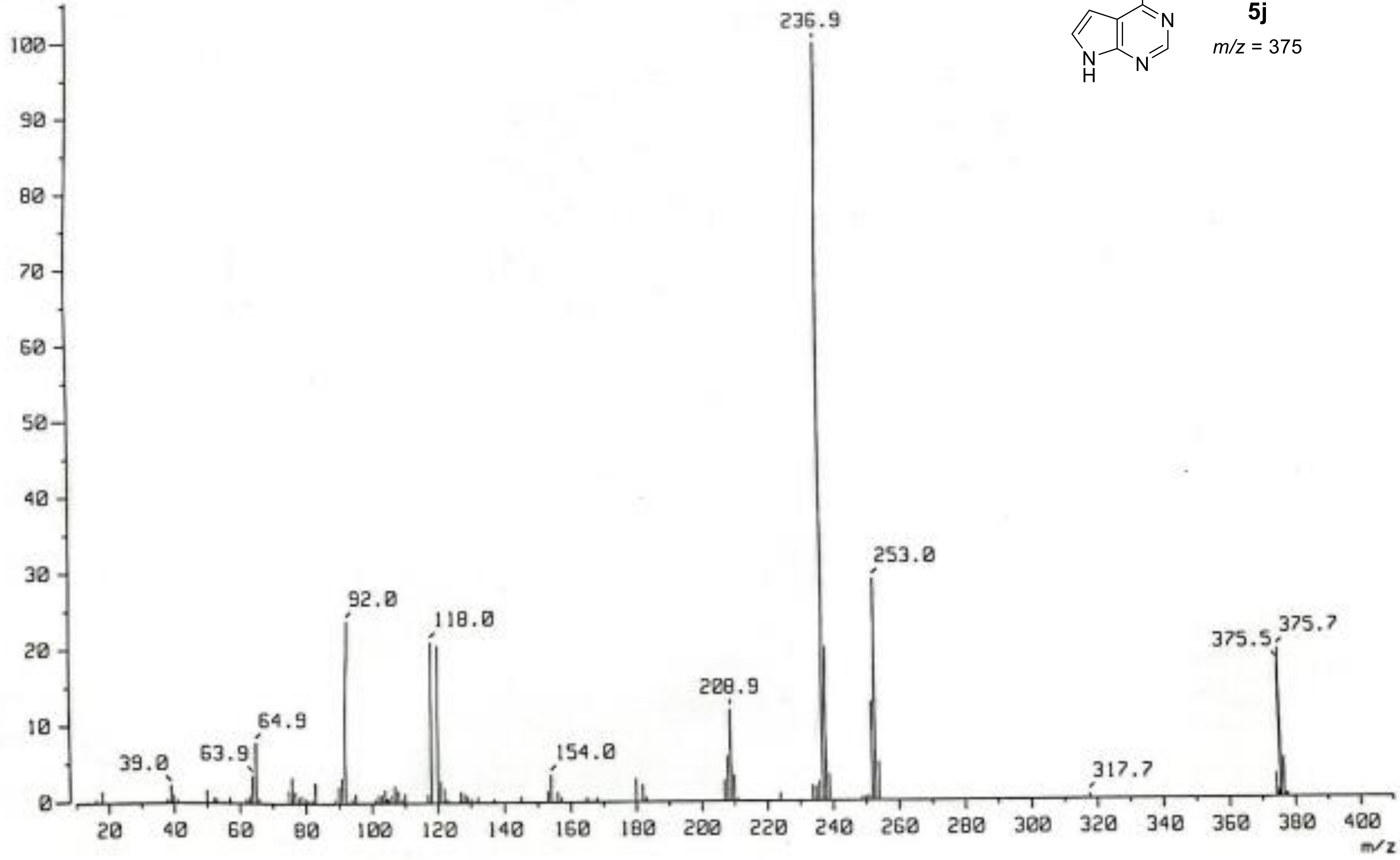

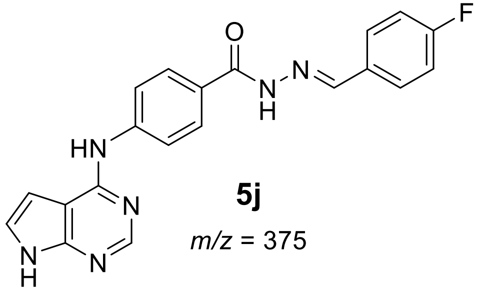

5j

m/z = 375

^{1}H-NMR spectra of **5k**

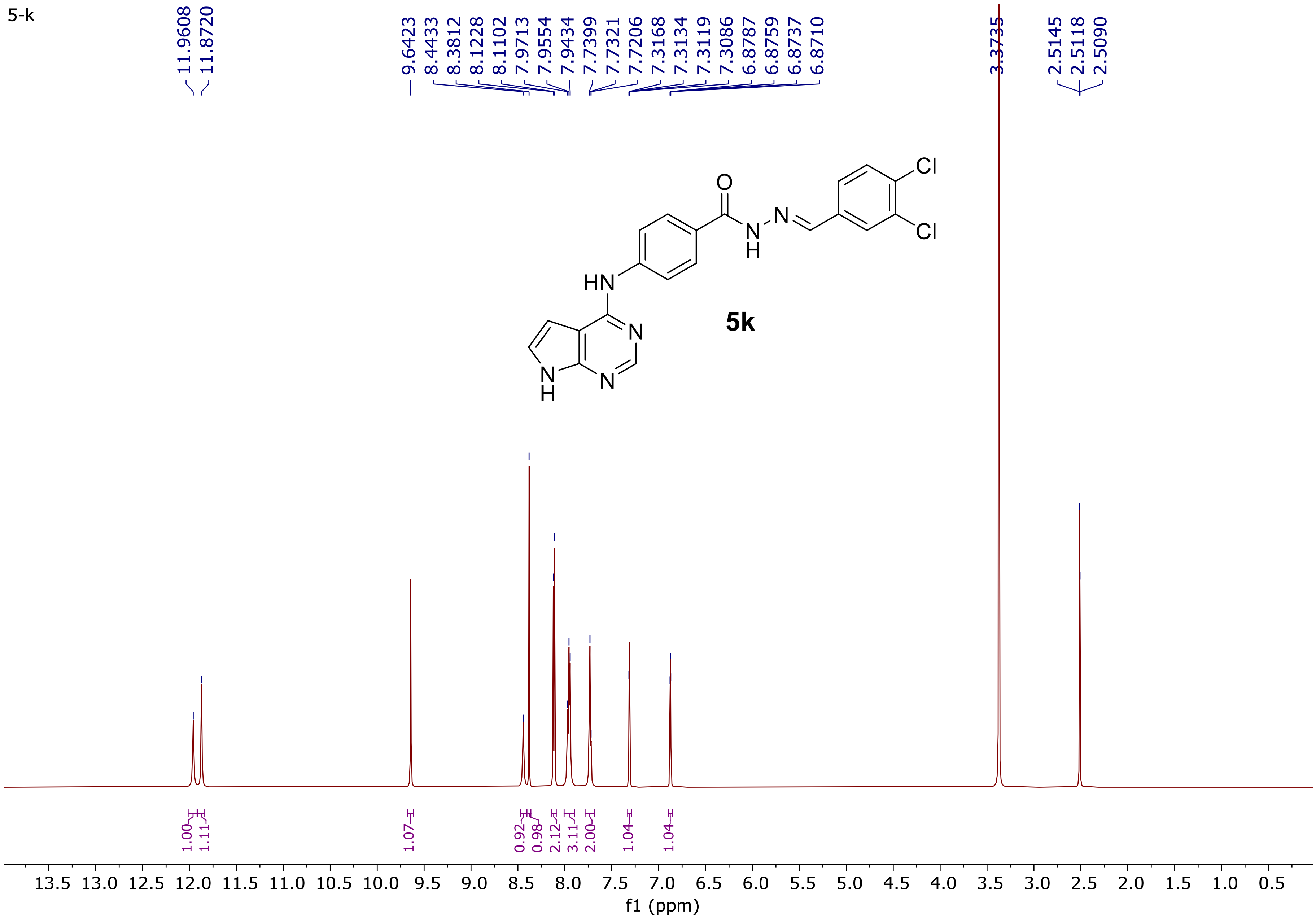

^{13}C-NMR spectra of **5k**

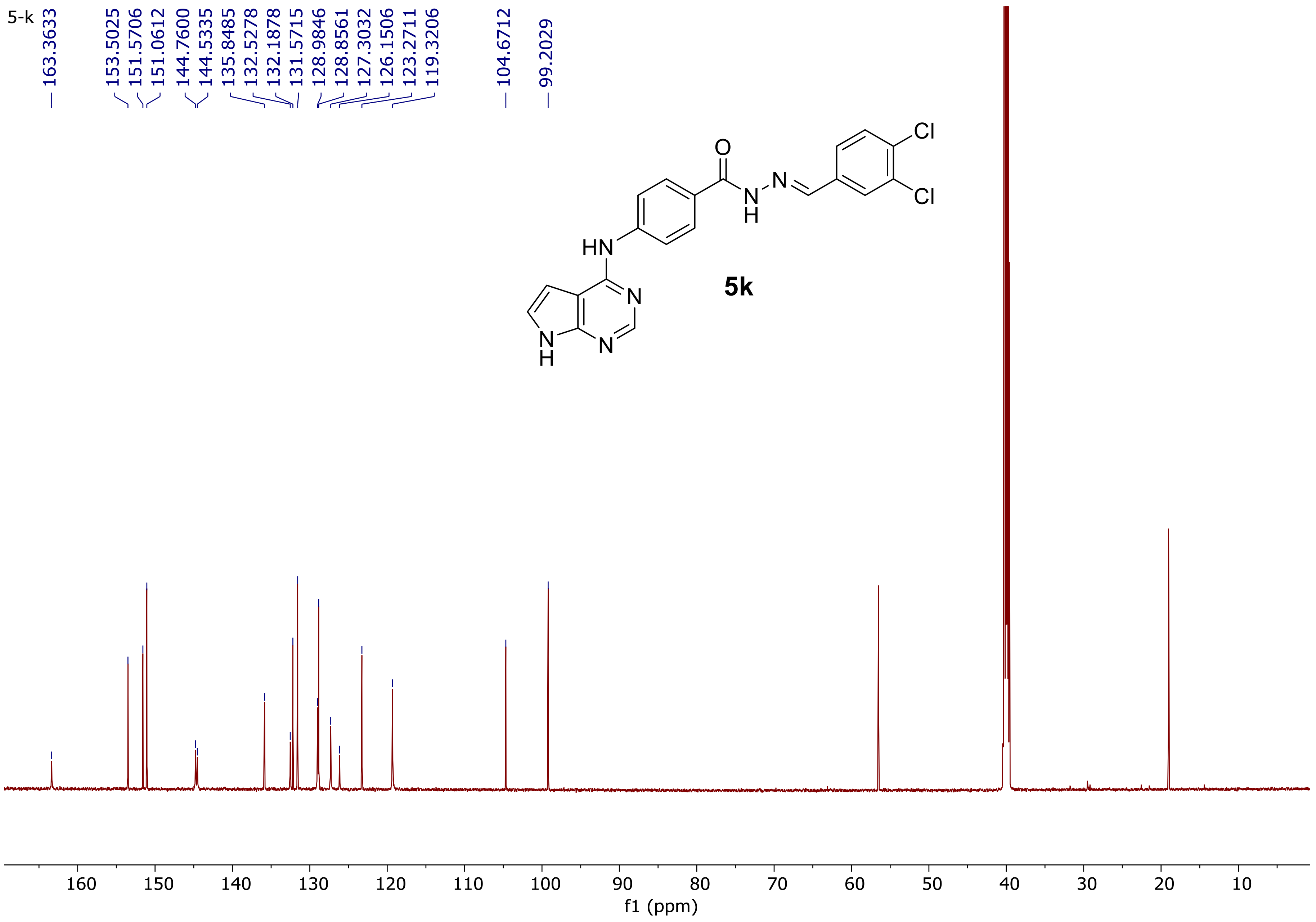

Mass spectra of **5k**

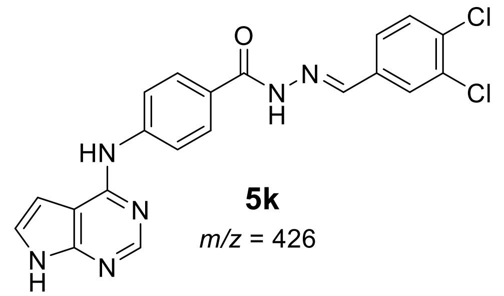

5k

m/z = 426

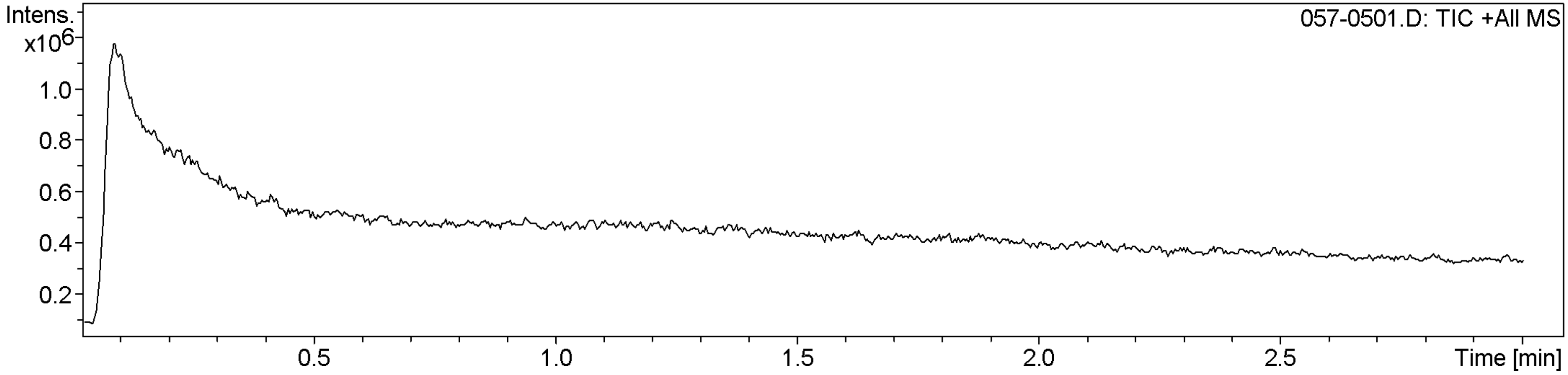

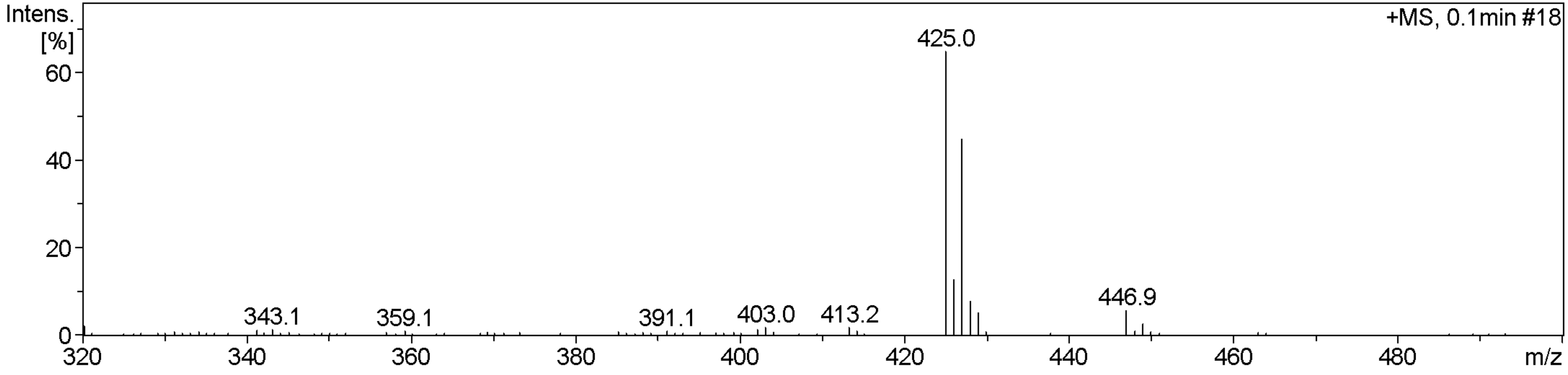

HMRS Mass spectra of **5k**

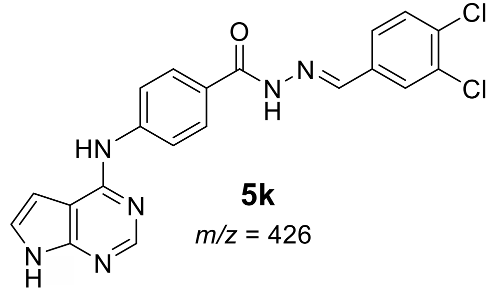

5k

m/z = 426

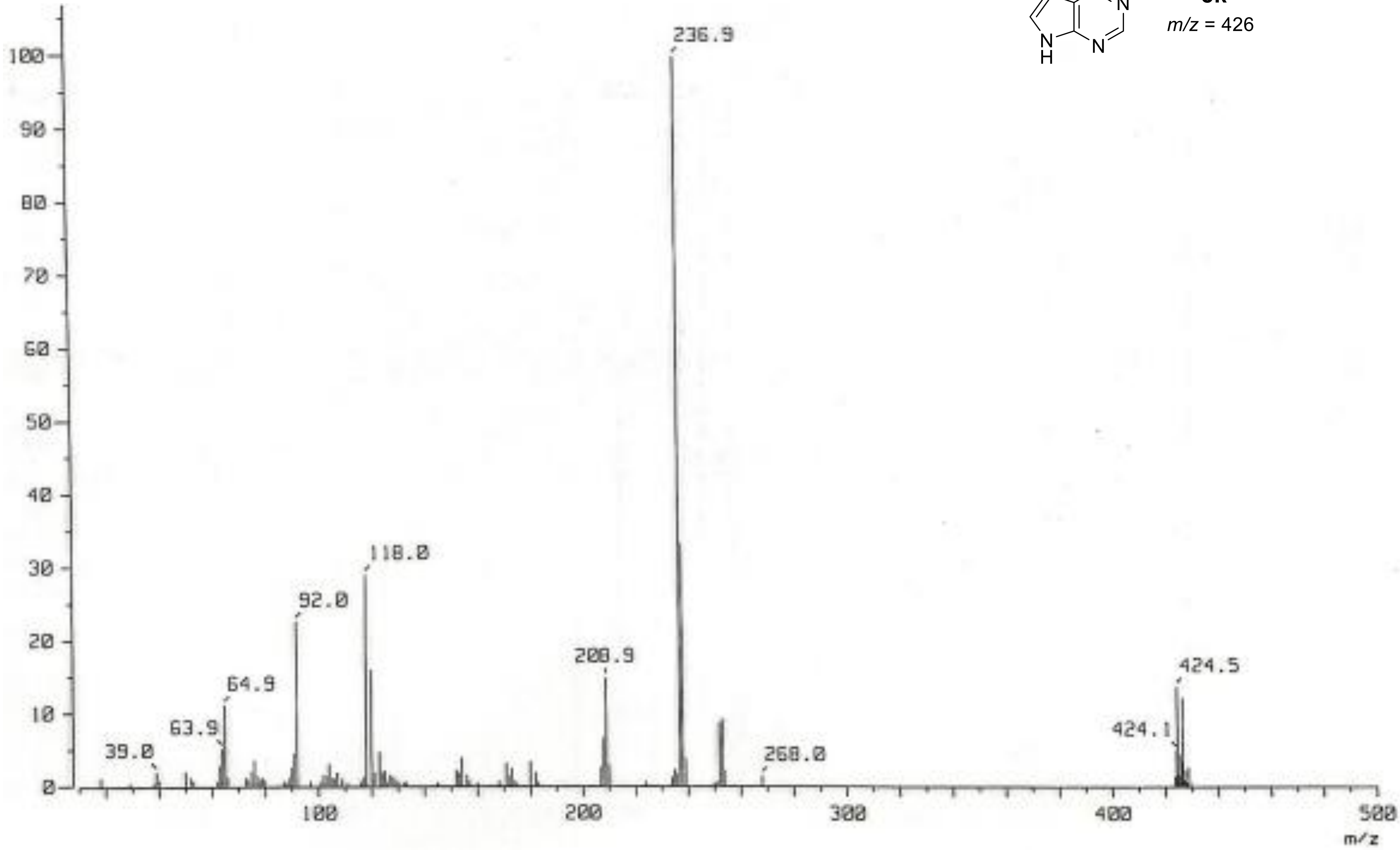

^{1}H-NMR spectra of **5l**

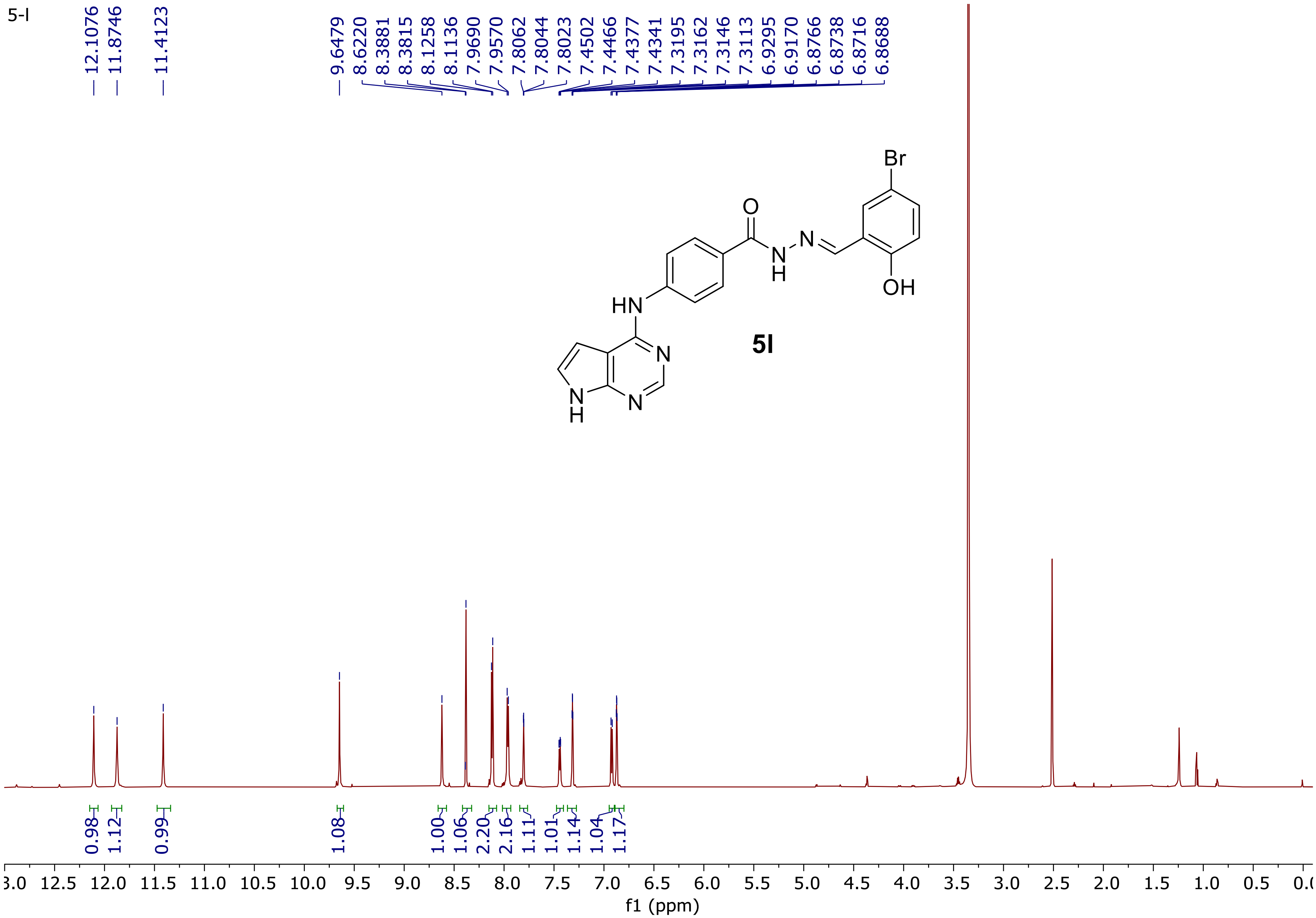

^{13}C-NMR spectra of **5l**

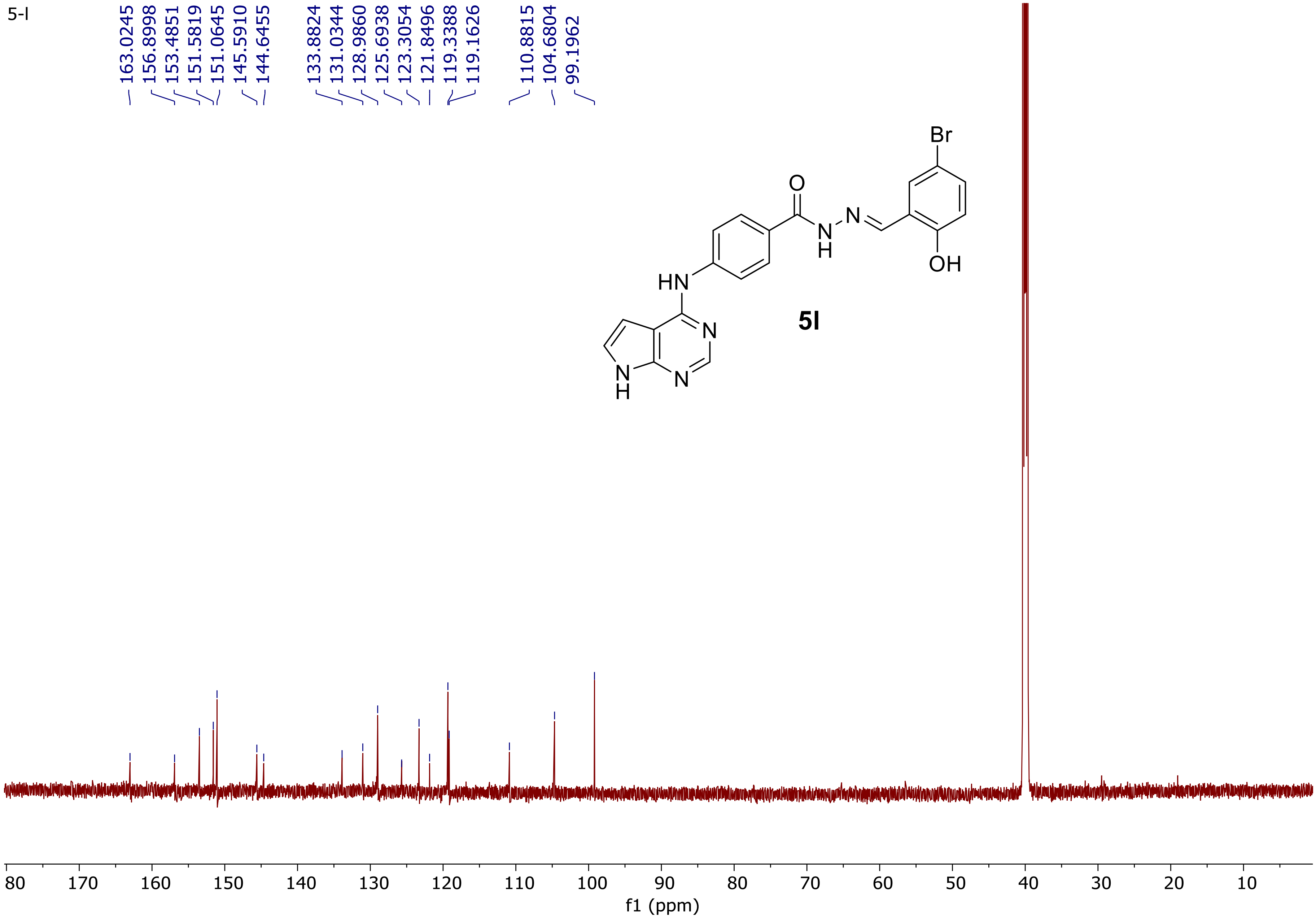

Mass spectra of **5l**

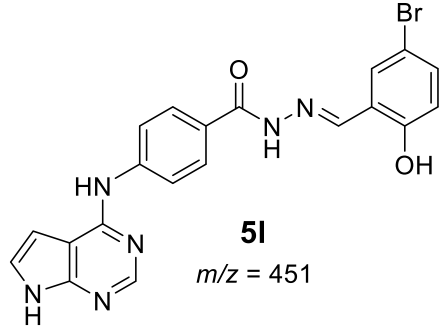

5l

m/z = 451

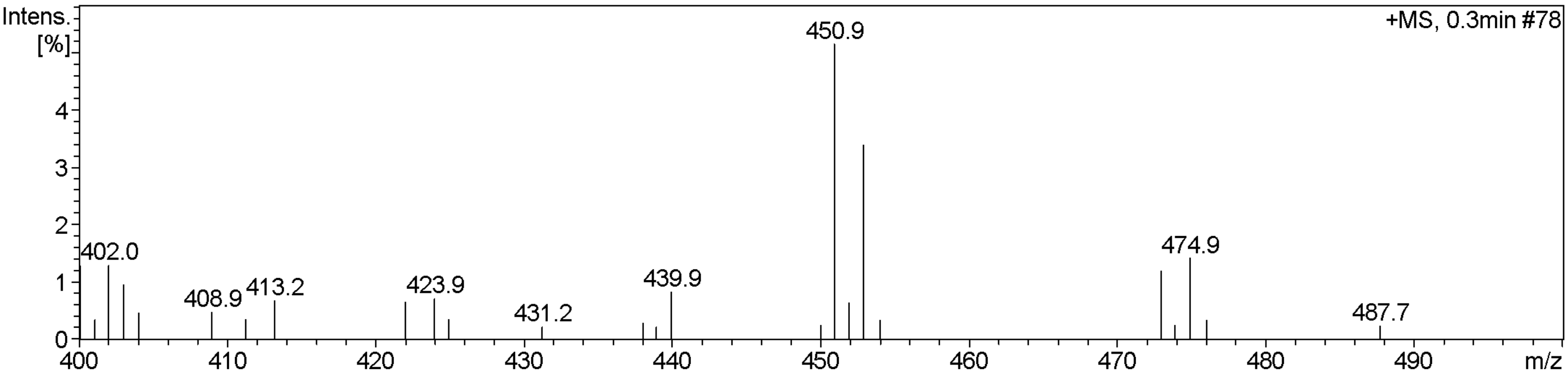

HMRS Mass spectra of **5l**

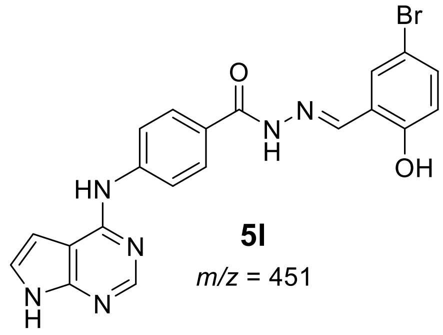

5l

m/z = 451

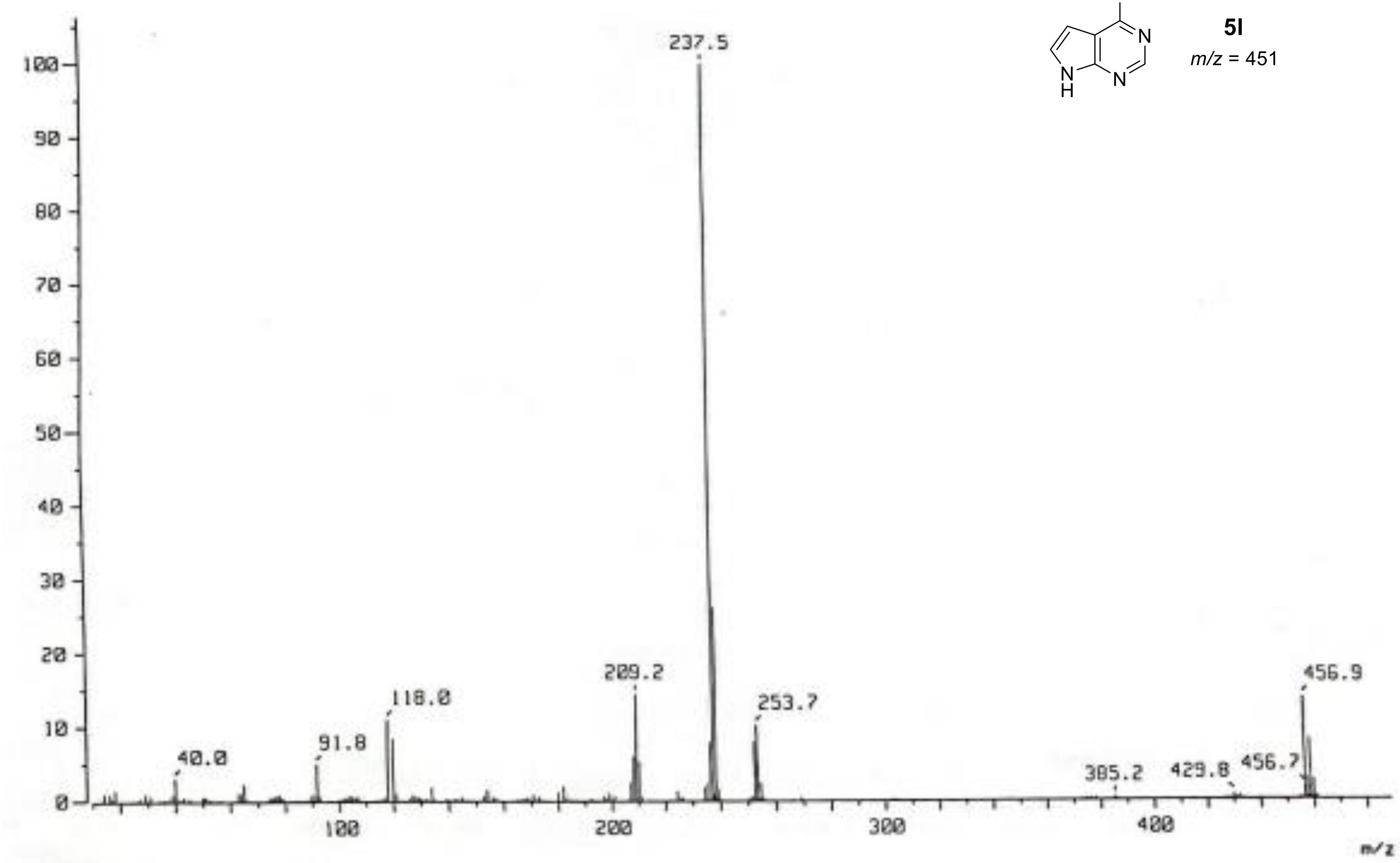

^{1}H-NMR spectra of **5m**

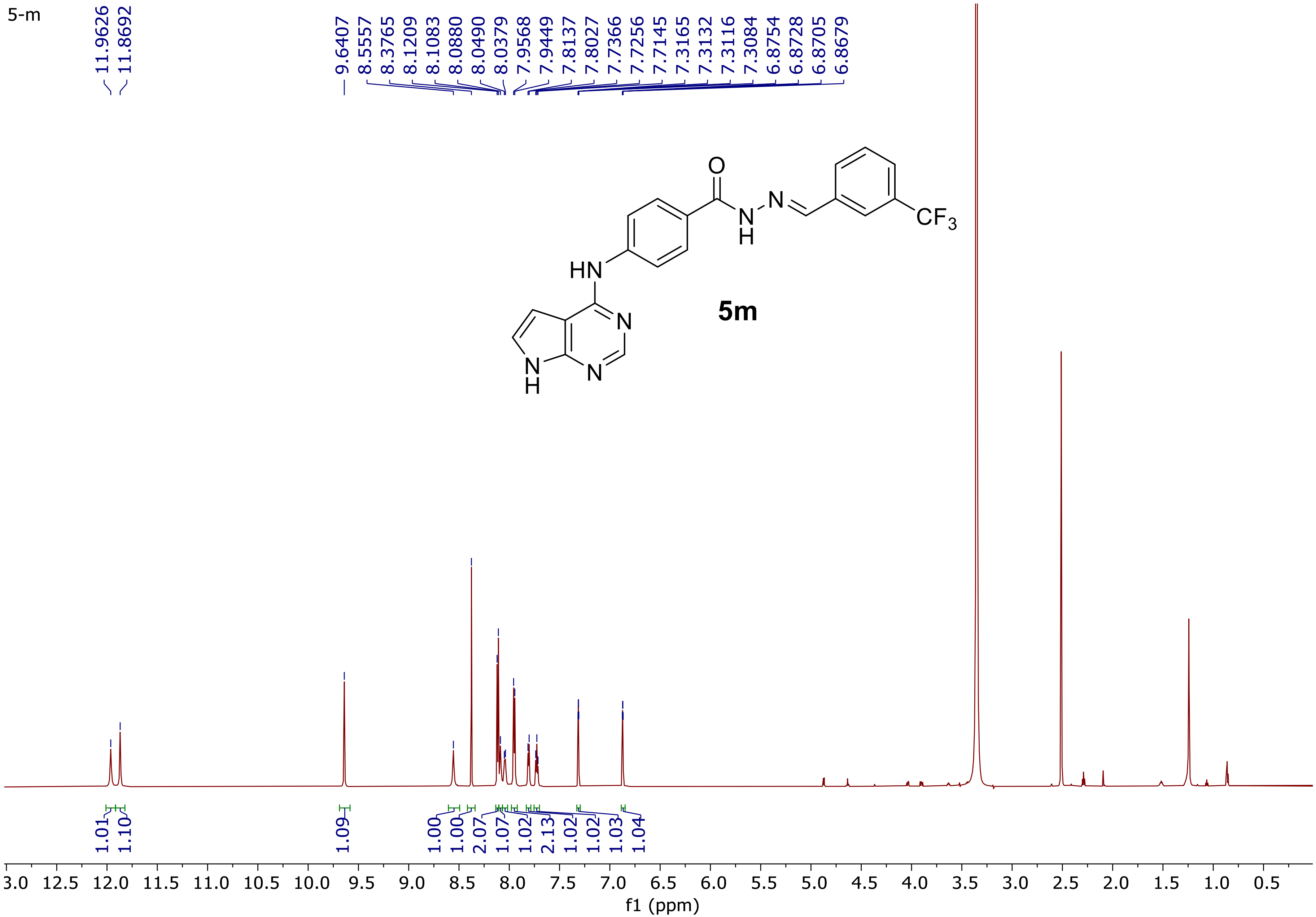

^{13}C-NMR spectra of **5m**

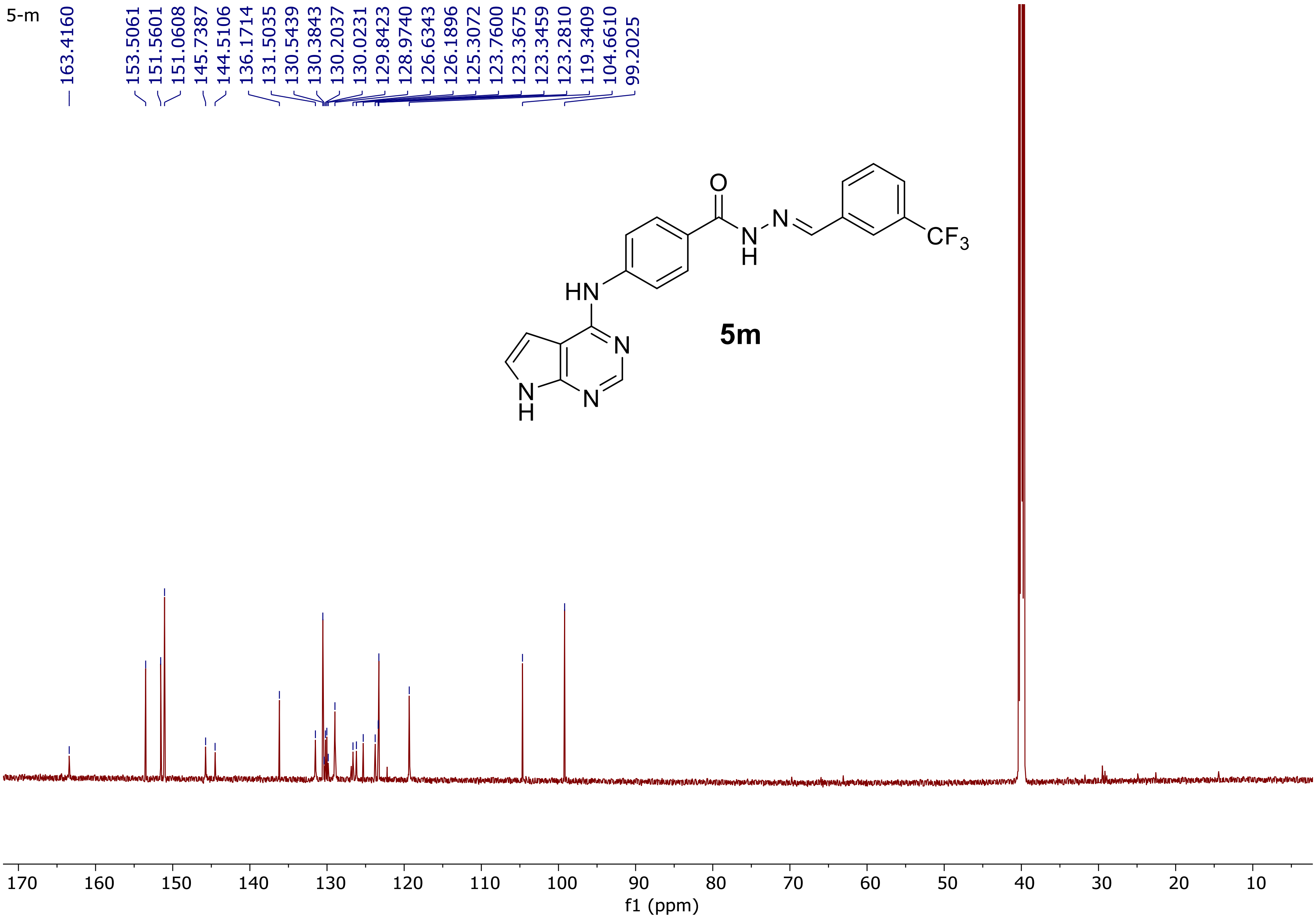

Mass spectra of **5m**

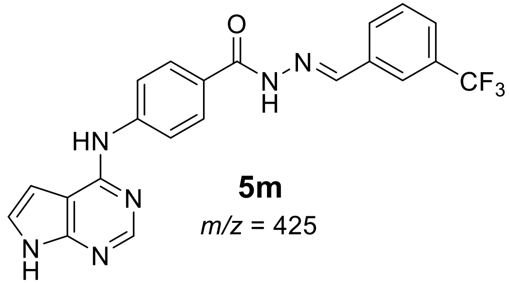

5m

m/z = 425

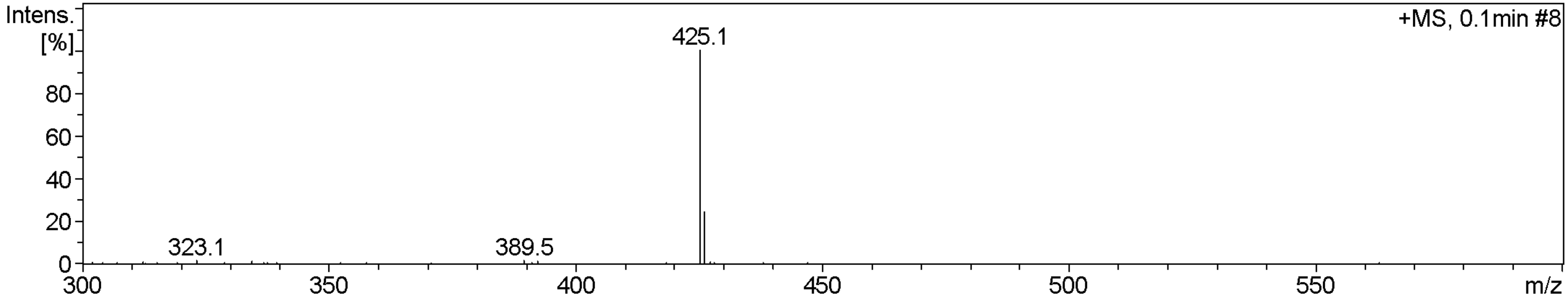

HMRS Mass spectra of **5m**

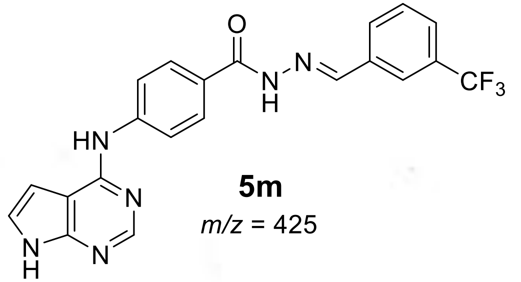

5m

m/z = 425

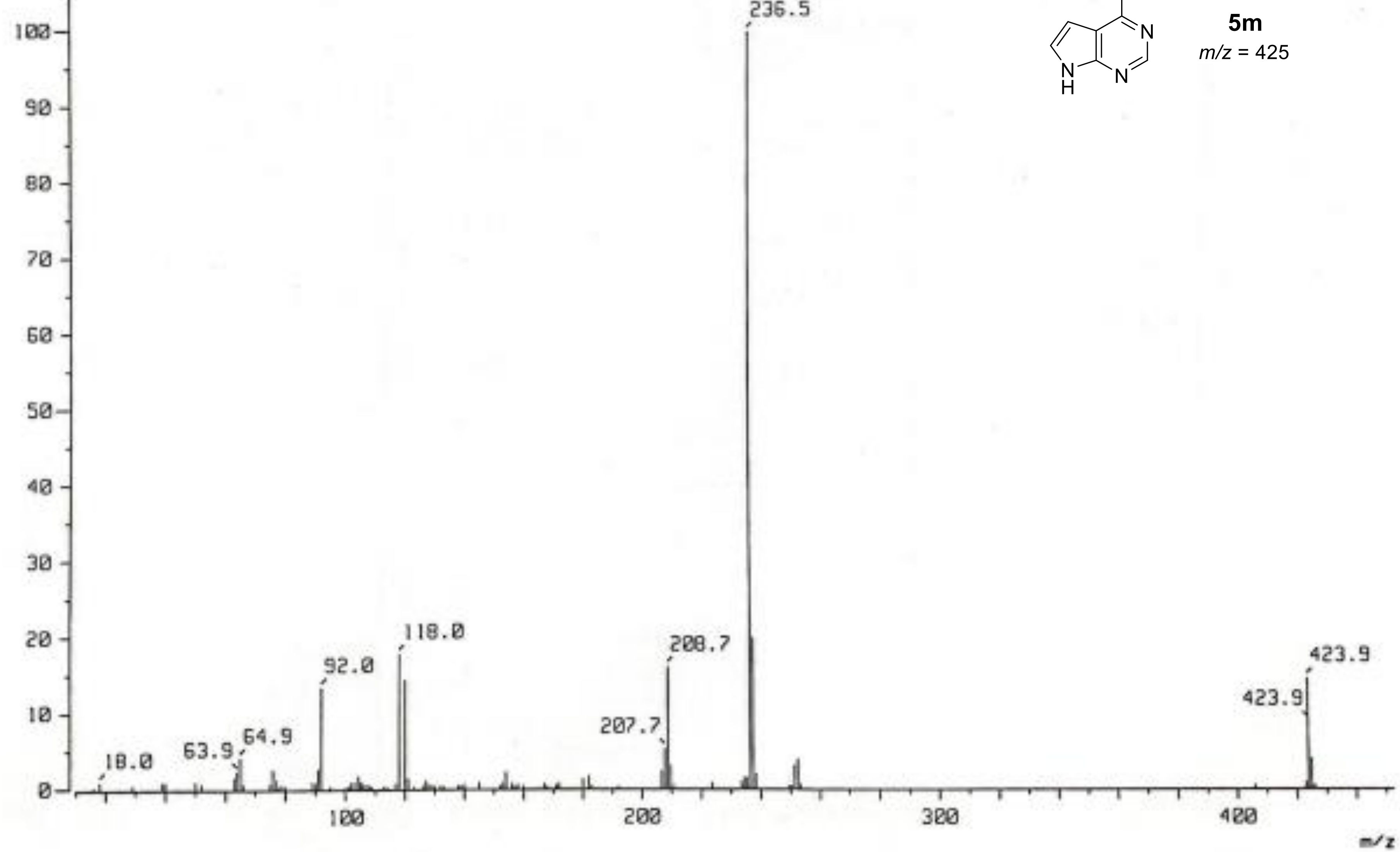

Supplement: Supplementary file 1 [file pharmaceuticals-16-01324-s001.zip › pharmaceuticals-2573695-supplementary.pdf]
